# Photoconductive Properties and Electronic Structure in 3,5-Disubstituted 2-(2′-Pyridyl)Pyrroles Coordinated to a Pd(II) Salicylideneiminate Synthon

**DOI:** 10.1021/acs.inorgchem.0c02991

**Published:** 2021-06-14

**Authors:** Andreea Ionescu, Nicolas Godbert, Roberto Termine, Massimo La Deda, Mario Amati, Francesco Lelj, Alessandra Crispini, Attilio Golemme, Mauro Ghedini, Pilar Garcia-Orduña, Iolinda Aiello

**Affiliations:** † MAT-INLAB (Laboratorio di Materiali Molecolari Inorganici) and LASCAMM-CR INSTM, Unità INSTM della Calabria, Dipartimento di Chimica e Tecnologie Chimiche, Università della Calabria, 87036 Arcavacata di Rende (CS), Italy; ‡ CNR NANOTEC-Istituto di Nanotecnologia U.O.S. Cosenza, 87036 Arcavacata di Rende (CS), Italy; § Dipartimento di Scienze and LASCAMM-CR INSTM, Unità INSTM della Basilicata, Università della Basilicata, 85100 Potenza, Italy; ⊥ Dipartimento di Fisica, Università della Calabria, 87036 Arcavacata di Rende (CS), Italy; # Instituto de Síntesis Química y Catálisis Homogénea, Universidad de Zaragoza-CSIC, Pza. San Francisco s/n, Zaragoza 50009, Spain

## Abstract

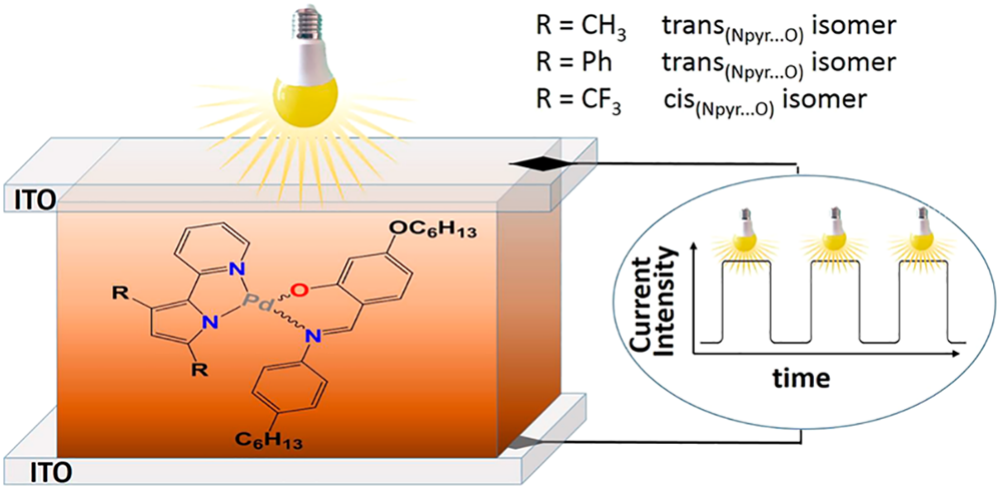

The synthesis and the electrochemical, photophysical, structural, and photoconductive properties of three new heteroleptic Pd(II) complexes with various 3′,5′- disubstituted-2-(2′-pyridil) pyrroles H(N^N) as coordinated ligands are reported. The coordination of the metal center was completed by a functionalized Schiff base H(O^N) used as an ancillary ligand. The [(N^N)Pd(O^N)] complexes showed highly interesting photoconductive properties which have been correlated to their electronic and molecular structures. Theoretical density functional theory (DFT) and time-dependent DFT calculations were performed, and the results were confronted with the organization in crystalline phase, allowing to point out that the photoconductive properties are mainly a consequence of an efficient intramolecular ligand-to-metal charge transfer, combined to the proximity between the central metal and the donor moieties in the solid-state molecular stacks. The reported results confirm that these new Pd(II) complexes form a novel class of organometallic photoconductors with intrinsic characteristics suitable for molecular semiconductors applications.

## Abstract 2

The first evidence of enhancement of the role of d* orbitals in improving photoconduction in crystalline Pd(II) complexes comprising 2-(2′-pyridyl)pyrroles.

## Introduction

Photoconductivity, that is, the increase of electrical conductivity of a material upon irradiation at a proper wavelength, is a complex phenomenon that can take place only if the charge carrier generation is stimulated by absorbed light, and separated charges can drift under the effect of an electric field.^1^ High photogeneration efficiency is therefore a key prerequisite of effective photoconductors.^1^

The study of new materials with optoelectronic properties, such as charge photoconduction and/or photogeneration, is of relevant contemporary interest, especially in the case of organic-based photoconductors, because of their high mechanical flexibility and processability and of the easy tuning of their properties via opportune chemical modifications.^2^ In particular, photoconductors are gaining attention for their applications in sensors, transistors, photodetectors, photorefractive composites, photovoltaic cells, light-emitting devices, and memory elements.^3^ To date, the most studied materials are based on oligomers,^4^ polymers,^5,6^ or composite systems.^7,8^ Although the performances of organic materials are still low if compared with their inorganic counterpart, intensive studies are on the way to reduce drawbacks, such as low photogeneration efficiency and/or low charge mobilities, that are often intrinsic characteristics of organic semiconductors. In order to reduce the gap between the performances of organic and inorganic semiconductors, different strategies have been adopted. In particular, materials with a more defined molecular and supramolecular architecture have recently attracted research interest. For example, discotic liquid crystals have received particular attention as semiconducting materials due to their columnar organization, which enhances charge mobility.^9^ Recently, we investigated the photoconductive properties of discotic metallomesogens, particularly interesting materials because of the presence of the metal center that allows intense absorptions over a wide spectral range.^10,11^

**Chart 1:**
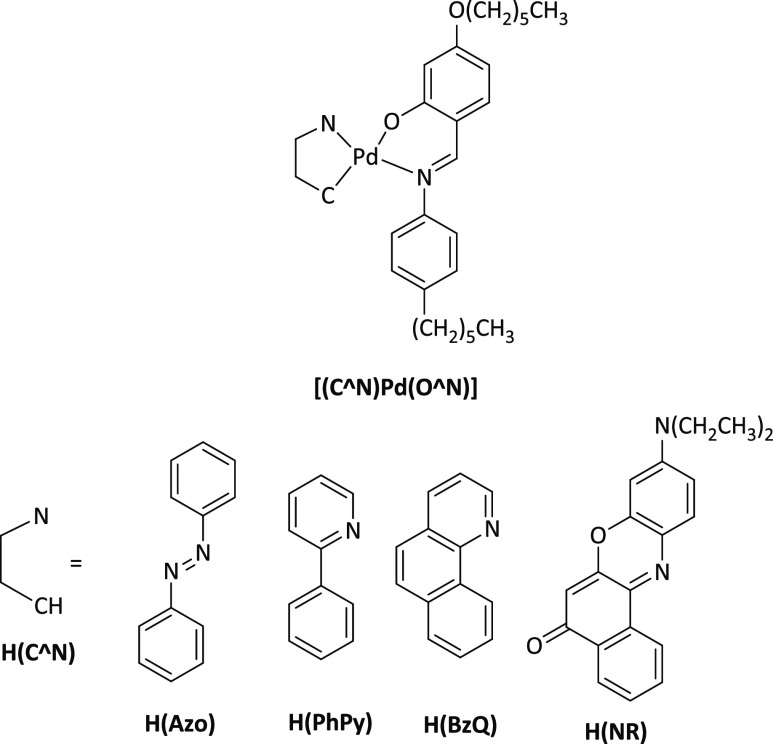
Structural Formulae of Previously Studied Photoconductive Square-Planar Cyclopalladated Complexesa

In the case of cyclometalated complexes, we have previously studied the photoconductive properties of square-planar Pd(II) complexes with a cyclopalladated azobenzene, benzo[*h*]quinoline, Nile Red or 2-phenylpyridine ligand H(C^N), and a Schiff base as ancillary ligand H(O^N) (Chart 1).^11,12^

a Adapted with permission from ref (11). Copyright 2012 Royal Society of Chemistry. Adapted with permission from ref (12). Copyright 2000 Elsevier.

In particular, these [(C^N)Pd(O^N)] complexes exhibited interesting photorefractive properties even without the addition of any dopant.^13−16^ The properties of these [(C^N)Pd(O^N)] complexes were studied in detail in order to understand the molecular mechanisms leading to their appealing optoelectronic properties^10,16−19^ that have been proven to be an issue from their specific chemical structure.^20^ Indeed, density functional theory (DFT) studies showed that for all of these [(C^N)Pd(O^N)] complexes, the HOMO orbital is mainly localized on the Schiff base and the LUMO orbital on the cyclometalated ligand. Hence, the two frontier orbitals HOMO and LUMO are, in these complexes, physically separated by the metal center; moreover, DFT studies have highlighted a distortion of the square-planar geometry around the metal center during excitation. Thus, the photogeneration of charge carriers might be associated with the spatial separation of HOMO and LUMO, and an additional contribution could derive from the conformational twisting of the excited state that probably delays or hampers charge recombination. Within this frame, with the intent to (i) enlarge the pool of available photoconductive Pd(II) complexes, (ii) improve the efficiency of photogeneration or photoconduction, and (iii) deepen the understanding between the structure–property relationship, we herein synthesized and studied three new Pd(II) complexes bearing, instead of the previously used H(C^N) cyclometalated ligands, coordinated 3′,5′-disubstituted-2-(2′-pyridyl)pyrrole ligands, H(N^N).^1−3^ The H(O^N) Schiff base ancillary ligand, with respect to our previous study, was kept unvaried (Chart 1)^20^ for immediate comparison with the properties displayed by the newly synthesized complexes. The choice of the H(N^N) ligands derives from their known versatile chelating abilities, displayed against different metal ions, as well as their behavior as monoanionic bidentate ligand analogous to H(C^N).^21^ In addition, it is possible to modify the chemical structure of H(N^N) ligands by introducing specific substituents with different electronic and/or steric characteristics in the 3,5- positions of the pyrrolic fragment.^22^ To this end, the three different substituents CH_3_, Ph, and CF_3_ were selected, and their influence on the photoconductive properties of the resulting complexes was thoroughly investigated.

## Experimental Section

### General Information

All commercially available starting materials and Pd(II) acetate (Sigma-Aldrich) were used without further purification. The H(N^N)^1^,^22^ H(N^N)^2^,^22^ H(N^N)^3^,^21i^ and H(O^N)^12^ ligands and [(N^N)^1^Pd(μ-OAc)]_2_**I**,^21i^ [(N^N)^2^Pd(μ-OAc)]_2_**II**,^21l^ and [(N^N)^3^Pd(μ-OAc)]_2_**III**^21i^ complexes were prepared following literature methods.

Elemental analyses were carried out on a CHNS/O analyzer PerkinElmer 2400. FT-IR spectra were recorded on a PerkinElmer 2000 FT-IR instrument. ^1^H NMR spectra were recorded on a Bruker WM-300 (CDCl_3_ solution, internal standard Me_4_Si). The 2D-NOESY ^1^H NMR spectrum of complex **2** was recorded on a Bruker WM-3500 on a degassed (N_2_) CDCl_3_ solution with internal standard Me_4_Si.

Cyclic voltammograms were recorded using an Epsilon ECI2 potentiostat at a standard scan rate of 100 mV s^–1^, in a ca. 3 mL solution of freshly distilled, anhydrous, and degassed dimethylformamide, and electrochemical measurements were conducted under argon atmosphere. [N(C_4_H_9_)_4_]PF_6_ was used as a supporting electrolyte (0.1 M). A platinum wire was used as a counter electrode, a platinum disk as a working electrode, and a silver wire as a pseudoreference electrode. All the reported oxidation and reduction potentials are relative to ferrocene/ferrocenium (Fc/Fc^+^), using the voltammetric oxidation of ferrocene added in the analytical solution and used as internal reference. Frontier orbitals energies, HOMO and LUMO, were estimated by assuming for ferrocene the energy value of −4.8 eV, as previously reported.^23−25^

### Photophysics Measurements

Spectrofluorimetric grade solvents were used for the photophysical investigations in solution, at room temperature. A PerkinElmer Lambda 900 spectrophotometer was employed to obtain the absorption spectra. Steady-state emission spectra were recorded on a HORIBA Jobin-Yvon Fluorolog-3 FL3-211 spectrometer equipped with a 450 W xenon arc lamp, double-grating excitation, single-grating emission monochromators (2.1 nm/mm dispersion; 1200 grooves/mm), and a Hamamatsu R928 photomultiplier tube, while a TBX-04-D single-photon-counting detector was used for time-resolved measurements. Emission and excitation spectra were corrected for source intensity (lamp and grating) and emission spectral response (detector and grating) by standard correction curves. Measurements at 77 K were conducted by employing capillary tubes immersed in liquid nitrogen and hosted within homemade quartz Dewar. Time-resolved measurements were performed using the time-correlated single-photon counting option on the Fluorolog 3. NanoLED at 265 nm, fwhm <1.0 ns with repetition rate at 1 MHz, was used to excite the sample. Excitation sources were mounted directly on the sample chamber at 90° to a single-grating emission monochromator (2.1 nm/mm dispersion; 1200 grooves/mm) and collected with the TBX-04-D single-photon-counting detector. The photons collected at the detector are correlated to the excitation pulse by a time-to-amplitude converter (TAC). Signals were collected using an IBH Data Station Hub photon-counting module, and data analysis was performed using the commercially available DAS6 software (HORIBA Jobin Yvon IBH). Goodness of fit was assessed by minimizing the reduced chi squared function (χ^2^) and visual inspection of the weighted residuals.

#### Synthesis of [(N^N)_1–3_Pd(BS)] complexes (**1**–**3**)

##### [(N^N)^1^Pd(O^N)] (**1**)

To a suspension of the intermediate **I** (0.10 g, 0.15 mmol) in ethanol (20 mL) was added the ligand H(O^N) (0.11 g, 0.30 mmol). The resulting mixture was stirred at room temperature for 6 h. The obtained yellow-orange solid was collected by filtration and purified by dissolving it in the minimum amount of chloroform and precipitated with ethanol. Yield (from **I**) 76% (0.15 g). Mp 146 °C. Anal. calcd for C_36_H_45_N_3_O_2_Pd (658.18): C, 65.69; H, 6.89; N, 6.38%. Found: C, 65.30; H, 6.83; N, 6.12%. FT-IR (KBr, cm^–1^) ν_max_: 2927, 2855, 1603, 1544, 1491, 1465, 1354, 1202, 1122, 970, 814, 770, 735. ^1^H NMR (CDCl_3_, ppm) δ: 8.52 (1H, dd, *J* = 6 Hz, *J* = 0.5 Hz, H_6′_), 7.78 (1H, s, H_b_), 7.30 (2H, d, *J* = 8 Hz, H_e-d_), 7.54 (ddd, 1H, *J* = 8 Hz, *J* = 6 Hz, *J* = 1.5 Hz, H_4′_), 7.25–7.15 (2H, m, H_c_ and H_3′)_, 7.09 (2H, *J* = 8 Hz, H_f-g_), 6.75 (1H, ddd, *J* = 8 Hz, *J* = 6 Hz, *J* = 1.2 Hz, H_5′_), 6.49 (1H, d, *J* = 1.8 Hz, H_a_), 6.31 (1H, dd, *J* = 8.7 Hz, *J* = 2.4 Hz, H_b_), 5.44 (1H, s, H_4_), 4.01 (2H, t, *J* = 6.3 Hz, O–CH_2_), 2.58 (2H, t, *J* = 6 Hz, Ph–CH_2_), 2.28 (3H, s, CH_3_(pyr)), 1.81(2H, q, *J* = 6 Hz, O–CH_2_–CH_2_) 1.55–1.25 (CH_2_ alkyl chains),1.15 (3H, s, CH_3_(pyr)), 0.92 (3H, t, *J* = 7 Hz, CH_3_), 0.88 (3H, t, *J* = 6 Hz, CH_3_).

##### [(N^N)^2^Pd(O^N)] (**2**)

According to the above-mentioned typical procedure, complex **2** was prepared from intermediate **II**. Yellow-orange solid. Yield (from **II**): 79% (0.13 g). Mp 163 °C. Anal. calcd for C_46_H_49_N_3_O_2_Pd (782.32): C, 70.62; H, 6.31; N, 5.37%. Found: C, 70.53; H, 6.39; N, 5.08%. FT-IR (KBr, cm^–1^) ν_max_: 2928, 2857, 1600, 1515, 1489, 1466, 1347, 1252, 1194, 1140, 776, 745, 700. ^1^HNMR (CDCl_3_, ppm) δ: 8.58 (1H, dd, *J* = 6 Hz, *J* = 0.5 Hz, H_6′_), 7.60–7.48 (m, 3H), 7.46–7.27 (m, 6H), 7.25–7.00 (m, 5H), 6.86 (1H, ddd, *J* = 8 Hz, *J* = 6 Hz, *J* = 1 Hz, H_5′_), 6.82 (2H, d, *J* = 8.4 Hz), 6.63 (2H, d, *J* = 8,4 Hz), 6.57 (1H, d, *J* = 2.4 Hz, H_a_), 6.31 (1H, dd, *J* = 8.7 Hz, *J* = 2.4 Hz, H_b_), 6.00 (1H, s, H_4_), 4.01 (2H, t, *J* = 6.5 Hz, O–CH_2_), 2.58 (2H, t, *J* = 6 Hz, Ph–CH_2_), 1.81(2H, q, *J* = 6 Hz, O–CH_2_–CH_2_) 1.55–1.25 (CH_2_ alkyl chains), 0.93 (3H, t, *J* = 7 Hz, CH_3_), 0.89 (3H, t, *J* = 6 Hz, CH_3_).

##### [(N^N)^3^Pd(O^N)] (**3**)

According to the above-mentioned typical procedure, complex **3** was prepared from intermediate **III**. Orange solid. Yield (from **III**): 21% (0.04 g). Mp 170 °C. Anal. calcd for C_36_H_39_N_3_O_2_F_6_Pd (766.12): C, 56.44; H, 5.13; N, 5.48%. Found: C, 56.22; H, 4.84; N, 5.09%. FT-IR (KBr, cm^–1^) ν_max_: 2928, 2857, 1611, 1591, 1503, 1465, 1295, 1135, 1100, 992, 776, 730. ^1^HNMR (CDCl_3_, ppm) δ: 7.58 (1H, dd, *J* = 7.5 Hz, *J* = 1 Hz, H_6′_), 7.53 (1H, s, H_b_), 7.50 (2H, d, *J* = 6 Hz, H_e-d_), 7.42 (ddd, 1H, *J* = 7.5 Hz, *J* = 7 Hz, *J* = 1.5 Hz, H_4′_), 7.12 (1H, d, *J* = 9 Hz, H_c_), 7.09 (2H, *J* = 6 Hz, H_f-g_), 6.88 (1H, s, H_4_), 6.82 (1H, dd, *J* = 6 Hz, *J* = 1.5 Hz, H_3′_), 6.64 (1H, d, *J* = 2.1 Hz, H_a_), 6.31 (1H, dd, *J* = 8.7 Hz, *J* = 2.4 Hz, H_b_), 6.29 (1H, ddd, *J* = 7 Hz, *J* = 6 Hz, *J* = 1.2 Hz, H_5′_), 4.02 (2H, t, *J* = 6.3 Hz, O–CH_2_), 2.58 (2H, t, *J* = 7.5 Hz, Ph–CH_2_), 1.81(2H, q, *J* = 7 Hz, O–CH_2_–CH_2_) 1.56–1.28 (CH_2_ alkyl chains), 0.92 (3H, t, *J* = 7 Hz, CH_3_), 0.89 (3H, t, *J* = 6 Hz, CH_3_).

### X-ray Crystallography

X-ray diffraction data for single crystals of complex **1** was collected using synchrotron radiation from the European Synchrotron Radiation Facility, Grenoble, France (ESRF) BM 16 CRG beamline. Data were measured in a single axis HUBER diffractometer, using silicon (111) monochromated synchrotron radiation (0.73780 Å). The crystals were cooled to 100(1)K with an Oxford 600 Cryosystem open-flow nitrogen cryostat. Intensities were integrated with the HKL2000 suite^26^ and absorption corrected with SORTAV program.^27,28^

Single-crystal XRD (SCXRD) data of **3** were collected at 100(1)K temperature with a Bruker-Nonius X8APEXII CCD area detector system equipped with a graphite monochromator with radiation Mo Kα (λ = 0.71073 Å). The data were processed through the SAINT^28^ reduction and SADABS^29^ absorption software. Both structures were solved by standard Patterson methods and refined by full matrix least-squares based on *F*^2^ by using the SHELX and SHELXTL structure determination program.^30^ All nonhydrogen atoms were refined anisotropically. Hydrogen atom positions were calculated geometrically and refined using the riding model. In the case of complex **3**, one, among the alkyl chains, of the carbon atoms of one of the three molecules of the asymmetric unit is found disordered in two positions and refined with occupancy factors of 0.6 and 0.4. Both sets of atoms have been refined anisotropically. Cell parameters and final refinement data are given in Table 1. Selected interatomic bond lengths and angles are given in Table 2. CCDC reference numbers are CCDC 1959674 (**1**) and 1959652 (**3**).

**Table 1:** Details of Data Collection and Structure Refinements for Complexes 1 and 3

complex	**1**	**3**
formula	C_36_H_45_N_3_O_2_Pd	C_36_H_39_F_6_N_3_O_2_Pd
*M*_r_	658.20	766.10
crystal system	monoclinic	triclinic
space group	*P*2_1_/*c*	*P*-1
a [Å]	40.8279(6)	17.4620(9)
b [Å]	9.36210(10)	17.5169(9)
c [Å]	16.6972(2)	19.9749(11)
α [°]	90	72.2680(10)
β [°]	100.5770(6)	70.3980(10)
γ [°]	90	64.3360(10)
V [Å^3^]	6273.81(14)	5095.9(5)
Z	8	6
ρ calcd [gcm^–3^]	1.394	1.498
μ [mm^–1^]	0.684	0.616
θ range [°]	2.32–29.46	1.51–26.37
data collected	60280	46578
unique data, *R*_int_	13691, 0.0642	20377, 0.0281
no. parameters	766	1306
*R*_1_ [obs. data]	0.1302	0.0425
*wR*_2_ [all data]	0.3341	0.1199
GOF	1.181	1.092

**Table 2:** Relevant Bond Lengths (Å) and Angles (°) for Complexes **1** and **3**

	**1**	**3**
Pd(1)–N(1)	2.022(10), 2.029(10)	2.060(3), 2.038(3), 2.028(3)
Pd(1)–N(2)	2.042(10), 2.026(10)	2.026(3), 2.025(3), 2.035(3)
Pd(1)–N(3)	2.054(10), 2.034(11)	2.026(3), 2.018(3), 2.024(3)
Pd(1)–O(1)	2.000(8), 1.988(8)	1.964(2), 1.968(3), 1.978(3)
N(1)–Pd(1)–N(3)	172.8(4), 172.2(4)	100.7(1), 97.4(1), 97.3(1)
N(1)–Pd(1)–N(2)	80.0(4), 81.1(4)	79.7(1), 80.6(1), 80.2(1)
N(1)–Pd(1)–O(1)	86.3(4), 86.2(4)	169.8(1), 173.0(1), 167.5(1)
N(3)–Pd(1)–N(2)	102.4(4), 101.5(4)	177.3(1), 173.9(1), 169.9(1)
N(3)–Pd(1)–O(1)	91.7(4), 91.6(4)	88.8(1), 88.7(1), 89.4(1)
N(2)–Pd(1)–O(1)	165.7(4), 166.6(4)	90.7(1), 93.6(1), 95.0(1)

### Computational Methods

All the reported calculations (except the ONIOM computations detailed below in this paragraph) were performed on model complexes obtained from **1**, **2**, and **3** by replacing the O^N ligand hexyl chains with methyl groups. All the *ab initio* computations were based on DFT methods. The used xc functionals were the meta-hybrid M06^31^ and, in order to be comparable with our previous studies,^20a^ the hybrid modified one parameter mPW1PW91^32^ xc. For all second period atoms, the Dunning all electron basis set augmented by a set of d polarization functions (D95(d))^33^ were used on C, N, O, F, and H atoms. For Pd, the double-ζ Stuttgart basis set including f polarization functions and relativistic effects with a fully relativistic small core pseudopotential (SDD09)^34^ were used rather than the default SDD as included in Gaussian 09. The ultrafine option with (99 radial, 590 angular) grid points was used for the integral calculations for all atoms except Pd, where a total of (15,662,28) grid points were used. All energy minimized structures were characterized by the calculation of the Hessian matrix in order to check that they were minima and not simple stationary points on the molecular Born–Oppenheimer energy surface. Singlet and triplet excitations were computed with the time-dependent (TD)-DFT linear response approach in the random phase approximation. NMR chemical shifts were computed with the gauge-independent atomic orbital (GIAO) method.^35^ The first and second triplet states geometries were computed at the TD-DFT level with use of the MPW1PW91 xc functional.

ONIOM computations^36^ were performed after extracting six molecules from the resolved **1** crystal without applying, in this case, any simplification of their structure so to model more accurately the packing interactions among molecules in the crystal. The central molecule in this cluster was treated as the high-level layer, with the MPW1PW91 xc functionals and the basis set/pseudopotential combination described above. The remaining (surrounding) molecules were treated at the PM6 level. Tests were performed on the PM6 performances on complex **1** (see SI). All the structure optimizations, UV–vis characterizations, and spin densities of charged molecules were computed by using the Gaussian 09 rev. D.01 software.^37^

The solvent was simulated according to the polarizable continuum model^38^ as implemented in Gaussian 09.^39^ DFT comparison between the computed and experimental redox potentials was performed, with the mPW1PW91/D95(d)/SDD09/THF DFT computed HOMO and LUMO shifting by −498.1 and −350.4 mV, respectively, to account for the Fe/Fe^+^ reference and further contribution due to non-Nernstian phenomena on the electrodes, computational electron correlation, and thermodynamic effects. The HOMO and LUMO values were computed as the energy difference between neutral and charged complexes after geometry optimization of all the structures in the presence of the solvent (PCM model as reported above).

Singlet and triplet emission in solution at 77 K was computed through TD-DFT structure optimization in the excited state as implemented in the Gaussian 09 rev. D01 software. These optimizations were performed in solution by means of the above-discussed PCM method. With the aim to model the hindrance to the full geometry relaxation due to the frozen environment in the solid solutions, excited-state optimizations were performed by freezing all the dihedral angles of the molecule at their computed ground-state values in solution (PCM). Without this tool, an unlikely strong distortion toward a not-planar coordination sphere is computed, especially in T_1_ (see the discussion about ONIOM computations in the SI).

Part of the metal–ligand bond analysis reported in the paper and detailed in the SI was obtained from the application of the Amsterdam Density Functional (ADF) software. The methods used are reported in the SI, where additional information is also discussed.

### Photoconductivity Measurements

The cells for photoconductivity experiments were prepared by overlapping two indium–tin oxide (ITO) covered glasses, controlling the thickness using 2 μm glass spacers. The two substrates were glued together with epoxydic glue. The actual cell thicknesses were controlled by interferometry. The finished cells were filled by capillarity on a hot plate at a temperature slightly above the melting point. Photocurrent data were taken by applying a DC voltage and then measuring the current in the dark and under illumination. The voltage application and the current measurement were carried out by using a Kethley 6517A electrometer. In order to minimize the effects due to light intensity gradients and to charge trapping, measurements were carried out at wavelengths for which the absorption is very low, but still high enough to be able to detect a clear photoresponse. As the three complexes exhibit an absorption edge at different wavelengths, the light was provided by a He–Ne laser at 633 nm for complex **2** and by a solid-state laser at 532 nm for complexes **3**. In the case of complexes **1**, measurements were carried out at 600 nm by using a lamp/monochromator system. For further details see SI and Figure S1.

## Results and Discussion

### Synthesis and Structures of [(N^N)_1–3_Pd(O^N)] Complexes

**Scheme 1:**
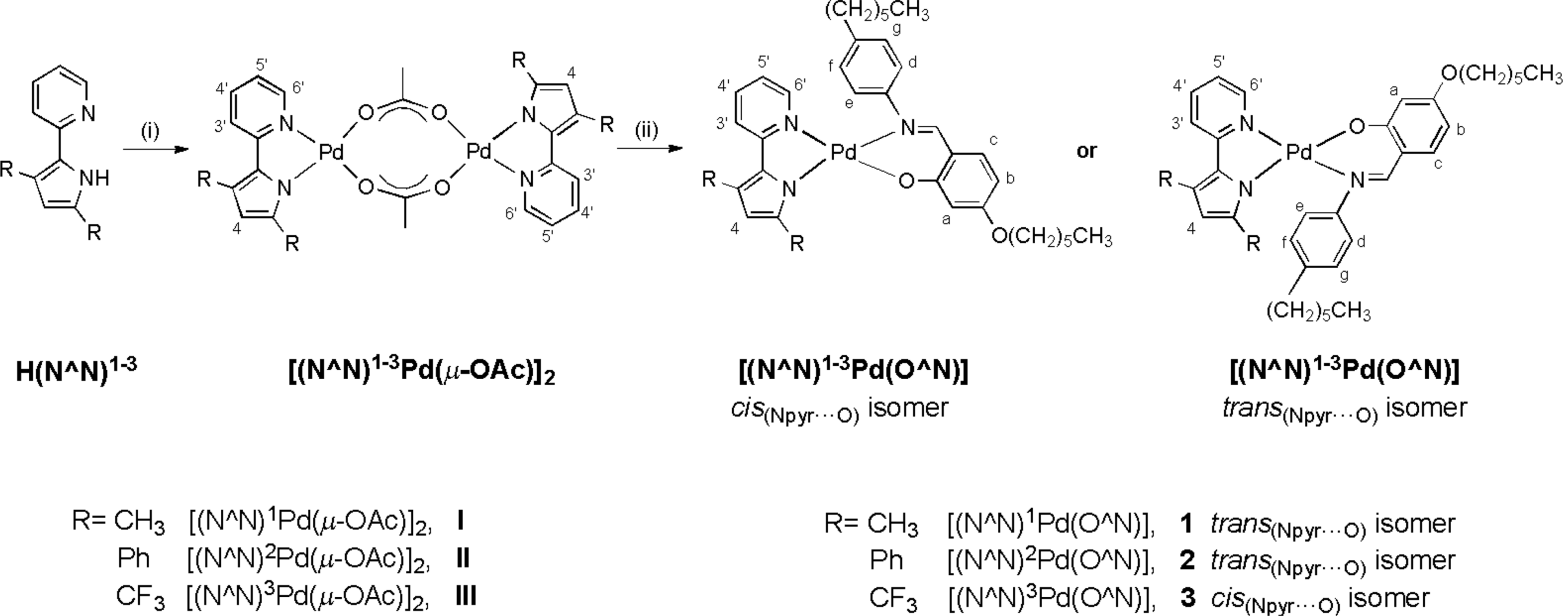
Synthetic Route to the [(N^N)^1–3^Pd(O^N)] Complexes **1**–**3** (i) Pd(II) acetate, dichloromethane, 25 °C, 2 h; (ii) H(O^N), ethanol, 25 °C, 6 h.

The synthetic strategy adopted for the preparation of the [(N^N)_1–3_Pd(O^N)] complexes (**1**–**3** in Scheme 1) is similar to the one typically used for the preparation of analogous [(C^N)Pd(O^N)] complexes,^20^ for which two reaction steps are needed. First, the formation of Pd(II) acetate-bridged intermediates (**I**–**III**) was obtained by reaction of the H(N^N)^1−3^ ligands with Pd(II) acetate. The bridge cleavage reaction of **I**–**III** was subsequently performed using the H(O^N) Schiff base, prepared as previously reported^20^ (Scheme 1).

The obtained thermally stable complexes **1**–**3** were fully characterized by microelemental analyses, ^1^H NMR, UV–vis spectroscopy, and single crystal X-ray diffraction. For these complexes, in principle, two isomers may be possible, depending on the relative position of the N^N/O^N chelated ligands (Scheme 1). However, all complexes **1**–**3** showed one set of ^1^H NMR signals, indicating the presence of only one isomer in solution. All ^1^H NMR spectra are reported in Figures S2–S7.

**Figure 1:**
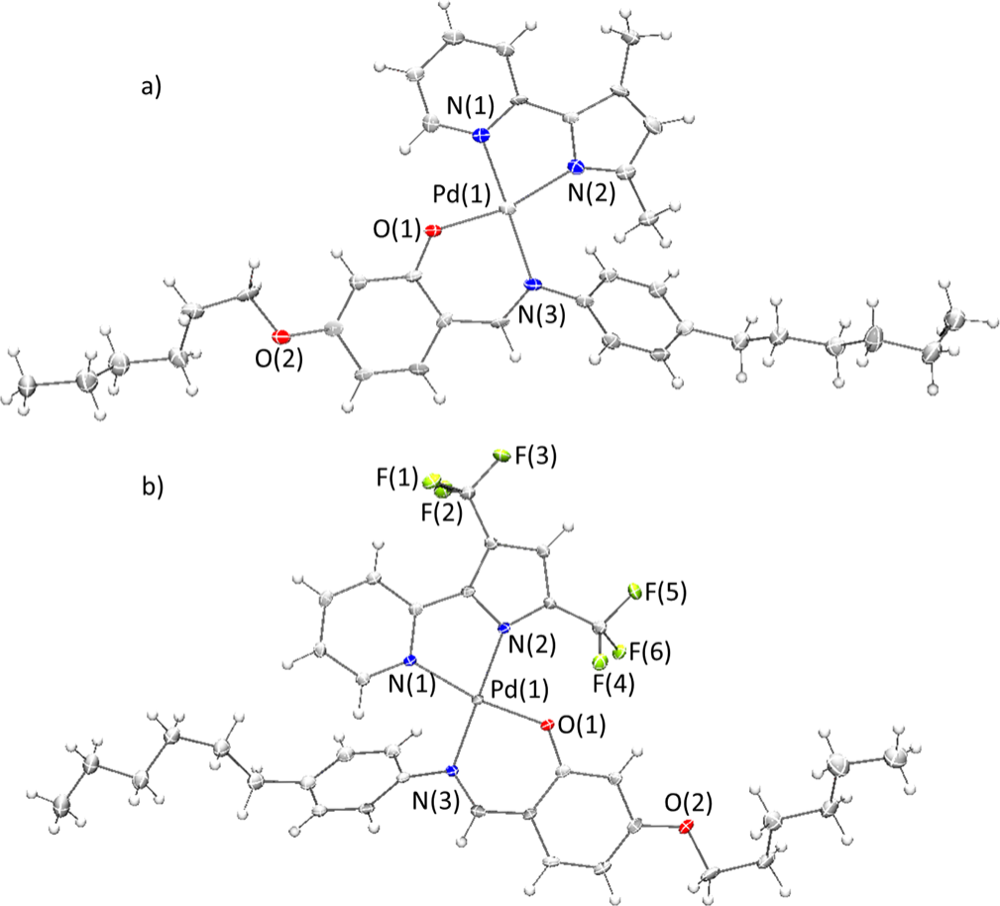
ORTEP view, drawn with atom displacement ellipsoids at a 40% probability level, of one of the molecules of the asymmetric unit of complexes **1** (a) and **3** (b). For clarity only selected atoms have been labeled.

Single-crystal X-ray data indicate that the asymmetric units of complexes **1** and **3** are made up of 2 and 3 molecules, respectively. Relevant bond distances and angles are reported in Table 2. ORTEP views of a single molecule of **1** and **3** are reported in Figure 1.

Complexes **1** and **3** are both characterized by the presence of the Pd(II) metal ion, in a slightly distorted square-planar geometry, at the center of two metallacycles: the N,O six-membered (N^O) and the N,N five-membered (N^N) rings, respectively, due to the chelation to the metal ion of the Schiff base ligand and of the 3′,5′-disubstituited-2,2′-pyridilpyrrole ligands H(N^N)^1,3^ (Table 2).

In agreement with the ^1^H NMR observation, in both cases, only one isomer was isolated in the crystalline solid state. Noteworthy, the PXRD patterns of the synthesized compounds are in agreement with the respective simulated PXRD patterns deriving from the single-crystal structures (Figures S8 and S9), confirming that only one isomer in both cases has been synthesized and then isolated. However, a single but different isomer was obtained for the two complexes, depending on the pyridylpyrrole ligand. In the case of complex **1**, the pyridine N(1) atom of the coordinated pyridylpyrrole ligand H(N^N)^1^ is found to be in *trans* position with respect to the bound nitrogen atom of the Schiff base ligand. The nitrogen donor atom of the pyrrolic ring (N_pyrr_), therefore, behaves as the carbon atom in similar 2-phenylpyridine cyclometalated derivatives, where the C,O *trans* isomer is found.^40^ In analogy, the Pd–O bond distances found in **1** confirm a less pronounced *trans* influence of the N_pyrr_ atom with respect to the cyclometalated carbon atom.

When the H(O^N) ligand coordinates the Pd(II) diperfluoromethyl pyridylpyrrole moiety, the isolated isomer found in the case of complex **3** is in an opposite configuration, with the N_pyrr_ donor atom in *cis* position with respect to the bound oxygen atom of the Schiff base ligand, that is, *cis*_(N_pyrr_···O)_ configuration. Indeed, the Pd–O bond distances are found slightly shorter than those found in complex **1**.

**Figure 2:**
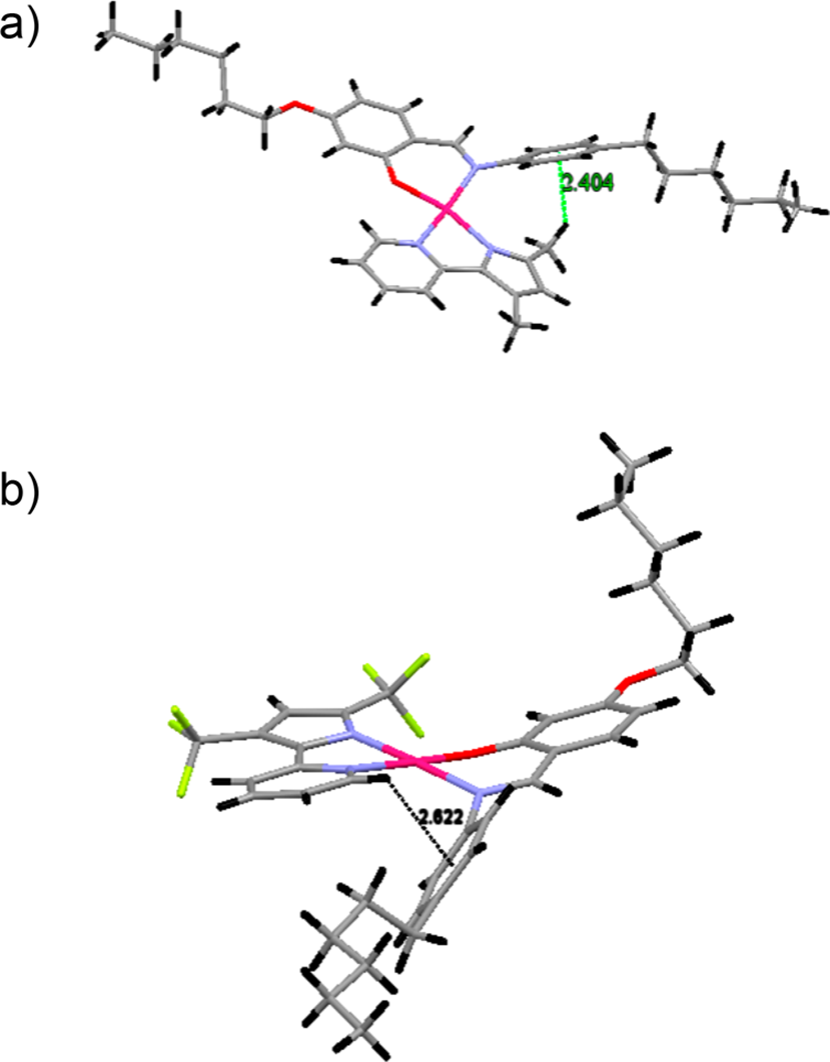
View of complexes **1** (a) and **3** (b) showing the CH−π intramolecular interactions [H–phenyl plane distances shorter than 2.5 Å.

Clearly, the substitution of the methyl groups with the fluorinated fragments causes noticeable electronic effects capable of directing the chelation of the ancillary ligand during the bridge splitting reaction, leading to the preferential and exclusive formation of the *cis*_(N_pyrr_···O)_ isomer with respect to the *trans*_(N_pyrr_···O)_ isomer previously obtained for the methyl substituted pyridylpyrrole (see discussions below about our computational DFT-based results). In any case, differences in intramolecular interactions derived from the crystal structure of complexes **1** and **3** are the most evident consequence of both the presence of fluorine atoms and the different isomers formed. CH−π attractive intramolecular interactions are established between the rotationally free phenyl ring of the Schiff base ligand and the hydrogen atom of the methyl group of the coordinated pyridylpyrrole ligand in **1** and the aromatic ring in **3** (Figure 2a).

Very short H–phenyl plane distances characterize these interactions, and all geometrical parameters are indicative of their presence.^41^ In complex **3**, the fluorine atoms of the CF_3_ groups seem to drive the crystal packing by establishing numerous C–H–F intermolecular interactions (Figure S10), being then considered both cause and effect of the formed preferential isomer.

If it has been possible to obtain crystals of good enough quality for single-crystal X-ray diffraction analysis for complexes **1** and **3**, all attempts to crystallize complex **2** were unsuccessful. However, the chemical shift variation of the ^1^H NMR signal of the proton hold by the carbon atom in α-position to the nitrogen atom of the pyridine ring (H_6′_, see Scheme 1) could be an indication of the nature of the isomer formed during the reaction.

Indeed, the H_6′_ chemical shift for the free ligands is almost unaffected by the nature of the substituents grafted onto the pyrrole ring, 8.45 ppm for H(N^N)^1^, 8.51 ppm for H(N^N)^2^, and 8.58 ppm for H(N^N)^3^. Note that the slight upfield variation in value across the series is consistent with the increase in electronegativity of the substituents going from Me, Ph, to CF_3_.

For complex **1** (*trans*_(N_pyrr_···O)_ isomer), the chemical shift registered for H_6′_ is 8.52 ppm, almost identical to the free ligand. Instead for complex **3** (*cis*_(N_pyrr_···O)_ isomer), a downfield effect of 1 ppm is observed, with the chemical shift of H_6′_ becoming 7.56 ppm. This effect must be therefore correlated to the *cis*_(N_pyrr_···O)_ configuration of complex **3**. For complex **2**, H_6′_ chemical shift is 8.58 ppm, similar to the free ligand, therefore pointing out the exclusive formation of the *trans*_(N_pyrr_···O)_ isomer.

DFT calculations were performed on **1**–**3** model complexes (see Computational Methods), obtained by replacing both the hexyl chains with shorter terminal methyl groups (see Computational Methods). Simulations showed that the relative stability of the two isomers is widely modulated by the substitution on the pyrrole moiety and, more interesting, by solvation effects. At the M06/SDD09/D95d level of theory (in vacuum), the *trans*_(N_pyrr_···O)_ isomer is always the more stable in energy (11.0, 31.0, and 8.9 kJ/mol in favor of *trans*_(N_pyrr_···O)_ for **1**–**3**, Table S1). The preference for this isomer is much higher in **2** than in **1** and **3**, and **3** shows the lowest preference. A detailed decomposition of the total bond energy between the central metal and the two ligands indicates that the main origin of the *trans*_(N_pyrr_···O)_ higher stability is the smaller electrostatic repulsion between the two chelants, which in turn can be traced back to the longer distance between the N^N N_pyrr_ and O^N O donor atoms (see SI for details). The particularly high stability observed in the **2***trans*_(N_pyrr_···O)_ isomer is due to the higher electrostatic and steric (Pauli) repulsions in the *cis*_(N_pyrr_···O)_ isomer (Tables S2 and S3 and related discussions in the SI). On the other hand, in the **3***cis*_(N_pyrr_···O)_ isomer, a smaller (respect to **2**) ligand–ligand repulsion and a relatively favorable metal–ligand interaction reduce its energy gap with respect to the *trans*_(N_pyrr_···O)_ isomer.

Structure optimizations in solvents of different dielectric properties (chloroform, dichloromethane, ethanol, and water) suggest that the solvent reaction field reduces the energy difference between the two isomers on increasing the dielectric properties of the solvent from dichloromethane to ethanol and water (see Figure S11 and related discussion in the SI). Whereas in ethanol, the **1** and **2***cis*_(N_pyrr_···O)_ isomers continue to be less stable by 4.7 and 20.3 kJ/mol, respectively, and the **3***cis*_(N_pyrr_···O)_ isomer becomes more stable by 1.0 kJ/mol. The effect of solvation can be associated with the larger dipole moments of the *cis*_(N_pyrr_···O)_ isomers (6.3, 7.3, and 11.1 D for **1**, **2**, and **3** in vacuum) in comparison to the *trans*_(N_pyrr_···O)_ ones (1.3, 2.0, and 8.9 D in vacuum, respectively). Hence, the computations suggest that the ethanol environment used for the synthesis and precipitation of all the complexes can favor the **3***cis*_(N_pyrr_···O)_ form. Moreover, in the solid phase, its larger dipole moment can be still more important in stabilizing the crystal lattice of this isomer, allowing the isolation of the **3***cis*_(N_pyrr_···O)_ form.

**Figure 3:**
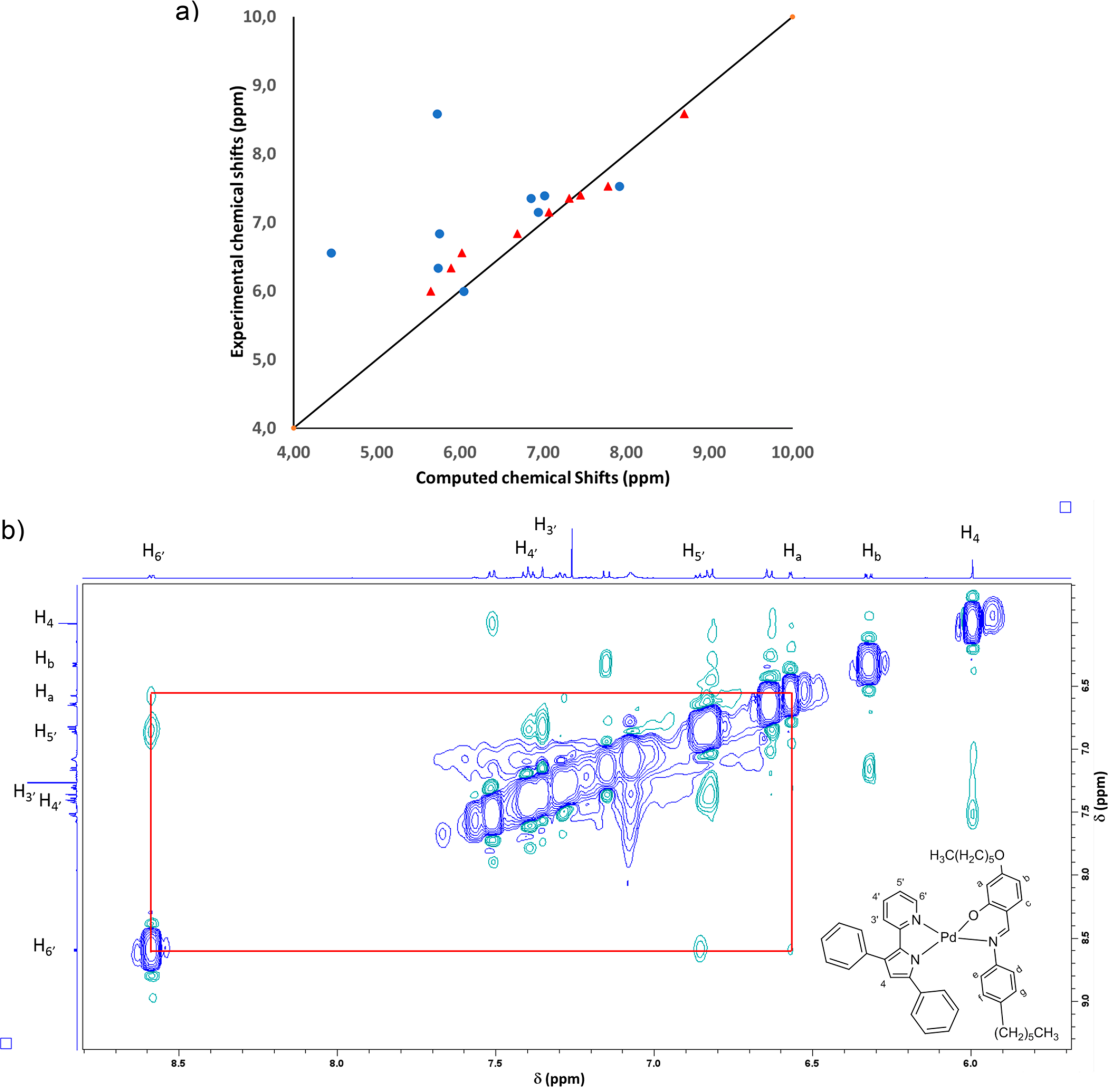
(a) Correlation between computed and experimental ^1^H NMR chemical shifts. Red triangle **2***trans*_(N_pyrr_···O)_ and blue circles **2***cis*_(N_pyrr_···O)_. (b) 2D-NOESY ^1^H NMR for **2**; the red line shows the correlation between H_6′_ and H_a_.

To remove any possible doubt of the exact configuration of complex **2**, energy information by more accurate DFT calculation at the M06/ECP28MDF:cc-augVQZPP/cc-pVQZ/CLF level of theory was performed and also confirms the *trans* as 22.5 kcal/mol more stable than *cis* isomer. We also computed the NMR chemical shifts of hydrogen atom nuclei of the three compounds at different levels of theory. Values computed at the M06/EM60DF:cc-aug-VQZPP/cc-pVQZ/CLF level of theory after optimization at the same level of theory in the case of compound **2** (Figure 3a) show a fairly good correlation with the experimental values only in the case of the *tran*s isomer. In the case of the *cis* isomer, hydrogen atoms nuclei (H_4_, H_4′_, H_a_, and H_b_) do not correlate at all with the experimental ones (Figure S13). Comparable results for isomers **1** and **3** (Figures S14 and S15) corroborate the quality of the results and the indication of the kind of isomers on the ground of DFT NMR chemical shift calculations. Further evidence comes also from the 2D-NOESY ^1^H NMR spectrum effectuated on a degassed (N_2_) CDCl_3_ solution. Indeed, a weak correlation has been observed between the signal of the proton placed in α-position of the pyridine ring of the (N^N)^2^ ligand (H_6′_) and the signal of the H_a_ proton of the ancillary Schiff base ligand (Figure 3b).

### Electrochemical Studies and Density Functional Theory Calculations

**Figure 4:**
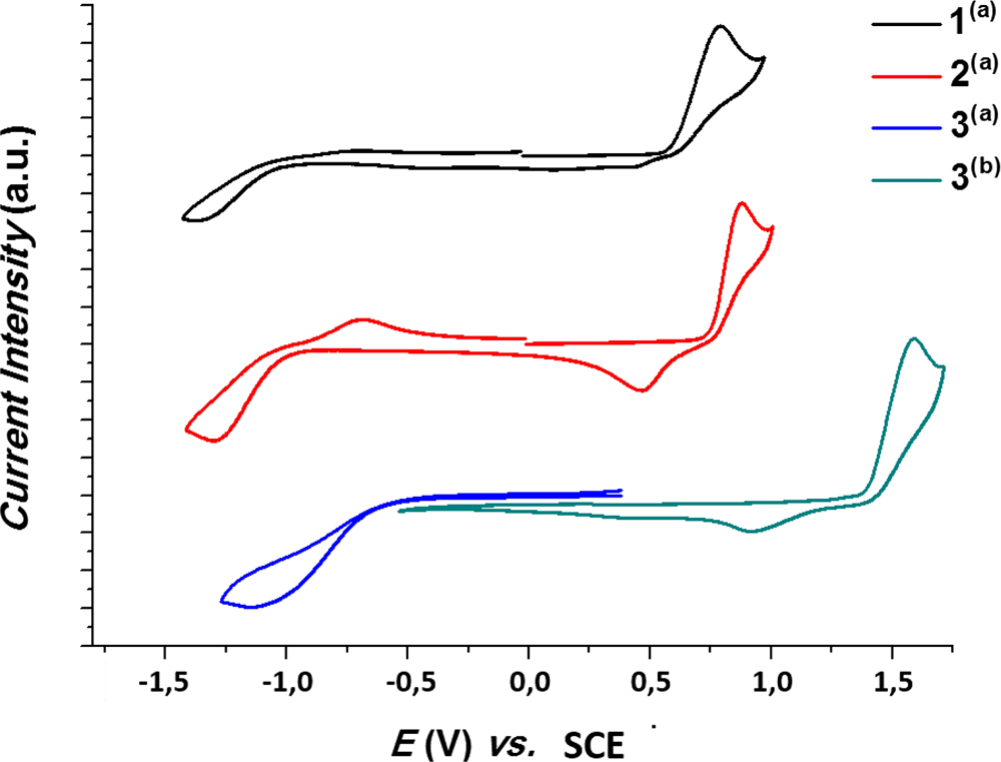
Cyclic voltammograms of complexes **1**–**3** in dry tetrahydrofuran (a) or dichloromethane (b). Traces are reported vs SCE that were plotted using Fc/Fc^+^ as the internal standard.

The redox properties of complexes **1**–**3** were investigated by cyclic voltammetry in dry and degassed (Ar) tetrahydrofuran or dichloromethane solutions, using ferrocene as internal reference. Complex **3** required the use of dichloromethane, since its oxidation potential fell outside the tetrahydrofuran potential window. Oxidation and reduction processes are irreversible for all complexes, although quasi-reversible processes could be attributed to complex **2**, as evidenced in the cyclic voltammograms illustrated in Figure 4 vs SCE, taking into account *E*_(Fc/Fc^+^)_^ox^ = 0.46 V vs SCE.^42^

Oxidation and reduction potentials vs Fc/Fc^+^ are reported in Table 3 together with the estimation of the corresponding HOMO/LUMO energy levels, evaluated by taking into account −4.8 eV for the HOMO energy level of ferrocene and by using the equation^43^*E* = (−1.4 ± 0.08) × *qV*_CV_ + (−4.6 ± 0.08), where *q* is the number of exchanged electrons and *V*_CV_ is the measured CV potential.

**Table 3:** Oxidation and Reduction Potentials of Complexes **1**–**3** (vs Fc/Fc^+^) and Corresponding Estimated HOMO/LUMO Energy Values

complex	*E*^ox^ (mV)^a^	*E*^red^ (mV)^a^	HOMO (eV)	HOMO^b^ (eV)	LUMO (eV)
**1**	+286 (Irr)	–1840 (Irr)	–5.09	–5.00	–2.96
**2**	+380 (Irr)	–1800 (Irr)	–5.18	–5.13	–3.00
**3**	+1090 (Irr)	–1601 (Irr)	–5.89	–6.13	–3.20

a All potentials are given vs Fc/Fc^+^. Irr: Irreversible wave.

b Values computed according to the equation *E* = −(1.4 ± 0.08) × *qV*_CV_ – (4.6 ± 0.08). *E*^ox^ = *E*^pa^ and *E*^red^ = *E*^pc^ for irreversible processes (*E*^pa^ = anodic peak potential; *E*^pc^ = cathodic peak potential).

The oxidation potential, *E*^ox^, increases within the series as follows: **1** < **2** ≪ **3**, with ca. 850 mV difference between the oxidation potential of the CH_3_ substituted complex **1** and that of the CF_3_ substituted analogue **3**. This observation seems to hint, deceptively, that the HOMO energy is highly influenced by the overall electronegativity of the (N^N) chelating ligand, suggesting its localization on this moiety. On the other hand, smaller changes (0.24 mV) in the reduction potential, *E*_red_, compared to the oxidation potentials are observed within the series **1**–**3**. This could suggest that the LUMO is more localized in the part of the molecule less sensitive to the pyrrole substitution. Note that for complex **2** (both in oxidation and reduction) and complex **3** (oxidation only), on their cyclic voltammograms, a reversed wave of smaller intensity is observed, most likely due to adsorption of the oxidized (respectively reduced) species onto the working electrode surface.

**Figure 5:**
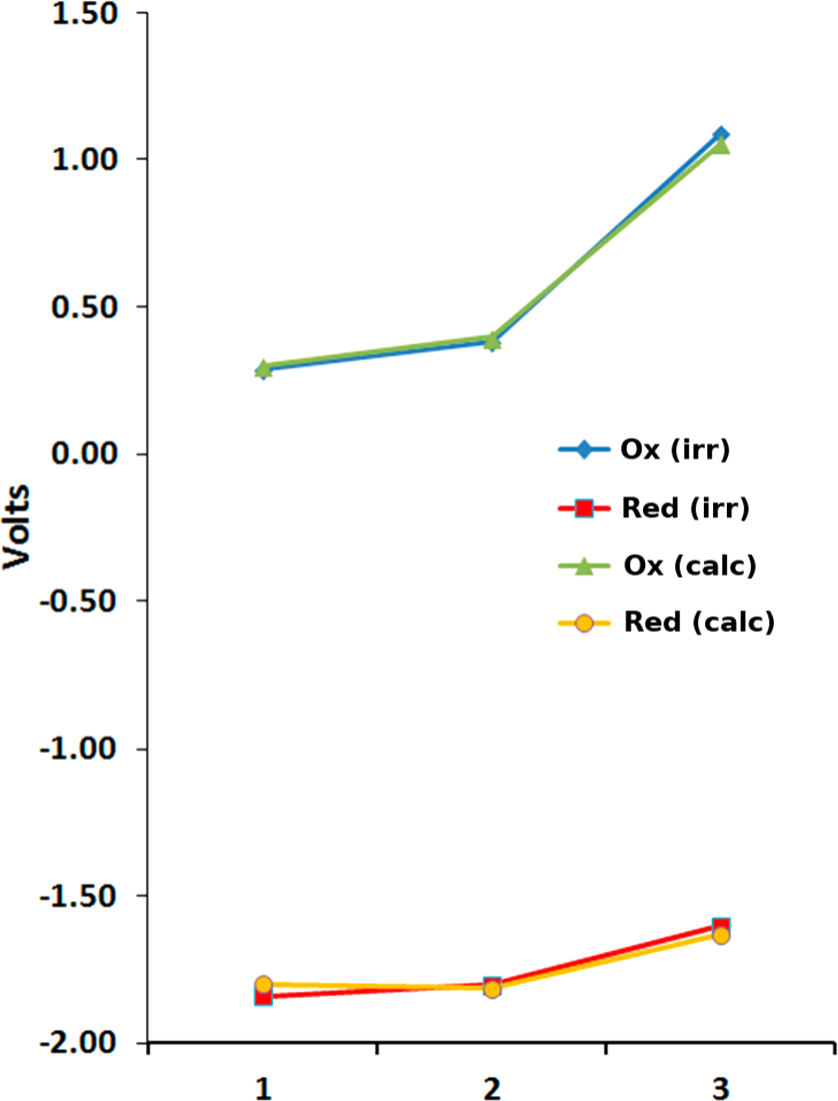
Comparison of the experimental and computed oxidation and reduction potentials for complexes **1**–**3**.

**Figure 6:**
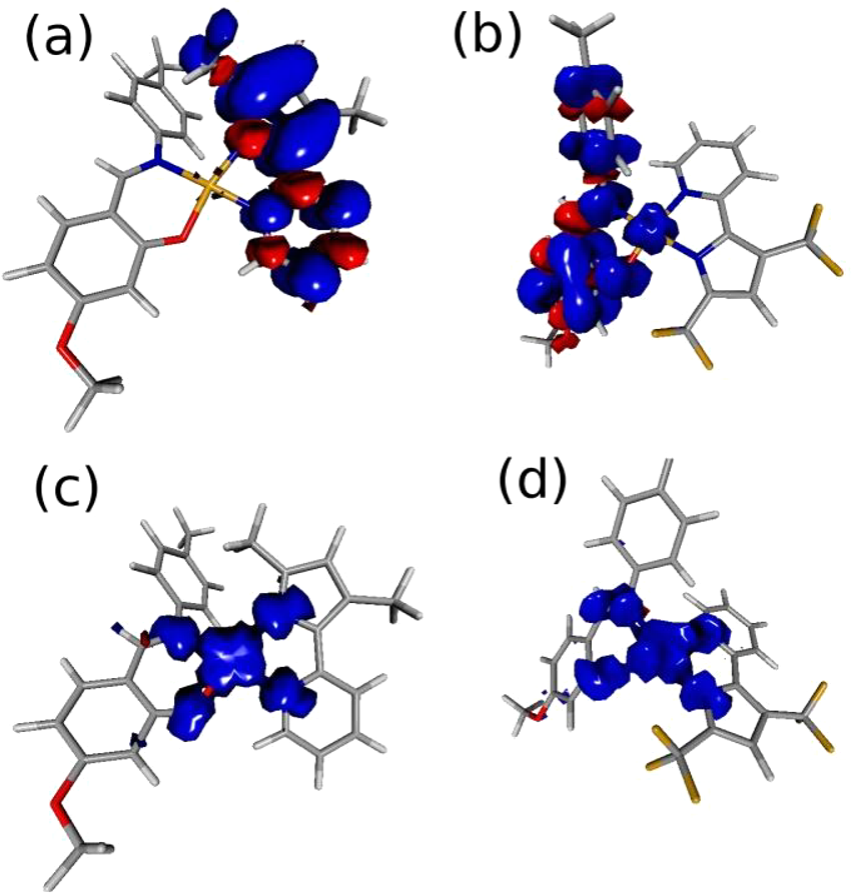
Computed spin densities of the cationic forms of **1** and **3** (a and b, respectively) and their anionic forms (c and d) at the mPW1PW91/SDD09/D95d/DCM level of approximation. Spin density surfaces are drawn at 0.002 e·bohr^–3^.

DFT computed oxidation and reduction potentials (on model **1**–**3** structures, see Computational Methods) at the mPW1PW91/D95(d)/SDD09/DCM level of theory (Figure 5) agree fairly well with the experimental ones. Furthermore, the computed spin densities of **1** and **2** cations confirm that, in both **1** and **2**, the positive charge is clearly localized on the (N^N) chelating ligand with a large contribution from the pyrrole ring (see Figure S16 and Figure 6a for **1**).

On the other hand, in case of **3**, the localization is reversed (Figure 6b), and the electron hole has the largest contribution from the O^N ligand. This evident change can be easily explained because of the large electron-withdrawing effect of the −CF_3_ substituents in **3**, with a consequent energy lowering of the N^N ligand fragment orbitals. This leads to the localization of the HOMO on the O^N ligand also in the complex. From Figure S17, in this respect, it is possible to notice the swap of energy order of the HOMO and HOMO–1 orbitals passing from **1** (and **2**) to **3** in case of the neutral species.

The **1** and **3** cations spin densities in Figure 6a,b strongly resemble the computed HOMO of the neutral complexes (Figures S13), suggesting that relaxation of the electron density (SCF process) after the electron extraction from the highest occupied orbital does not change the localization of the hole in a significant way. The same was observed in **2** (Figures S16 and S17).

A different situation was observed in case of the anions. The computed anion spin densities (Figure 6c,d and Figures S16) are very similar in all the studied complexes, being mostly localized on the central metal and on the nearest four coordinated atoms. The spin density spatial distributions are indeed very different from the spatial distribution of the computed LUMO’s of the neutral species (Figures S17). Indeed, they strongly resemble those of the anions α-HOMO (resulting from unrestricted computations), easily identified as a d_*x*^2^–*y*^2^_* orbital (Figure S16). In this respect, the reduction process mainly involves the coordinated Pd(II) metal. These findings are in line with the experimental analysis, indicating a substantial difference in the localization of the hole and electron in the charged molecules and a larger similarity in the localization of the negative charge.

#### UV–vis Absorption Spectroscopy and Time-Dependent Density Functional Theory Calculation

**Figure 7:**
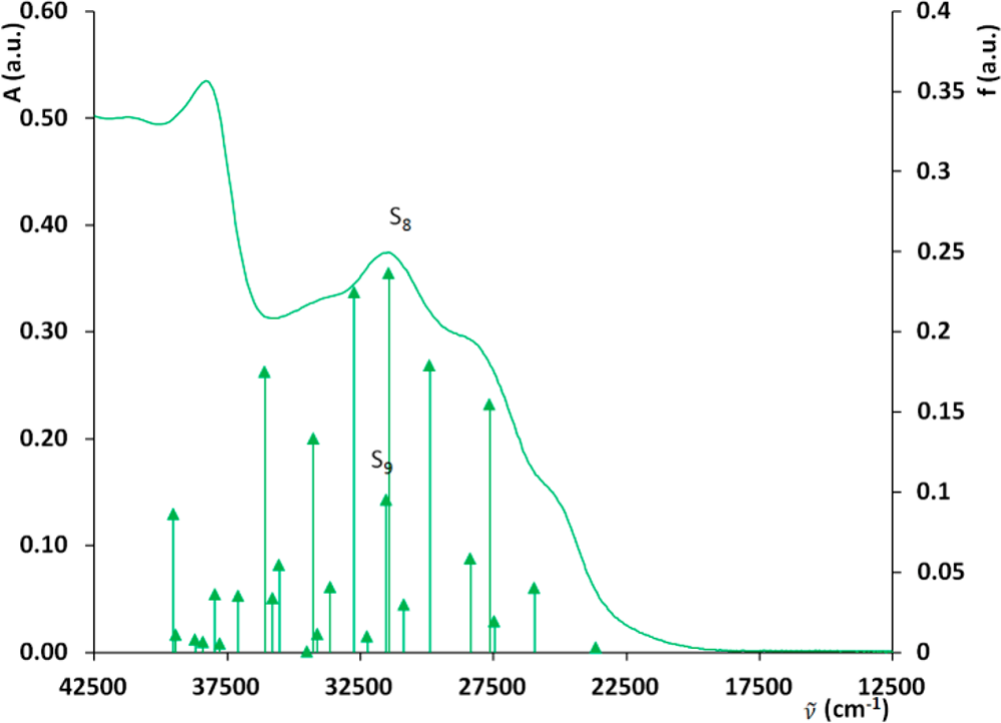
UV–vis absorption spectrum (solid line), oscillator strength, and position of the transitions computed by TD-DFT at the mPW1PW91/SDD09/D95d/DCM level of theory for complex **3** (vertical arrows).

The optical properties of complexes **1**–**3** were analyzed by UV–vis spectroscopy. The absorption spectra were recorded in dichloromethane solution (Figure S18), and the numerical data are reported in Table S4. In order to study the photophysical properties and the exact nature of the absorption bands of all complexes, theoretical approaches based on TD-DFT were performed. Absorption spectra (Figure 7) can be divided into five zones. All spectra show a long low-energy tail extending from 430 to 500 nm (23,256 to 20,000 cm^–1^). In the range of 380–430 nm (26,315–23,256 cm^–1^), **1** and **2** spectra show well-defined bands at 390 (25,641 cm^–1^) and 396 nm (25,253 cm^–1^), respectively, and in the case of **3**, this feature appears as a shoulder at about 395 nm (25,316 cm^–1^). In the 325–380 nm (30,769–26,316 cm^–1^) range, **1**–**3** show shoulders only at 343, 326, and 343 nm, respectively. Intense bands are present in the range 250–325 nm (40,000–30,769 cm^–1^), at 320 nm (31,250 cm^–1^) for **1**, 300 nm (33,333 cm^–1^) for **2**, and 318 nm (31,447 cm^–1^) for **3**. Finally, at 261 nm (38,314 cm^–1^), complex **3** spectrum shows an intense band, while **1** and **2** spectra display a shoulder at 265 (37,736 cm^–1^) and 260 nm (38,462 cm^–1^), respectively.

The TD-DFT was applied with the goals to assign the experimental features and also to check the accuracy of the chosen method (mPW1PW91/SDD09/D95d) before its application to the interpretation of the observed photoconductivity in the next paragraph. The detailed assignment of the experimental spectra is reported in Figure S19 and Table S5; here we only summarize the main results.

In **1**, spectral features are engendered by two clusters reflecting the overall experimental shape of the spectrum: The first one at lower energy and lower intensity and the second at higher energy and of higher oscillator strengths. In the case of **2**, even the lower wavelength group has a more intense transition than in case of **1**. Complex **3**, on the other hand, shows a set of comparable intensity transitions more evenly distributed in the 33,000–27,500 cm^–1^ range. These transitions lose the clustering behavior found in the cases of **1** and **2**, engendering less separated features. The overall spectrum of **2** shows a longer low-energy tail, whose displacement to higher wavelengths, compared to **1** and **3**, is a complementary effect of the low energy and higher oscillator strength of the S_2_, S_3_, and S_4_ transitions in the case of **2** and lower energy S_1_ in the case of **1**. In the case of **3**, S_2_, S_3_, S_4_, and S_5_ transitions move to higher energy and have a lower oscillator strength than in **2**, shifting the overall spectrum to higher energies.

All complexes were characterized to determine their luminescence properties. In dichloromethane solution at room temperature, no emissions were measurable. To verify if the complexes were emissive in a rigid matrix at 77 K, they were dissolved in a 5:5:2 mixture of cyclohexane, ethanol, and 2-methylbutane, which presents the same dielectric constant of dichloromethane but a lower scattering noise. A weak luminescence band, peaking at 473 nm, was observed for **1** only, with a lifetime of 3.70 ns, which accounts for a fluorescence emission (Figure S20).

With the aim to support this hypothesis, TD-DFT computations were performed (see Computational Methods for details and Table S12). After structural relaxation, the computed S_1_ is predicted to emit at 477 nm, in good agreement with the experiment, due to a metal-to-ligand charge-transfer exitation. Interestingly, the relaxed S_2_ is computed at only 5.3 kJ/mol from S_1_. Its metal-centered nature (a d_σ_*–d_π_* excitation) is expected to quench emission due to a strong intersystem crossing toward nonemissive triplet states.^44^ Its energy proximity to S_1_ explains the fact that only at 77 K such an emission can be recorded and also explains its low intensity. On the other hand, T_1_ is computed at 641 nm from the ground state, thus relatively far away from the observed emission, confirming that the **1** emission is fluorescence. The same computational study was performed on **2** and **3**. Interestingly, these complexes differ from **1**, where S_1_ is the metal-centered quenching d_π_*–d* state. In **2** and **3**, the metal-to-ligand charge transfer is computed relatively higher in energy (see Table S12). This allows to explain the lack of emission from these complexes also at 77 K solutions.

Spin–orbit effects have been computed for the lowest triplet states of **1**–**3**. Such a study was performed on the same T_1_ relaxed structure used for Table S12, and the related results are reported in Table S13 and discussed in the associated text in the SI, together with a detailed description of the methods used (perturbative spin–orbit coupling based on ZORA and TD-DFT). Here we summarize the fact that in all the complexes, the lowest triplet exited states are substantially not mixed with singlet states by spin–orbit perturbation. The direct consequence is the very low computed oscillator strengths associated with the electronic transition from the lowest triplet states and the ground state. This finding is in line with the lack of experimental phosphorescence in solid solutions. Moreover, such a low singlet–triplet spin–orbit coupling suggests that the studied complexes could present a long-living T_1_, even if not manifested by phosphorescence emission. The weak fluorescence observed in **1** as a possible result of thermal population of S_1_ from triplet states further corroborates this hypothesis. From above, the lack of phosphorescence could not be considered an evidence of a short T_1_ lifetime. This fact, combined with the nature of T_1_ in **1** (discussed in the following paragraph), can explain the high photogeneration observed particularly in this complex.

### Photoconductivity Studies

**Figure 8:**
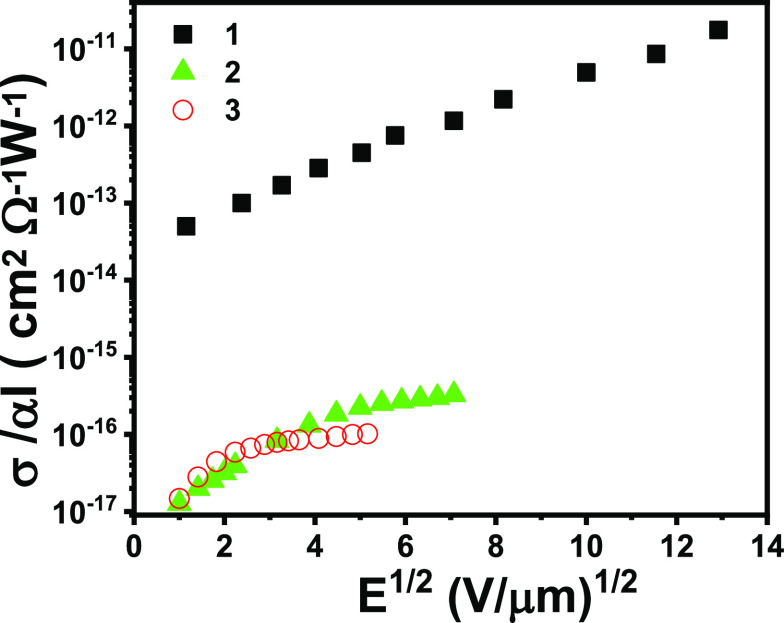
Normalized photoconductivity (σ) vs electric field intensities for complexes **1**–**3**.

The photoconductive properties of complexes **1**–**3** were studied on samples prepared according to the description reported in the Experimental Section. Figure 8 shows the photoconductivity of complexes **1**–**3** as a function of the applied electric field *E*. In all cases, photoconductivity was measured at wavelengths in the low-energy tail of absorption.

Photoconductivity depends on two factors: the amount of photogenerated charges and the existence of a relatively efficient mechanism for charge transport. The first factor can be described through a quantum efficiency of photogeneration, defined as the percentage of absorbed photons that yield a “free” charge, that is, a charge that can contribute to conduction. As the number of absorbed photons depends both on the intensity (*I*(λ)) of the incident radiation and on the light absorption coefficients (α(λ)), all data are presented in terms of the normalized photoconductivity σ_πη_/(*Ι*(λ)α(λ)). This allows a comparison among the different materials that is not affected by either their different absorption properties or by the different experimental conditions in terms of light intensity.

The photoconductivity of **1**, the best performing among all the studied complexes, is comparable to that of doped polyvinyl carbazole, a well-known photoconductor commonly used in optoelectronic applications.^45^ Note that polyvinyl carbazole requires sensitization with an electron acceptor, such as N-ethyl carbazole or fullerene, to increase photogeneration efficiency, while our measurements on **1**–**3** were carried out on undoped samples.

Furthermore, the photoconductivity of complex **1** has been compared with those of the previously studied photoconductive cyclopalladated complexes in order to evaluate the role of ligand substitution, and results are reported in Table 4.

**Table 4:** Comparison of Normalized Photoconductivity in the Complex **1** and in Different (C^N)Pd(O^N) Complexes

complex	σ_ph_/*I*α (cm^2^ Ω^–1^ W^1–^)^a^	ref
**1**	3 × 10^–13^	–
(PhPy)Pd(O^N)	5 × 10^–16^	(20)
(BzQ)Pd(O^N)	2 × 10^–15^	(20)
(PhPy)Pd(O^N)^tpa1^	2 × 10^–15^	(17)
(PhPy)Pd(O^N)^tpa2^	1 × 10^–14^	(17)
(PhPy)Pd(O^N)^tpa3^	2 × 10^–15^	(17)
(NR)Pd(O^N)^po^	4 × 10^–12^	(11)

a Measured at 20 V/μm. H(O^N)^tpa*i*^: (*i* = 1, 2 or 3) diversely functionalized Schiff Bases with a triphenylamine fragment (see ref (17)). H(O^N)^po^: Polyalkylated Schiff base (see ref (11)).

In a general overview, the photoconductivity of **1** is always higher than the photoconductivity reported for complexes of the (C^N)_*n*_Pd(O^N) class,^17,20^ with the only exception (ref (11)) related to a metallomesogen Pd(II) complex able to self-assemble into a columnar mesophase, a molecular organization known to boost charge mobility by several orders of magnitude.^46^

In an analogy with the observations in different complexes of the same class,^11,20^ it can also be assumed that the mobility of charges for these complexes is much larger for holes than for electrons. A direct measurement of the charge mobility of **1**–**3** would be essential for the understanding of the photoconductive behavior of these complexes. Several attempts to measure the hole mobilities of complexes **1**–**3** were carried out by using the space-charge-limited current method. However, in all cases the data did not allow the determination of mobility, as the SCLC regime was never achieved, even for high applied voltages. The measured currents were always low and linearly dependent on the applied voltages, even if the work function of the injecting electrode that was used (Au) matches the HOMO energy of the semiconductors, at least in the case of **1** and **2**.

**Figure 9:**
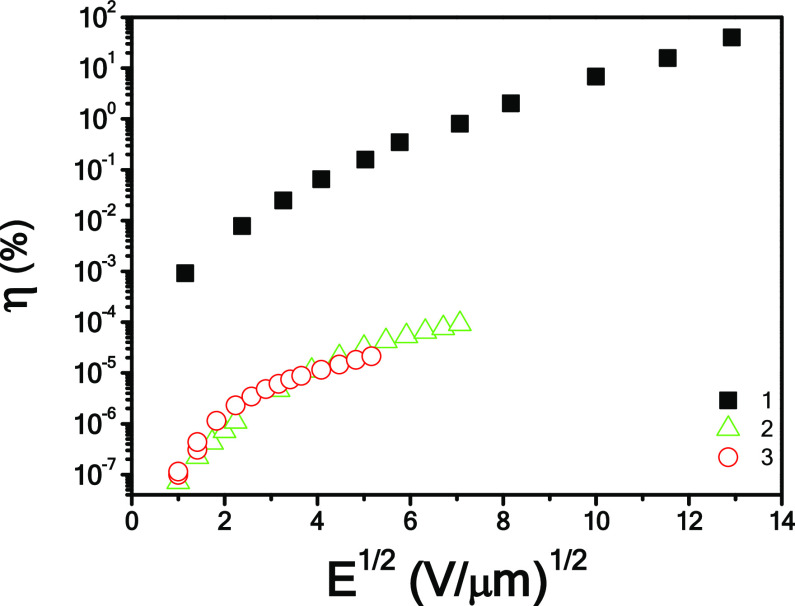
Photogeneration efficiencies of **1**–**3** as a function of the applied electric field, calculated according to eq 1.

In this light, in order to discriminate between charge mobility and photogeneration efficiency as the crucial factor explaining better photoconductivity of **1**, we determined the photogeneration efficiency η (*E*) of **1**–**3**. Photogeneration efficiency can be estimated from photoconductivity as^47^
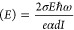
1where ℏω is the photon energy, *e* is the elementary charge, and *d* is the thickness of the sample. The applied field dependence of the photogeneration efficiencies for **1**–**3** are shown in Figure 9.

The estimated η for **1** is about 4 orders of magnitude higher than for **2** and **3**, approaching complete conversion of radiation energy into mobile charges at very high applied fields.

Photoconduction data were obtained from samples which were either amorphous or polycrystalline, with domains (grains) of different orientation. However, the molecular organization of **1** and **3** in such samples can be safely assumed as being, at least in the short-range, the one deduced from SCXRD studies. Furthermore, population of triplet states appeared to be important in the photoconduction studies performed on similar complexes.^20a^ On the basis of these two considerations, a computational analysis was carried out in order to gather further evidence in favor of the better photogeneration efficiency of **1** with respect to those of **2** and **3**.

**Figure 10:**
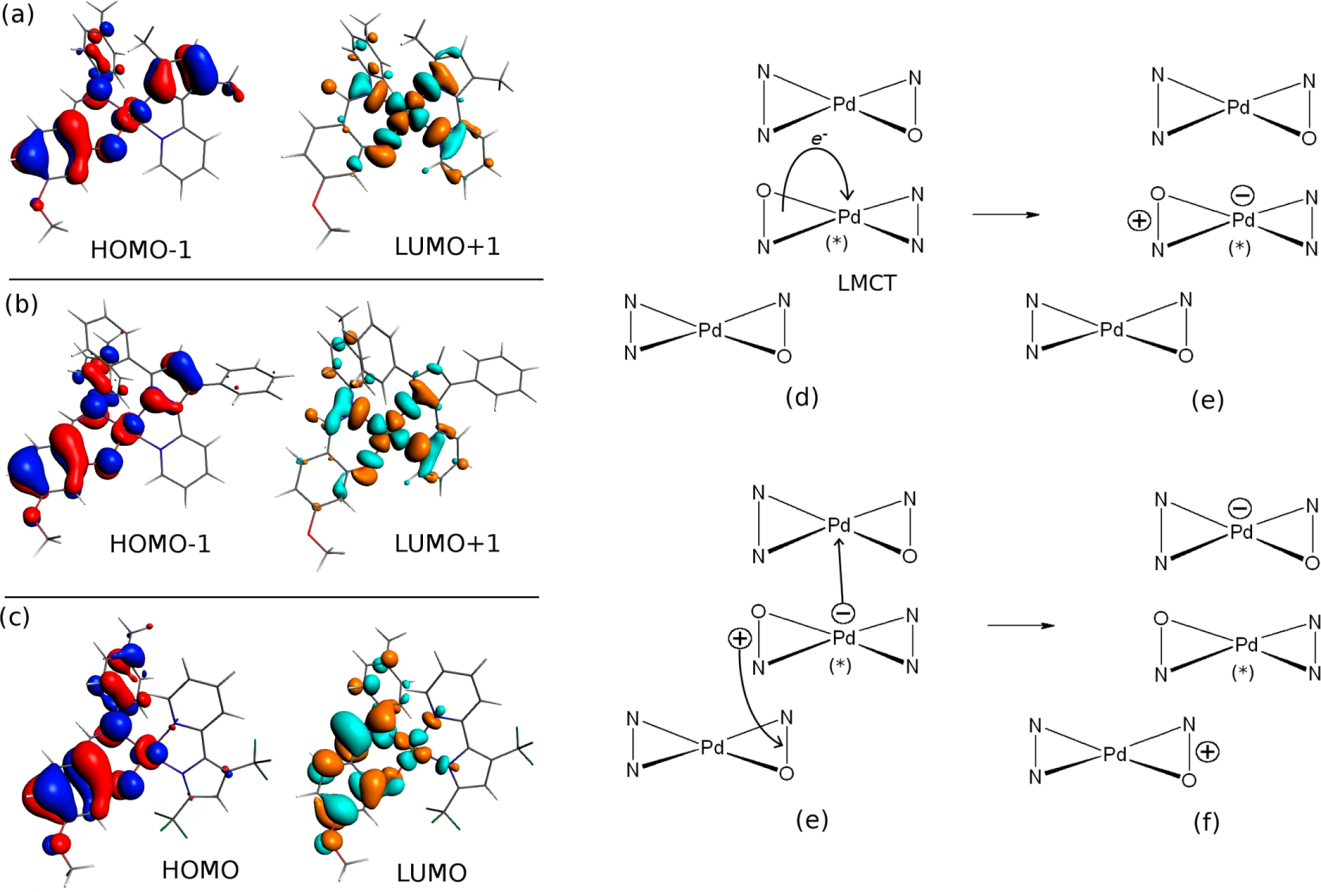
(a–c) Molecular orbitals computed on the T_1_ relaxed structure of **1** (a), **2** (b), and **3** (c) (MPW1PW91/ZORA/DZP from the ADF computations) which are relevant in describing the T_1_ excited state. The scheme on the right describes the proposed mechanism explaining the photogeneration efficiency in **1**. (d–f) Schematic view of the infinite stack found in the **1** crystal (see Figure S21a). The two molecules on the top and bottom of the one with the asterisk give a simplified view of the contacts present in the infinite stack. From (d, e), the metal-to-ligand charge transfer (producing T_1_) induces an intramolecular charge separation, with the hole mainly on the O^N ligand and the electron on a d_*x*^2^–*y*^2^_* orbital (in (a), from HOMO–1 to LUMO). From (e, f), the electron and/or hole hopping toward adjacent molecules takes advantage of the favorable stacking with the molecules, respectively, on the top and bottom.

The photogeneration and photoconduction processes are strictly connected to the way in which adjacent molecules reciprocally arrange in the solid phase. As shown in Figure S22a and extensively discussed in the relative paragraph in the SI, the **1** crystal features an infinite stack of molecules in which each complex “overlaps” the adjacent one with their central metals and coordinated donor atoms in one direction and with their O^N ligands in the opposite direction. Figure 10d reports a schematic representation of the stack, stressing the two different contacts in the two directions (toward the top and the bottom of the molecule with the asterisk, taken as reference). Such a stack appears favorable to photogeneration and photoconduction in general, as discussed below. An infinite stack with similar overlaps can be observed in the **3** crystal as well. The **3** stack is equally discussed in Figure S22b.

The importance of triplet states in determining photoconduction^20a^ makes desirable a detailed description of the lowest in energy states of this spin multiplicity. Computational structure optimizations performed in vacuo on the singlet ground state showed a slight tendency of **1** and **3** to distort from the almost planar arrangement observed in the crystal structure to a pseudotetrahedral coordination geometry. This behavior, though negligible in the ground state, becomes strongly evident in the excited states, especially in T_1_ (see SI for a detailed report). The same computational study was performed in a solid-state model designed for the **1** crystal (see Figure S21 and related discussion in SI).

From this study, it appears rather unlikely that the molecular packing could allow the distortion observed in the case of the in vacuo excited-state calculation. In more detail, all the dihedral angles remain substantially unchanged from the values they assume in the experimental crystal. As the description of photogeneration processes in the solid phase is our major target, the excited-state structures (T_1_ and T_2_) were computed at the TD-DFT level after freezing all of the dihedral angles to the values of the experimental structure in the crystal. This thwarted possible changes in the electronic structure caused by structural changes of the ligand and the coordination sphere, which can more easily happen in vacuo.

The T_1_ structure relaxed triplet state is more stable than the T_2_ by 0.168 eV (16.2 kJ/mol). Thus, T_1_ should be definitely more populated than T_2_. The former is mainly associated with the monoelectronic excitation from the HOMO–1 to LUMO+1 (61% in weight). From Figure 9a, this excitation shows a clear character of charge transfer from the ligands to the d_*x*^2^–*y*^2^_* orbital of the metal. Thus, a hole–electron separation is expected to take place during the nonradiative relaxation toward T_1_, internally to a single molecule, possibly favoring photogeneration.

More interestingly, from Figure 9d and Figure S18, the stack in the **1** crystal seems suitable to further the electron–hole separation, from the stage of a hole–electron couple inside the excited molecule to the stage of a hole and an electron on adjacent molecules in the stack. In fact, one couple of molecules in Figure 10d (and Figure S22a) seems to allow easy electron hopping from an excited molecule (that is, d_*x*^2^–*y*^2^_* populated in T_1_) to the adjacent molecule (empty d_*x*^2^–*y*^2^_* orbital in the ground state, the process from (d) to (e) in Figure 10). At the same time, the second couple (Figure 10 from (e) to (f) and Figure S22a) seems suitable to hole hopping from the excited molecule (HOMO–1 depletion on the O^N ligand) to the adjacent one (not-depleted O^N ligand). Whichever transfer takes place (hole, electron, or both), the stack in **1** crystal seems to be compatible with a good photogeneration from T_1_.

The same study was performed on **3**. T_1_ and T_2_ were optimized after freezing all the dihedral angles at their experimental values in the crystal. Similarly to **1**, the T_2_ is predicted as barely populated at ambient temperature, being less stable by 20.5 kJ/mol compared to T_1_. However, T_1_ of **3** mainly originates from an intraligand excitation (on the O^N ligand). As in case of **1**, the transition consists in the HOMO–LUMO monoelectronic excitation (Figure 10c) contributing by 78%. Thus, no significant hole–electron segregation takes place. Therefore, it is possible to tentatively suggest that the worse photoconduction of **3** can be justified with the lower degree of hole–electron separation in its T_1_.

Missing the SCXRD structure of complex **2**, we used the ground-state in vacuo optimized structure as reference structure as the starting point for the structure optimization in T_1_ after freezing all the dihedral angles. The computed T_1_ excited state consists of a ligand to metal charge transfer (HOMO–1 to LUMO+1 in Figure 10b) with a percentage of 49%. The nature of this state is very similar to the one observed in **1**. Hence, the nature of T_1_ does not seem to be a possible explanation of its lower photoconduction. Here, we can only underline that one phenyl group on the N^N ligand is in the proximity of the central metal (and its donor atoms), hence, possibly, it can thwart the interaction of the d_*x*^2^–*y*^2^_* orbital between adjacent molecules.

## Conclusions

In this study we report the synthesis and photoconduction properties of new heteroleptic Pd(II) complexes with various 3′,5′-disubstituted-2-(2′-pyridil)pyrrole ligands, H(N^N)^1–3^ and a Schiff base H(O^N) as the ancillary ligand. In each case, a sole isomer is obtained, depending on the substituents present onto the pyridilpyrrole ligand (*trans*_(N_pyrr_···O)_ isomers for **1** and **2** and *cis*_(N_pyrr_···O)_ isomer for **3**).

Noteworthy, while complexes with the Ph (**2**) and CF_3_ (**3**) substituted ligands show photoconductive performances similar to those of the previously studied complexes, the CH_3_ derivative (**1**) induces a much higher photoconductivity. The experimental trend of photoconduction vs the applied electric field suggests that efficient photogeneration is the main reason for the observed excellent photoconduction.

The higher photoconduction of **1** can be traced back to the physical separation of the hole and electron already at the level of the excited molecule generated in the solid phase during irradiation. In fact, the behavior of **1** resembles that of the previously studied organometallic complexes (see Chart 1) and confirms the necessity to design coordination complexes with this important feature. Complex **3** does not show this separation, suggesting a hole–electron recombination as the cause of the observed lower photoconduction.

In conclusion, the excellent performances of **1** as a photoconductive material and its interpretation discussed in this paper open the route to a new class of squared-planar complexes which, compared to the more thoroughly studied organometallic analogues, can represent a further step forward. Further experimental studies are necessary to confirm our hypothesis about the origin of such a promising photoconductive behavior, which can be summarized in the following points: (1) Presence of charge-transfer ligand-to-metal triplet excited states with involvement of the d_*x*^2^–*y*^2^_* orbital and (2) molecular stacks with efficient interactions of the central metal and its donor atoms between adjacent molecules.

The synthesis of new complexes and their characterization are currently in progress in our laboratories to further support the importance of the central metal involvement in the processes underlying photoconduction.

## Supporting Information Available

Computational details, absorption spectra, optimized structures, photoconductivity details, H^1^–NMR spectra, correlation between computed and experimental chemical shifts (PDF)

## Accession Codes

CCDC 1959652 and 1959674 contain the supplementary crystallographic data for this paper. These data can be obtained free of charge via www.ccdc.cam.ac.uk/data_request/cif, or by emailing data_request@ccdc.cam.ac.uk, or by contacting The Cambridge Crystallographic Data Centre, 12 Union Road, Cambridge CB2 1EZ, UK; fax: +44 1223 336033.
