# Supplementary material for: Photoconductive Properties and Electronic Structure in 3,5-Disubstituted 2-(2′-Pyridyl)Pyrroles Coordinated to a Pd(II) Salicylideneiminate Synthon
Source: Inorg Chem. 2021 Jun 14;60(13):9287–301. doi: 10.1021/acs.inorgchem.0c02991 (PMC8277164; doi:10.1021/acs.inorgchem.0c02991)
Supplement: Supplementary file 1 — ic0c02991_si_001.pdf [file ic0c02991_si_001.pdf]

SUPPORTING INFORMATION

Photoconductive properties and electronic structure in 3,5-disubstituted 2-(2'-pyridyl)pyrroles coordinated to a Pd(II) salicylideneiminate synthon

Andreea Ionescu,[†,‡] Nicolas Godbert,[†] Roberto Termine,[‡] Massimo La Deda,[†,‡] Mario Amati,[§] Francesco Lelj,[§] Alessandra Crispini,[†] Attilio Golemme,[±,‡] Mauro Ghedini,[†,‡] Pilar Garcia-Orduña,[#] Iolinda Aiello[*†,‡]*

[†] MAT-INLAB (Laboratorio di Materiali Molecolari Inorganici) and LASCAMM - CR INSTM, Unità INSTM della Calabria, Dipartimento di Chimica e Tecnologie Chimiche, Università della Calabria, 87036 Arcavacata di Rende (CS), Italy

[‡] CNR NANOTEC-Istituto di Nanotecnologia U.O.S. Cosenza, 87036 Arcavacata di Rende (CS), Italy

[§] Dipartimento di Scienze and LASCAMM-CR-INSTM, Unità INSTM della Basilicata, Università della Basilicata, 85100 Potenza, Italy

[±] Dipartimento di Fisica, Università della Calabria, 87036 Arcavacata di Rende (CS), Italy

[#] Instituto de Síntesis Química y Catálisis Homogénea, Universidad de Zaragoza-CSIC. Pza. San Francisco s/n. Zaragoza, Spain

Contents:

Figure S1. Current versus time measured in a sample of **2**

Figure S2. ^{1}HNMR spectrum of **H(N^N)^1** in $CDCl_3$

Figure S3. ^{1}HNMR spectrum of **H(N^N)^2** in $CDCl_3$

Figure S4. ^{1}HNMR spectrum of **H(N^N)^3** in $CDCl_3$

Figure S5. ^{1}HNMR spectrum of **1** in $CDCl_3$: full spectrum (a), aromatic region with indexation (b)

Figure S6. ^{1}HNMR spectrum of **2** in $CDCl_3$: full spectrum (a), aromatic region with indexation (b)

Figure S7. ^{1}HNMR spectrum of **3** in $CDCl_3$ full spectrum (a), aromatic region with indexation (b)

Figure S8. PXRD profiles of **1**

Figure S9. PXRD profiles of **3**

Figure S10. Crystal packing view of **3** showing the predominant C-H---F interaction [C---F^i 3.67(1) Å, C-H---F 165°, *i* = -x+2,-y+1,-z]

Table S1. Relative energies of the **1**, **2** and **3** $cis_{(Npyrr...O)}$ and $trans_{(Npyrr...O)}$ isomers (kJ/mol) in vacuum and different solvents

Figure S11. Energy of the $cis_{(Npyrr\cdots O)}$ form compared to the $trans_{(Npyrr\cdots O)}$ form

Table S2. Decomposition of the ligand-metal energy bond in **1**, **2** and **3** $cis_{(Npyrr...O)}$ and $trans_{(Npyrr...O)}$ isomers

Table S3. Decomposition of the interaction energy (energy term) associated to Step 3 only. Energies are kJ/mol

Figure S12. SCF potential plots of the $cis_{(Npyrr...O)}$ and $trans_{(Npyrr...O)}$ isomers of **1**

Figure S13. Correlation between computed and experimental chemical shifts for the most relevant hydrogen atoms in case of **2** for cis and trans isomers

Figure S14. Correlation between computed and experimental chemical shifts for the most relevant hydrogen atoms in case of **1** for cis and trans isomers

Figure S15. Correlation between computed and experimental chemical shifts for the most relevant hydrogen atoms in case of **3** for cis and trans isomers

Figure S16. Computed spin densities of **1**, **2** and **3** cations and anions

Figure S17. Some of the MO's for **1**, **2** and **3**

Figure S18. Electronic absorption spectra of **1**-**3**

Table S4. Absorption data of **1**-**3** recorded in dichloromethane solution

Figure S19. UV-Vis spectra in dichloromethane of **1**-**3** and the computed transitions by TD-DFT at the mPW1PW91/SDD09/D95d/DCM level of theory

Table S5. Computed singlet excited states for **1**-**3**(MPW1PW91/SDD09/D95(d)/DCM)

Table S6. Computed Singlet excited states for **1**-**3** (M06/SDD09/D95(d)/DCM)

Table S7. Computed Cartesian coordinates (MPW1PW91/SDD09/D95(d)/THF) of neutral and charged complexes

Figure S20. Emission spectrum of **1** at 77 K

Figure S21. Graphical view of the structure optimizations performed with ONIOM

Table S8. Computed Cartesian coordinates of the **1**molecular cluster with ONIOM (singlet and triplet ground states)

Table S9. Test on the reliability of PM6 computations: single molecule *in vacuo*

Table S10. Test on the reliability of PM6 computations: **1** couple a-b

Table S11. Cartesian coordinates of the T_1 excited states of **1**-**3**

Figure S22. Views of the **1** and **3** infinite stacks in the crystals

Table S12. Computed singlet and triplet excited states of **1**-**3**.

Table S13. Excited states of **1**-**3** computed with inclusion of spin-orbit effects.

Photoconductivity measurements details

Photoconductivity measurements were carried out by applying on the sample a DC voltage and measuring the current in the dark and under illumination, as detailed in the following. A DC voltage is applied in the dark and the current flowing through the sample is measured. Once the current becomes stable, the light is turned on and the increase of the current is observed. When the current is again stabilized, the light is turned off. The difference between the value of the current measured in the dark and the one measured with the light on is called photocurrent i_{ph}. From i_{ph}, applying Ohm's, it possible to calculate the photoconductivity σ_{ph}:

$$\sigma_{ph} = \frac{i_{ph} d}{VA}$$

where d is the sample thickness, A is the sample area and V is the applied voltage. The current was measured with a Keithley 6517A electrometer, while the light was provided by a He-Ne laser at 633 nm for (compound **2**) or by a solid-state laser at 532 nm (for compound **3**) or at 600 nm by using a lamp/monochromator system (for compound **1**). The light was turned on/off by a Thorlabs Optical Shutter. Figure SI6 shows typical data of current versus time obtained in a sample of complex **2**.

Figure S1. Current versus time measured in a sample of **2.**

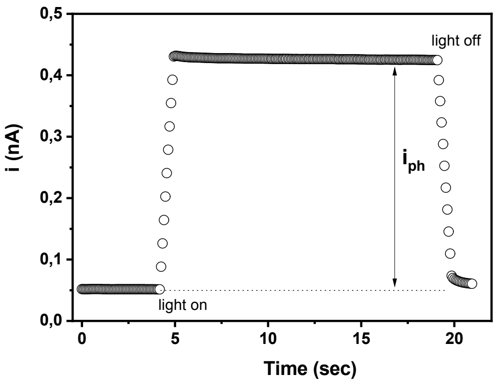

Figure S2. ^{1}HNMR spectrum of **H(N^N)^1** in $CDCl_3$.

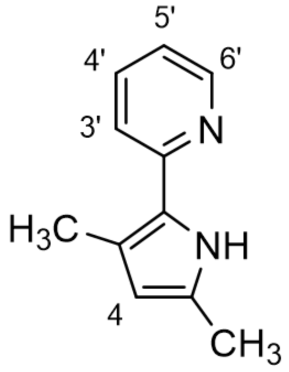

H(N^N)[1]

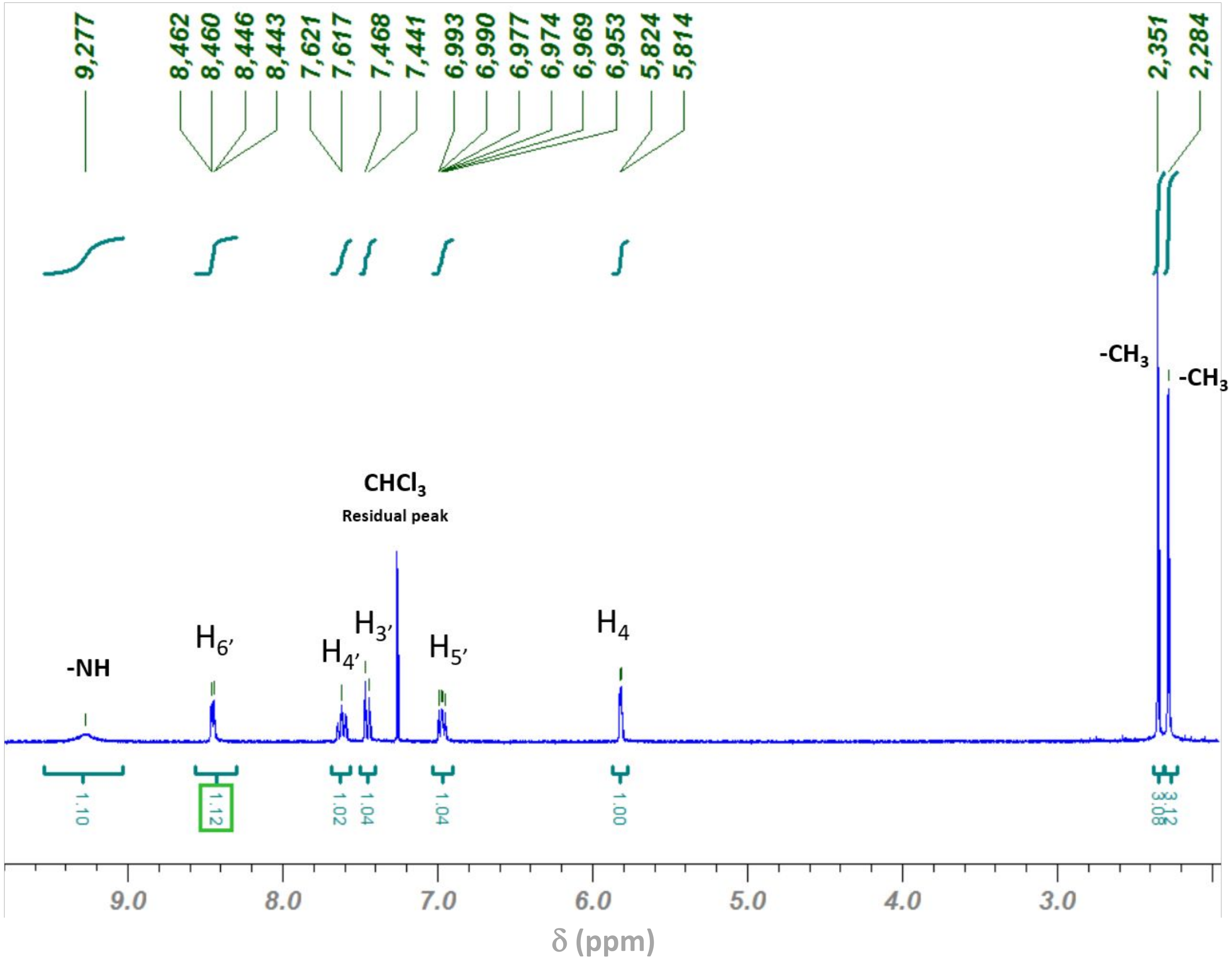

H(N^N)[1]: ^{1}H-NMR ($CDCl_3$, 300 MHz), δ (ppm): 9.28 (1H, broad s, NH), 8.45 (1H, dd, J = 4.8 Hz, J = 0.6 Hz, $H_{6'}$), 7.6 (1H, ddd, J= 7.2 Hz, J = 6.8 Hz, J = 0.6 Hz, $H_{4'}$), 7.45 (1H, dd, J = 6 Hz, J = 0.6 Hz, $H_{3'}$), 6.97 (1H, ddd, J=7.5 Hz, J=4.8 Hz, J=0.6 Hz, $H_{5'}$), 5.82 (1H, s, H_4), 2.35 (3H, s, CH_3), 2.28 (3H, s, CH_3).

Figure S3. ^{1}HNMR spectrum of **H(N^N)^{2}** in $CDCl_3$.

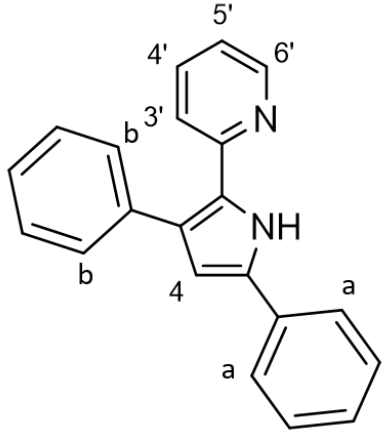

H(N^N)^{2}

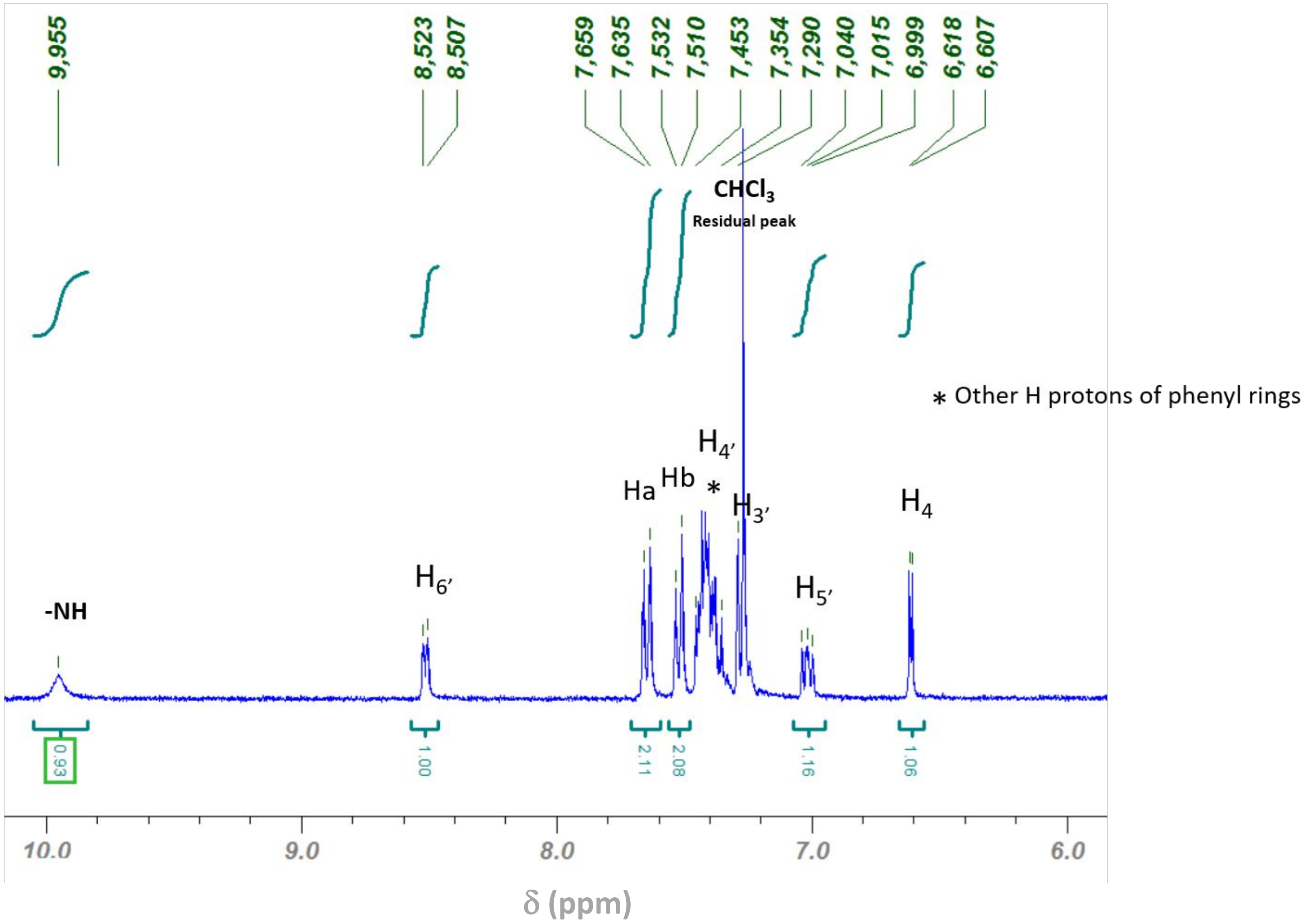

H(N^N)^{2}: ^{1}H-NMR ($CDCl_3$, 300 MHz), δ (ppm): 9.96 (1H, broad s, NH), 8.51 (1H, dd, J = 4.8 Hz, J = 0.6 Hz, $H_{6'}$), 7.6 (2H, dd, J= 7.2 Hz, J = 0.6 Hz, H_a), 7.52 (2H, dd, J= 7.2 Hz, J = 0.6 Hz, H_b) 7.45-7.35 (7H, m, $H_{4'}$ and other protons of phenyl rings), 7.29 (1H, m, $H_{3'}$), 7.0 (1H, ddd, J=7.5 Hz, J=4.8 Hz, J=0.6 Hz, $H_{5'}$), 6.61 (1H, s, H_4)

Figure S4. ^{1}HNMR spectrum of **H(N^N)³** in $CDCl_3$.

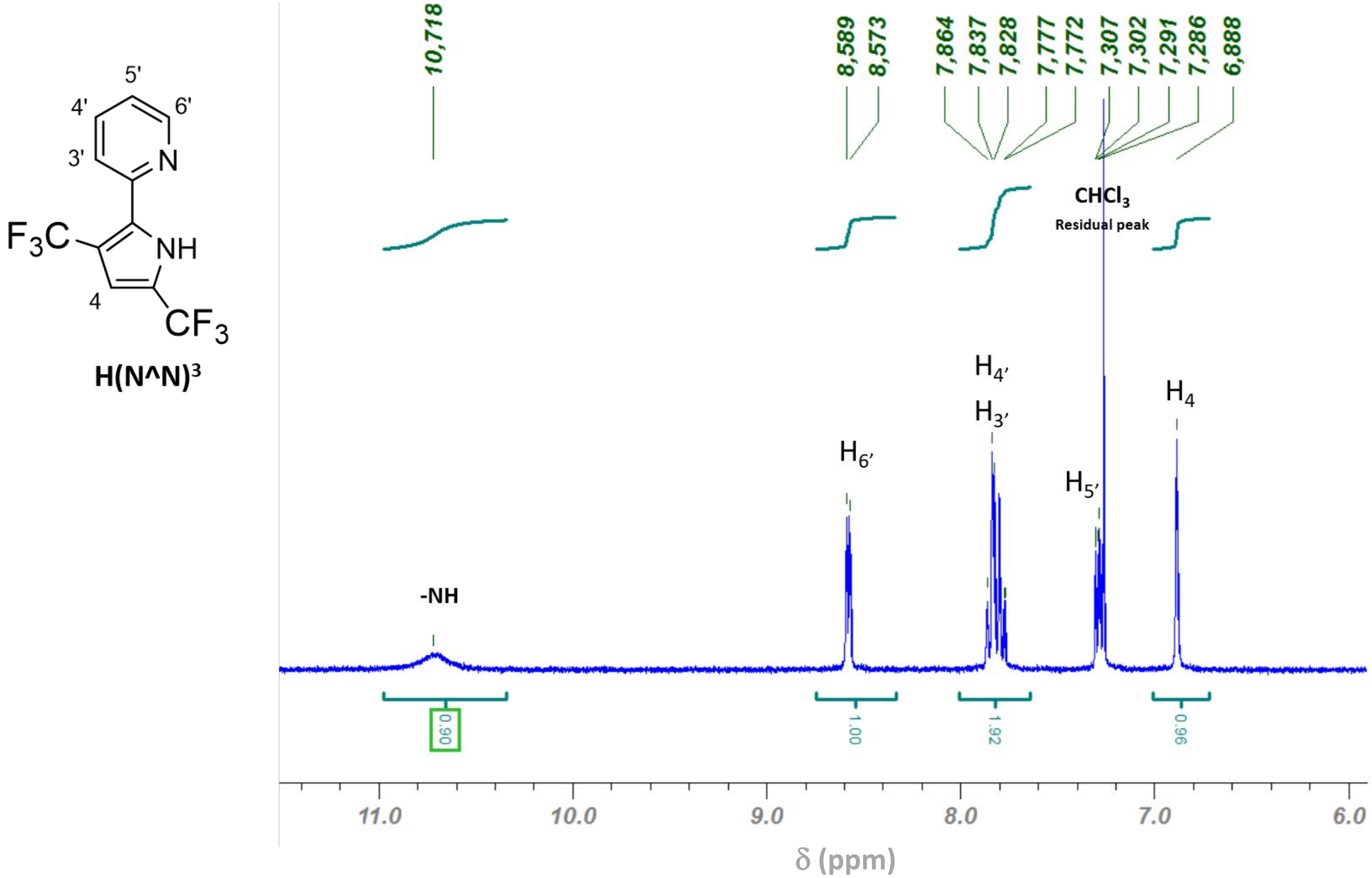

H(N^N)$_3$: ^{1}H-NMR ($CDCl_3$, 300 MHz), δ (ppm): 10.7 (1H, broad s, NH), 8.58 (1H, dd, J = 4.8 Hz, J = 0.6 Hz, $H_{6'}$), 7.9-7.7 (2H, m, $H_{4'}$ and $H_{3'}$), 7.30 (1H, m, $H_{5'}$), 6.89 (1H, s, H_4)

Figure S5. ^{1}HNMR spectrum of **1** in $CDCl_3$: full spectrum (a)**,** aromatic region with indexation (b).

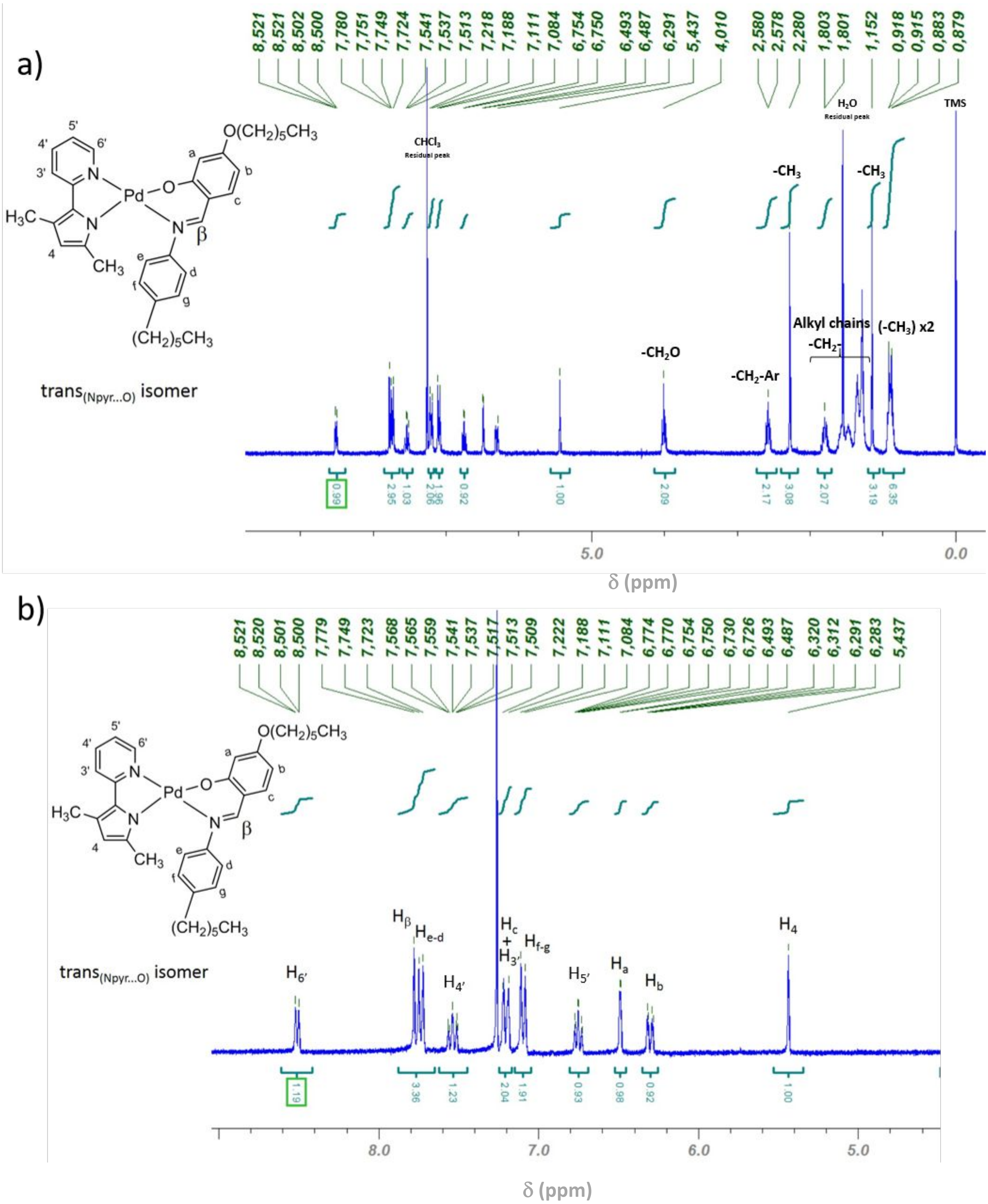

Figure S6. ^{1}HNMR spectrum of **2** in $CDCl_3$: full spectrum (a)**,** aromatic region with indexation (b).

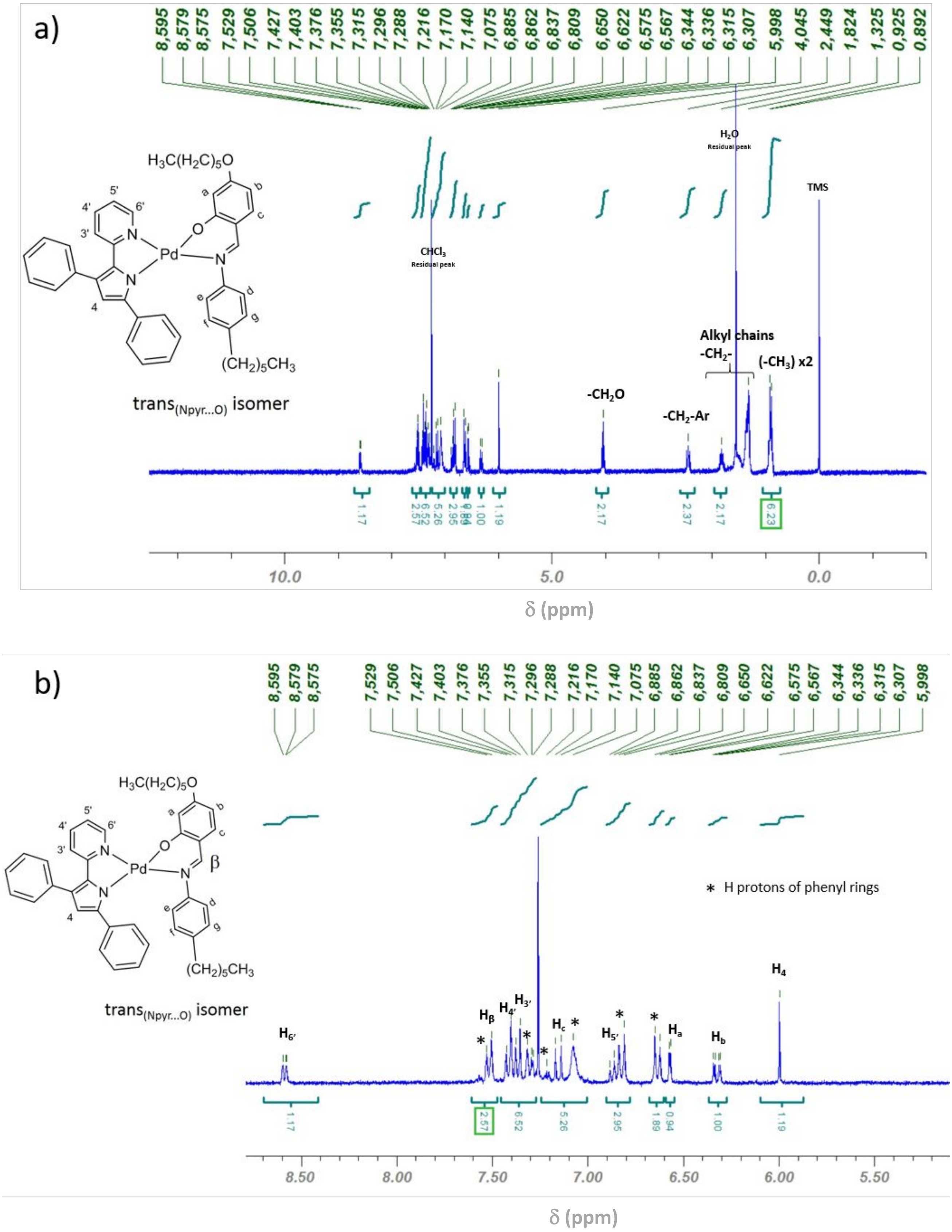

Figure S7. ^{1}HNMR spectrum of **3** in $CDCl_3$**:** full spectrum (a)**,** aromatic region with indexation (b).

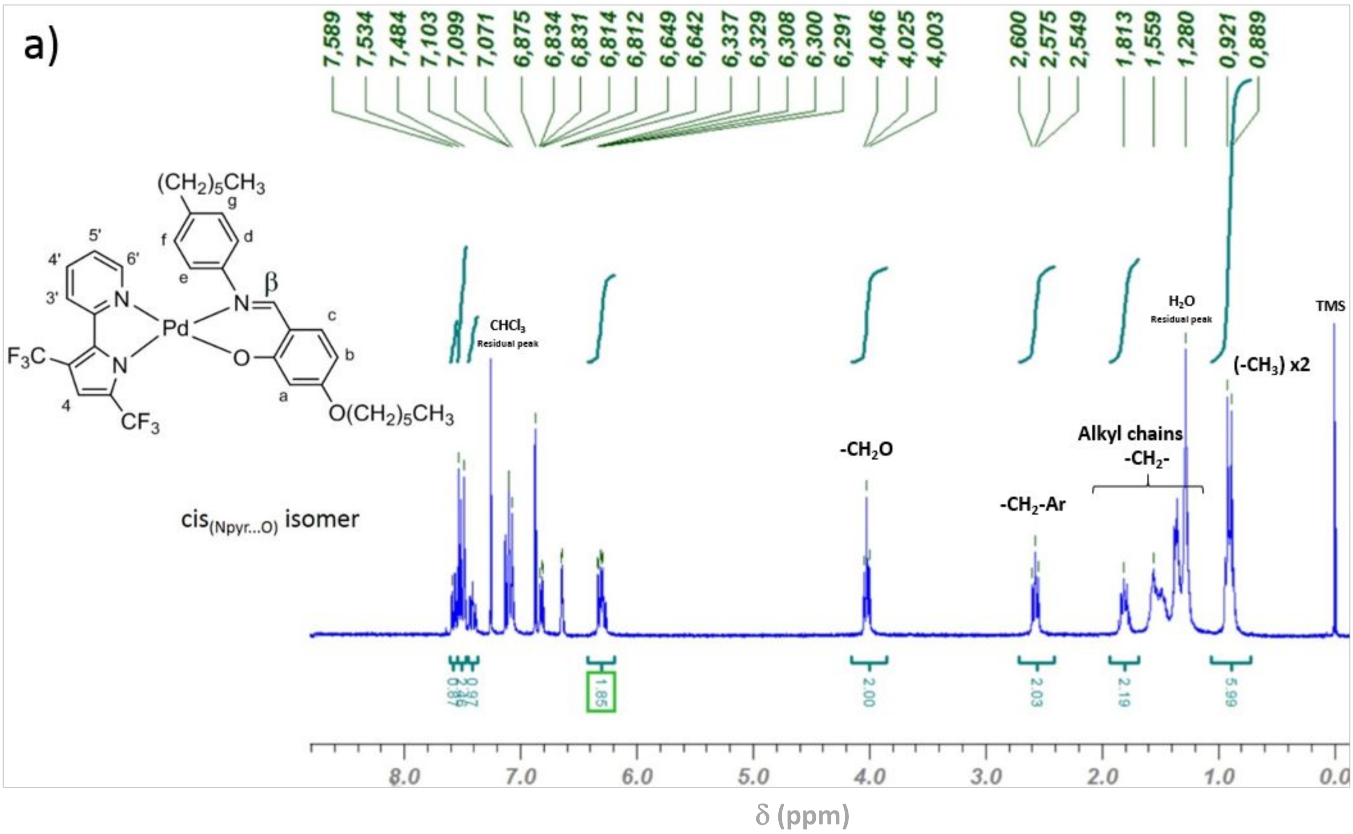

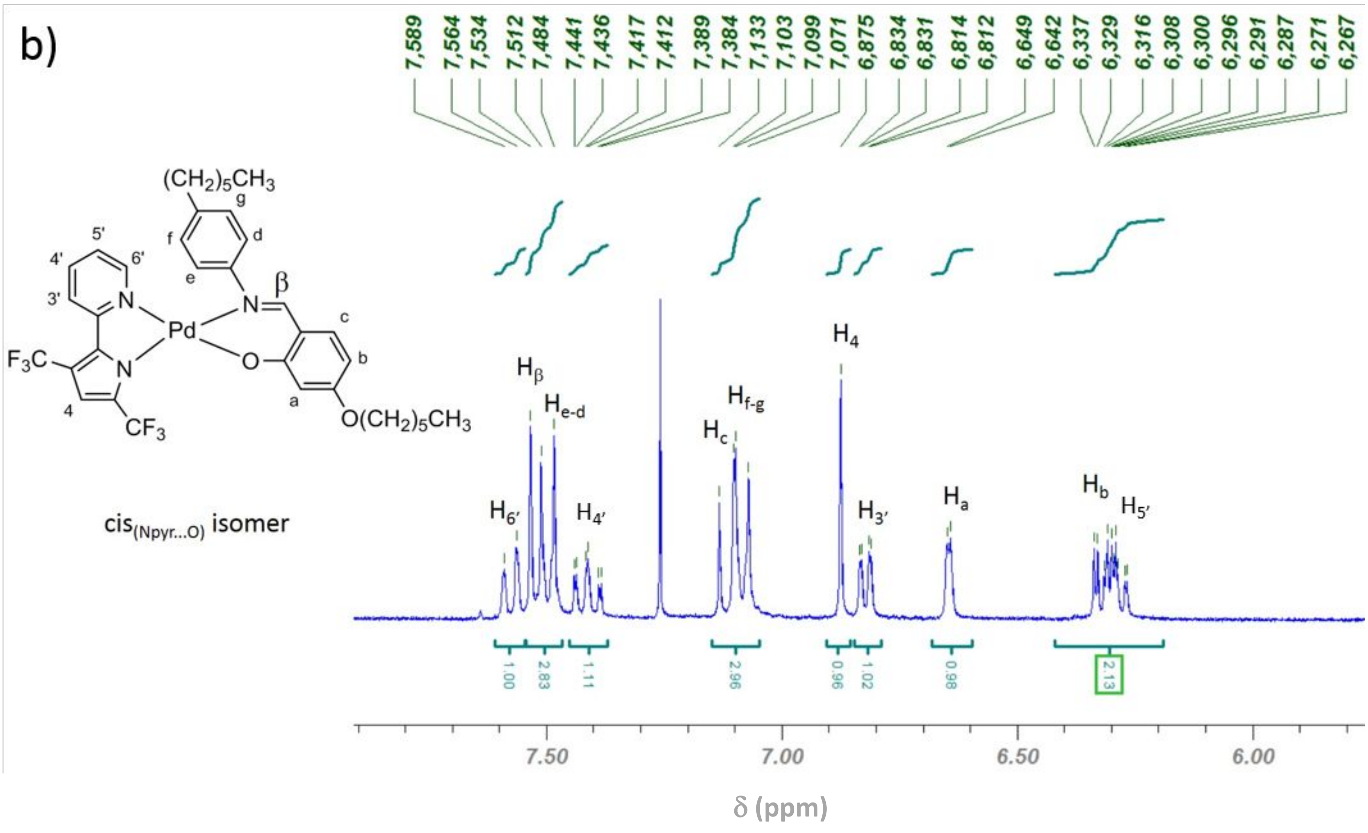

Figure S8. PXRD profiles of **1** in (a) experimental and in (b) simulated from the single X-Ray crystal structure.

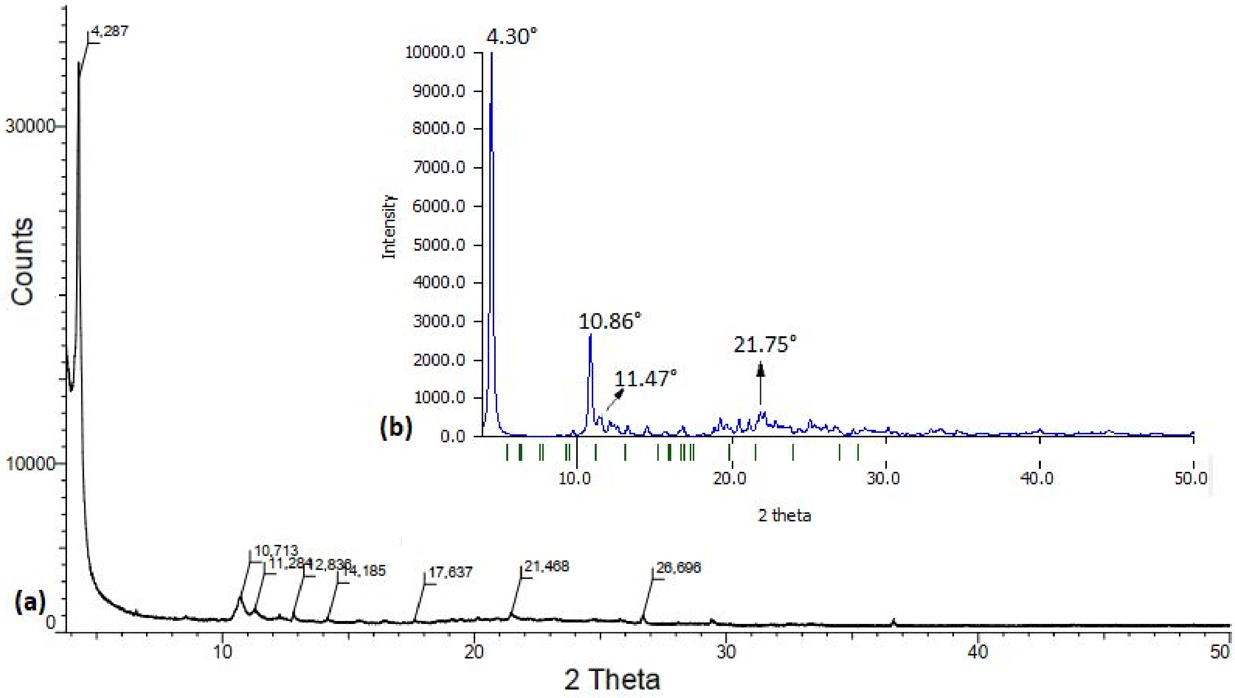

Figure S9. PXRD profiles of **3** in (a) experimental and in (b) simulated from the single X-Ray crystal structure.

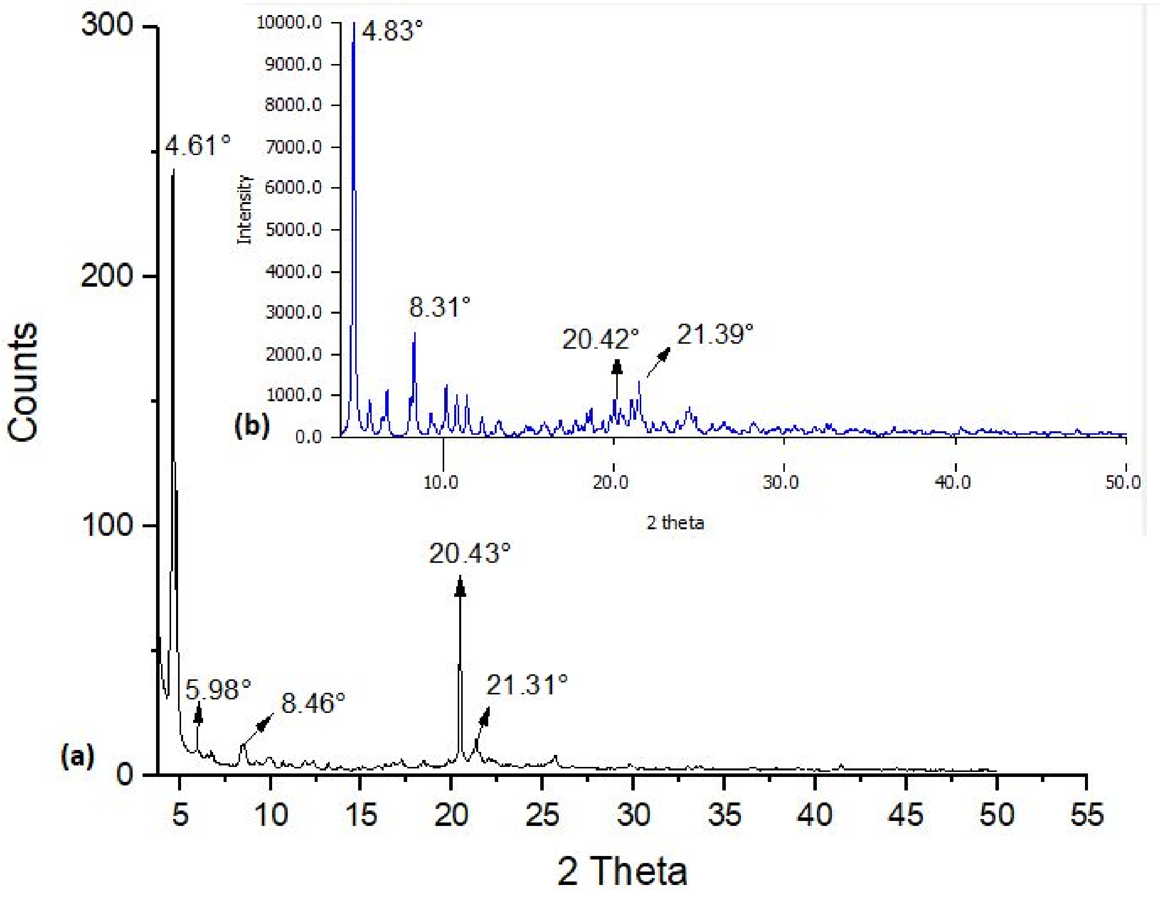

Figure S10. Crystal packing view of **3** showing the predominant C-H---F interaction [C---F^{i} 3.67(1) Å, C-H---F 165°, *i* = -x+2,-y+1,-z].

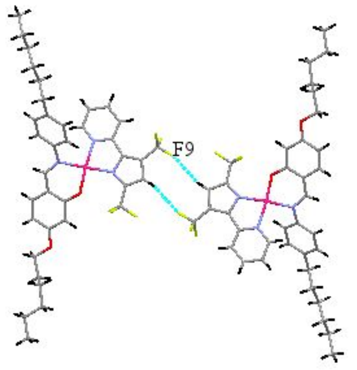

In Figure S10 is represented one of the most interesting synthon formed through C-H---F interactions in complex **3**. Two adjacent molecules are connected via C H---F weak hydrogen bonds with the complementary association of the hydrogen atoms and CF_3 groups of the pyrrolic ring of the coordinated pyridilpyrrole ligands, forming a hydrogen bond ring $R^2_2(10)$ graph set.

On the relative stability of the 1, 2 and 3 *cis*$_{(Npyrr\cdots O)}$ and *trans*$_{(Npyrr\cdots O)}$

The relative stability of the *cis*$_{(Npyrr\cdots O)}$ and *trans*$_{(Npyrr\cdots O)}$ isomers of **1**, **2** and **3** was investigated with the aim to explain the different isomery observed in **1** and **3** crystals (*trans*$_{(Npyrr\cdots O)}$ and *cis*$_{(Npyrr\cdots O)}$, respectively). Table S1 lists the relative energies of the two isomers, computed after full structure optimization in vacuum and full optimization in four solvents of different dielectric properties (chloroform, dichloromethane, ethanol and water). Solvation effects were included through the Self Consistent Reaction Field (SCRF) method described in the "Computational Methods" in the paper. These computations will be labeled as G09/SDD09/D95d when performed in vacuum and G09/M06/SDD09/D95d/SOLV in the case of SCRF computations (SOLV= chloroform, dichloromethane, ethanol and water, ε= 4.73, 8.92, 24.852, 78,3553, respectively). Figure S11 is a graphical view of the relative stabilities of the two isomers in the four studied solvents.

Table S1. Relative energies of the **1**, **2** and **3** *cis*$_{(Npyrr\cdots O)}$ and *trans*$_{(Npyrr\cdots O)}$ isomers (kJ/mol) in vacuum and different solvents. The *trans*$_{(Npyrr\cdots O)}$ isomer is always taken as reference (zero energies). Computations performed at the G09/SDD09/D95d (vacuum) and G09/SDD09/D95d/SOLV levels of approximation (SOLV=chloroform, dichloromethane, ethanol and water, ε= 4.73, 8.92, 24.852, 78,3553 respectively).

	Vacuum	$CHCl_3$	DCM	EtOH	H_2O
**1**-*cis*$_{(Npyrr\cdots O)}$	+11.0	+7.0	+5.1	+4.1	+4.7
**1**-*trans*$_{(Npyrr\cdots O)}$	0.0	0.0	0.0	0.0	0.0
**2**-*cis*$_{(Npyrr\cdots O)}$	+31.0	+24.8	+22.9	+21.2	+20.3
**2**- *trans*$_{(Npyrr\cdots O)}$	0.0	0.0	0.0	0.0	0.0
**3**-*cis*$_{(Npyrr\cdots O)}$	+8.9	+1.7	0.0	-1.0	-1.6
**3**- *trans*$_{(Npyrr\cdots O)}$	0.0	0.0	0.0	0.0	0.0

The first point to be discussed is the origin of the higher *trans*$_{(Npyrr\cdots O)}$ stability observed in vacuum. It is particularly evident in **2**, much less in **3**, however, it is a constant along the series of the studied compounds (in vacuum). For this goal, the formation process of *cis*$_{(Npyrr\cdots O)}$ and *trans*$_{(Npyrr\cdots O)}$ complexes from Pd^{2+} and the two anionic ligands was decomposed according to the following sequence of ideal steps, which is detailed below for and written for a generic complex:

$(O^\wedge N)^-_{(relaxed)} \rightarrow (O^\wedge N)^-_{(prepared)}$	Step 1
$(N^\wedge N)^-_{(relaxed)} \rightarrow (N^\wedge N)^-_{(prepared)}$	Step 2
$(O^\wedge N)^-_{(prepared)} + (N^\wedge N)^-_{(prepared)} \rightarrow [(O^\wedge N)(N^\wedge N)]^{2-}$	Step 3
$[(O^\wedge N)(N^\wedge N)]^{2-} + Pd^{2+} \rightarrow [Pd(O^\wedge N)(N^\wedge N)]$	Step 4

In the first and second steps, the (O^N) and (N^N) fragments, computed as anions, change their structure starting from the relaxed one (in vacuum) toward the structure assumed in the complex. These steps always imply a positive energy variation and are called "preparation energies" so that the deformed ligands are labeled as "prepared". The third step consists in the two prepared ligands approaching one another and reaching the relative position they undertake in the complex. Being the two fragments anionic and closed-shell, a positive energy change is expected also in this case. The fourth step consists in the formation of the metal-ligand bonds. This step includes both charge-transfer between ligands and central metal and also polarization of the electron densities induced by the interactions among fragments. It is normally associated to a negative energy term.

Figure S11. Energy of the *cis*$_{(Npyrr\cdots O)}$ form compared to the *trans*$_{(Npyrr\cdots O)}$ form in case of **1**, **2** and **3** model compounds computed in the solvent simulated by SCRF at the G09/M06/D95(d)/SDD09/M06/SOLV level of theory (SOLV=chloroform, dichloromethane, ethanol and water, ε= 4.73, 8.92, 24.852, 78,3553 respectively).

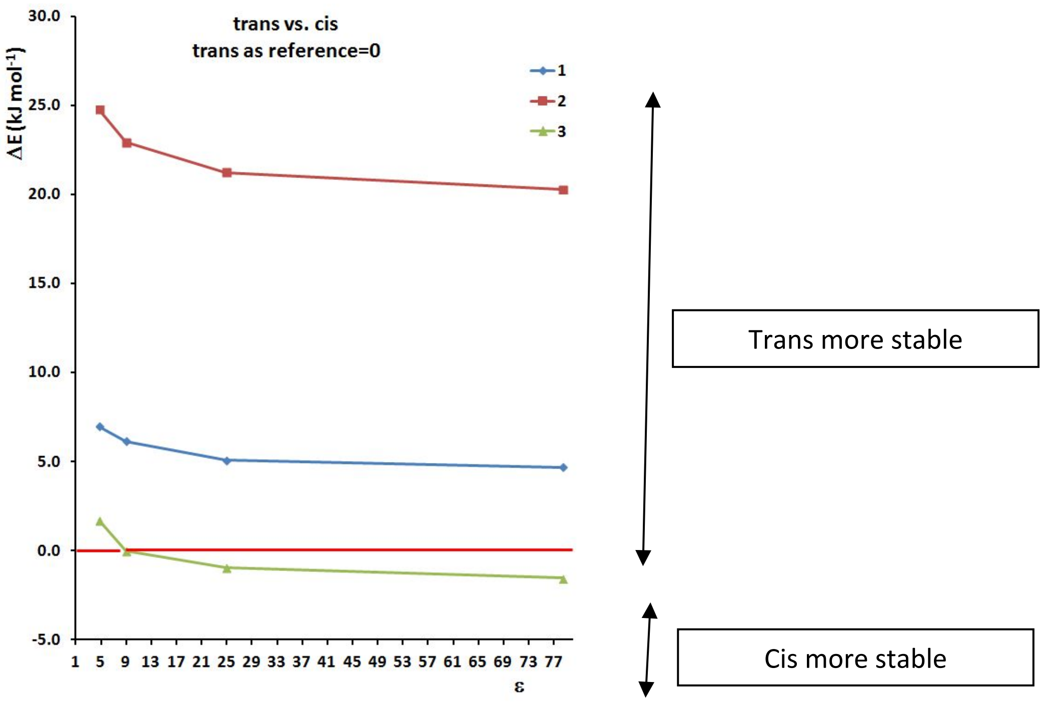

Table S2 collects the change in electronic energies of each step computed at the G09/M06/SDD09/D95d level of approximation. Step 3 was evaluated, for comparison, also at the higher level of approximation M06/6-311+G(d) in the G09/M06/6-311+G(d) column, that is, using a triple-zeta quality basis set plus one polarization (on C, O, N and F atoms) and one diffused function as provided by the program. The same step 3 was computed using the ADF2019 (Amsterdam Density Functional, 2019.03) [1] software at the M06/ATZP level, that is with a Slater-type basis set of the same quality (the standard "AUG/ATZP" basis set provided by the program, triple-zeta quality with polarization and diffused functions on all the atoms). The numerical accuracy

of the ADF computations was improved to the “good” level (“NUMERICALQUALITY good” keyword). Structures were not optimized in the G09/M06/6-311+G(d) and ADF/M06/ATZP, we used the same structures obtained at the lower G09/SDD09/D95d level. The additional computations for step 3 were performed to rule out computational reliability problems often associated to anions computations, which could be especially present in the case of two interacting anionic fragments as the case of step 3 products. Table S2 allows being confident that the low level computations are sufficiently reliable. A further reason for having performed ADF/ATZP computations is to check its similarity to the G09 computations before a further energy decomposition discussed below in this paragraph.
From Table S2, the higher stability of $trans_{(Npyrr\cdots O)}$ isomers is always mainly related to Step 3, that is, to a lower repulsive interactions between chelants in the $trans_{(Npyrr\cdots O)}$ conformation. This contribution (in favor to $trans_{(Npyrr\cdots O)}$) is only partially counterbalanced by the better metal-ligand interaction (Step 4) in the case of $cis_{(Npyrr\cdots O)}$. As often, better metal-ligand interactions imply a more positive (destabilizing) preparation energy, but no equally relevant differences in this contribution are observed between the two isomers in each compound.

Table S2. Decomposition of the ligand-metal energy bond according to Steps 1-4 described above. Electronic energy changes are collected (kJ/mol) at the G09/M06/SDD09/D95d for all the steps where not otherwise specified. Step 3 energy change was computed also at the G09/M06/6-311+G(d) and ADF/ATZP levels. Small differences between the total metal-ligand bond energies reported here respect the same value Table S1 (for example, +11.5 kJ/mol versus +11.0 kJ/mol for **1**) arises from the different way used for this computation in the two tables. In this table, the total energy is the sum of the Step 1-4 contributions, in Table S1 it is the energy difference between the two complexes. Numerical errors are different in the two computations.

	**1**-*trans*(Npyrr…O)	**1**-*cis*(Npyrr…O) minus **1**-*trans*(Npyrr…O) (Energy Variation)
Step 1	+25.1	-4.0
Step 2	+34.1	+7.6
Total Preparation Energy	+59.2	+3.5
Step 3	+277.4<br>+270.8 (G09/M06/6-311+G(d))<br>+269.4 (ADF/M06/ATZP)	+25.0<br>+24.4 (G09/M06/6-311+G(d))<br>+23.3 (ADF/M06/ATZP)
Step 4	-3223.5	-17.0
Total metal-ligand bond energy	-2886.8	+11.5

	**2**-*trans*(Npyrr…O)	**2**-*cis*(Npyrr…O) minus **2**-*trans*(Npyrr…O) (Energy Variation)
Step 1	+40.5	-4.4
Step 2	+33.4	+8.3
Total Preparation Energy	+73.9	+3.9
Step 3	+245.5<br>+236.7 (G09/M06/6-311+g(d))<br>+241.5 (ADF/M06/ATZP)	+40.3<br>+38.6 (G09/M06/6-311+g(d))<br>+33.7 (ADF/M06/ATZP)
Step 4	-3124.1	-11.9
Total metal-ligand bond energy	-2804.8	+32.3

	**3**-*trans*(Npyrr…O)	**3**-*cis*(Npyrr…O) minus **3**-*trans*(Npyrr…O) (Energy Variation)
Step 1	+28.5	-6.6
Step 2	+38.5	+5.3
Total Preparation Energy	+67.1	-1.2
Step 3	+257.7<br>+253.1 (G09/M06/6-311+g(d))<br>+256.1 (ADF/M06/ATZP)	+31.2<br>+29.8 (G09/M06/6-311+g(d))<br>+27.7 (ADF/M06/ATZP)
Step 4	-3099.5	-20.8
Total metal-ligand bond energy	-2774.8	+9.2

For further understanding, step 3 was analyzed according to the decomposition scheme of the bonding energy implemented in the ADF program.[1,2] The interaction energy between the two anionic ligands (as they place one another in the complex) is decomposed in three contributions: electrostatic repulsion, Pauli repulsion (repulsion between closed-shell electron densities due to the antisymmetric character of the state function of a system of electrons, roughly associated to the two orbitals-four electrons interactions and commonly called steric repulsion) and polarization of the interacting fragments plus charge transfer between themselves. Regarding the last step, charge transfer between the two anionic ligands is almost inexistent, for obvious reasons, so this term can be considered only polarization of the two ligands due to mostly electrostatic interactions.
According to Table S3, the larger repulsion between the two ligands is associated to a larger electrostatic repulsion. The changes of Pauli repulsion and polarization energies (passing from the $trans_{(Npyrr\cdots O)}$ isomer to $cis_{(Npyrr\cdots O)}$ isomer) sum to a relatively small value in all the cases.

Table S3. Decomposition of the interaction energy (energy term) associated to Step 3 only. Energies are kJ/mol.

	**1**-*trans*(Npyrr…O)	**1**-*cis*(Npyrr…O) minus **1**-*trans*(Npyrr…O) (Energy Variation)
Electrostatic	+281.7	+20.2
Pauli Repulsion	+44.5	+1.4
Polarization and Charge Transfer	-56.8	+1.6
Total	+269.4	+23.3
	**2**-*trans*(Npyrr…O)	**2**-*cis*(Npyrr…O) minus **2**-*trans*(Npyrr…O) (Energy Variation)
Electrostatic	+256.4	+30.5
Pauli Repulsion	+43.4	+6.1
Polarization and Charge Transfer	-58.3	-2.9
Total	+241.5	+33.7
	**3**-*trans*(Npyrr…O)	**3**-*cis*(Npyrr…O) minus **3**-*trans*(Npyrr…O) (Energy Variation)
Electrostatic	+263.3	+16.6
Pauli Repulsion	+45.0	+9.3
Polarization and Charge Transfer	-52.2	-9.9
Total	+256.1	+27.7

Our conclusion is that in the $cis_{(Npyrr\cdots O)}$ isomer, the proximity between the formally negative oxygen and nitrogen atoms of the (O^N) and (N^N) ligands leads to a higher repulsion between the chelants. Figure S12 shows the SCF Coulomb potential computed for the two isomers of **1** and plotted on the surface corresponding to a 0.03 bohr^{-3} electron density. It is possible to observe that the pyrrole cycle bears more negative charge in comparison to the pyridine one. On the (O^N) ligand, the oxygen atom shows a particularly negative charge.
As detailed in the paper, $cis_{(Npyrr\cdots O)}$ isomers are favored in condensed phases by their higher dipole moment. Figure S12 allows a direct association between dipole moment and SCF charge distribution in the two isomers. In the **1** $trans_{(Npyrr\cdots O)}$ isomer, the positive Pd atom is in between the (O^N) oxygen and (N^N) pyrrole donor atoms. This fact is expected to lead to a smaller dipole moment in comparison to what happens in the $cis_{(Npyrr\cdots O)}$ isomer, where and bent disposition for the N_{pyrr}-Pd-O sequence of atoms is present.

Figure S12. SCF Coulomb potential (atomic units) plotted on the surface corresponding to the electron density of 0.03 bohr^{-3}. The **1** *trans*$_{(Npyrr\cdots O)}$ (top) and *cis*$_{(Npyrr\cdots O)}$ (bottom) isomers are sketched.

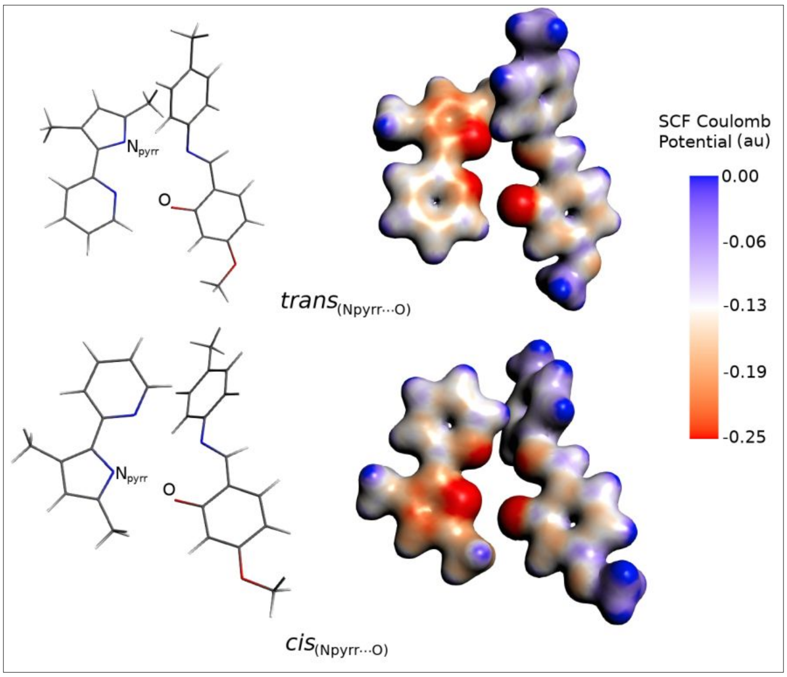

Experimental *vs.* computed chemical shifts of 1-3 *cis* and *trans* isomers

Figure S13. Plot of the experimental vs. computed chemical shifts(ppm) for compound **2** for the cis$_{(Npyr....O)}$ and trans$_{(Npyr....O)}$ isomers. Geometries were optimized at M06/ECP28MDF:cc-aug-VQZPP/cc-pVQZ/CLF. Chemical shifts were computed at this geometry with M06, B3PW91 and mPW1PW91 (red, green and violet respectively) xc-functionals and ECP28MDF:cc-aug-VQZPP/cc-pVTZ/CLF basis set. Further calculations including relativistic contributions were performed with ZORA approximation and mPW1PW91/STO-TZPP level of theory (black triangle and full circle).

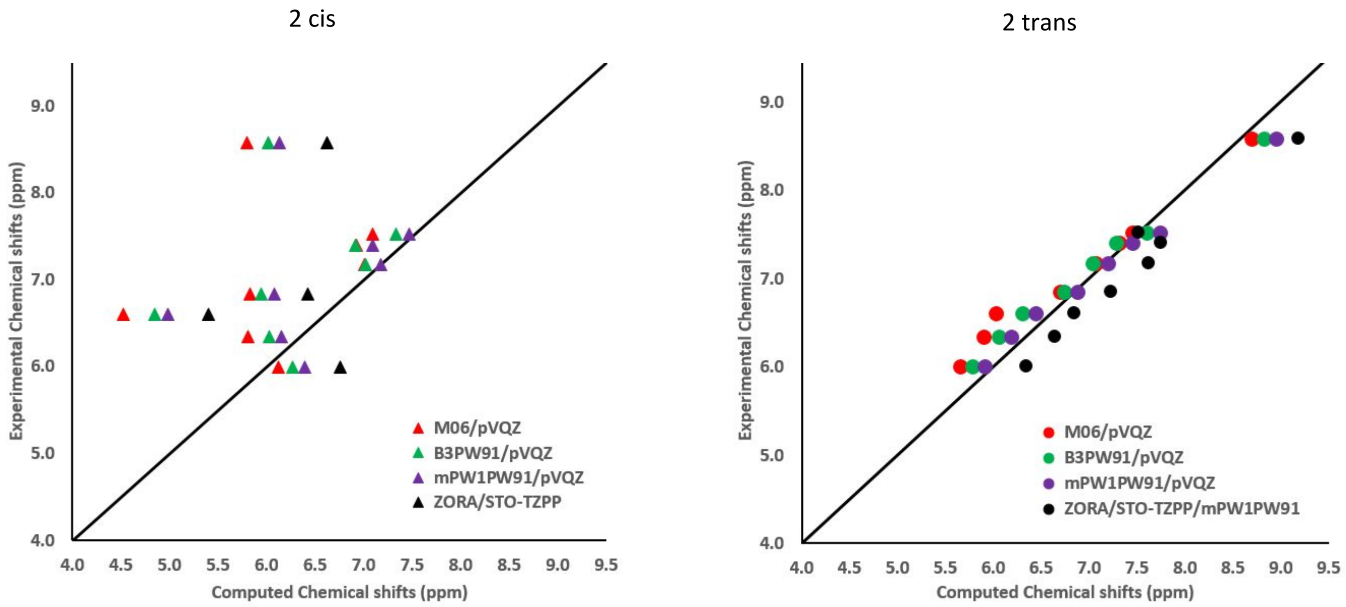

Figure S14. Plot of the experimental vs. computed chemical shifts (ppm) for compound **1** for the *cis*$_{(Npyr....O)}$ and *trans*$_{(Npyr....O)}$ isomers. Geometries were optimized at M06/ECP28MDF:cc-aug-VDZPP/cc-pVDZ/CLF. Chemical shifts were computed at this geometry but with M06, B3PW91 and mPW1PW91 (red, green and violet respectively) xc-functionals and DFT/ECP28MDF:cc-aug-VTZPP/cc-pVTZ/CLF level of theory.

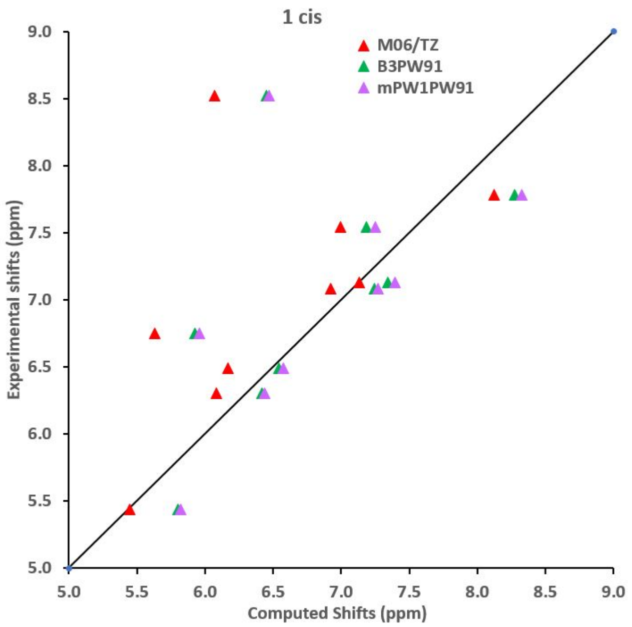

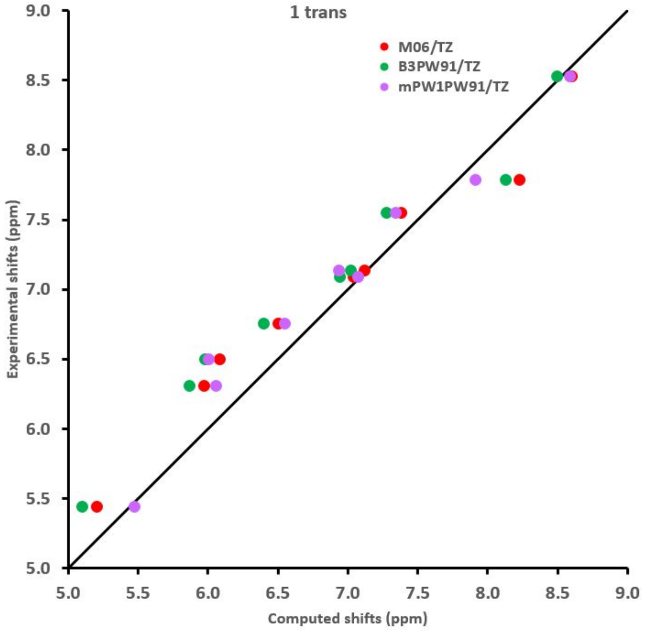

Figure S15. Plot of the experimental vs. computed chemical shifts(ppm) for compound **3** for the *cis*$_{(Npyr....O)}$ and *trans*$_{(Npyr....O)}$ isomers. Geometries were optimized at M06/ECP28MDF:cc-aug-VDZPP/cc-pVDZ/CLF. Chemical shifts were computed at this geometry but with M06, B3PW91 and mPW1PW91 (red, green and violet respectively) xc-functionals and ECP28MDF:cc-aug-VTZPP/cc-pVTZ/CLF basis set.

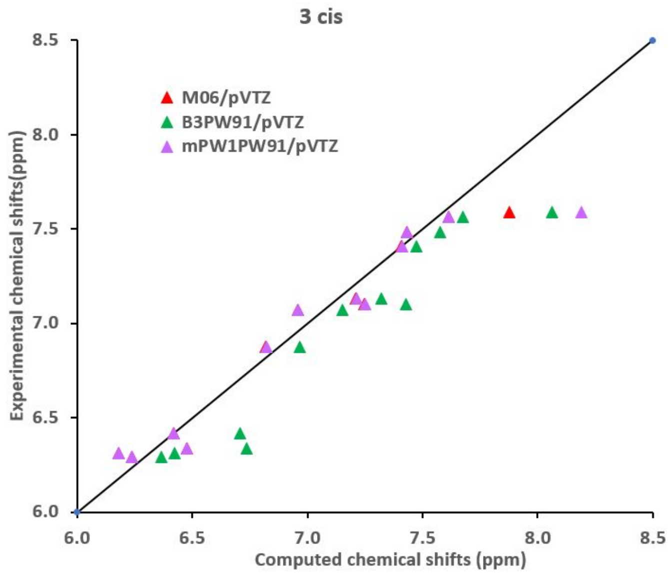

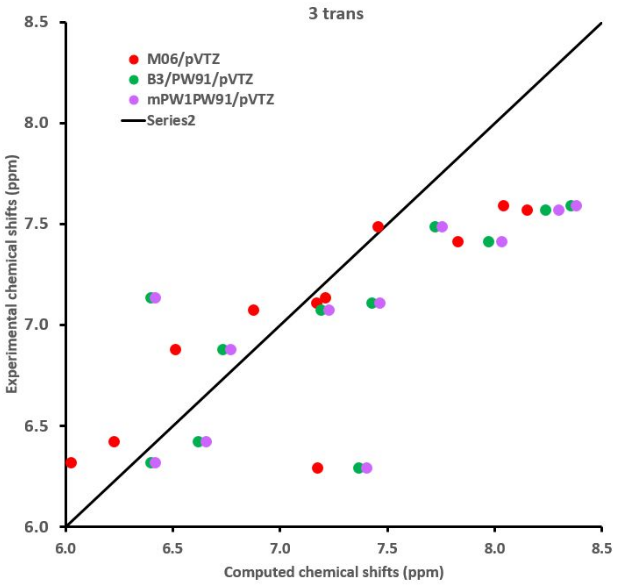

Figure S14 shows that, in case of **1**, the agreement between the computed and experimental chemical shifts are better for the *trans* isomer than in case of *cis*. The other way around is true in case of the compound **3** where the agreement (Figure S15) is better for the *cis* than for the *trans* isomer. Both calculations agree with the SCXRD findings validating this approach also in case of compound **2** (Figure S13) where SCXRD data aren't available as discussed in the paper. From Figure S13 it is evident as in case of **1** that the best agreement is for isomer *trans* than *cis*.

Figure S16. Spin density of the cationic forms of **1** (a), **2** (b) and **3** (c) and corresponding anionic forms (d-f); α-HOMO of the anionic **1**, **2** and **3** (g-i). Computations performed at the PW1PW91/SDD09/D95D/DCM level of approximation.

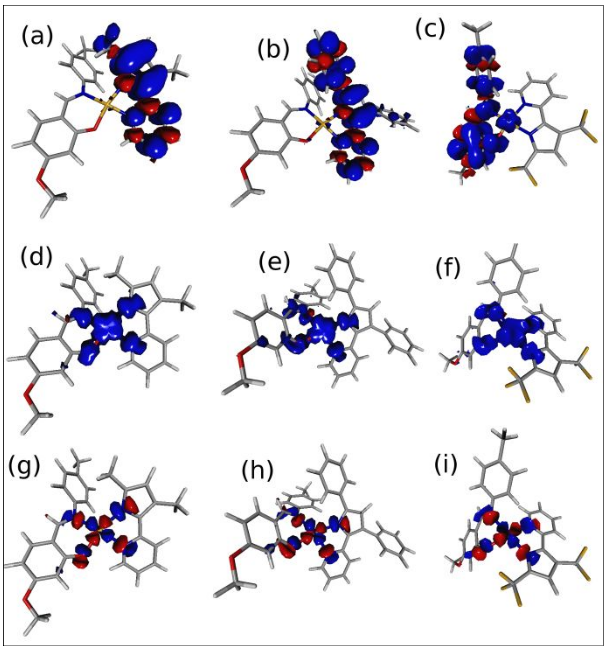

Figure S17. Some of the MOs of **1**, **2** and **3** computed at the mPW1PW91/SDD09/D95d/DCM level of theory involved in the most relevant transitions of the UV-Vis spectrum (all surfaces are drawn at 0.02 bohr$^{-3/2}$).

1	2	3
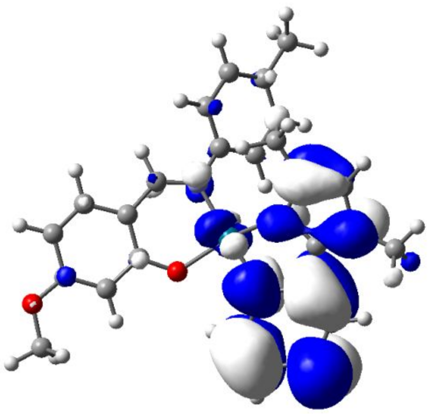	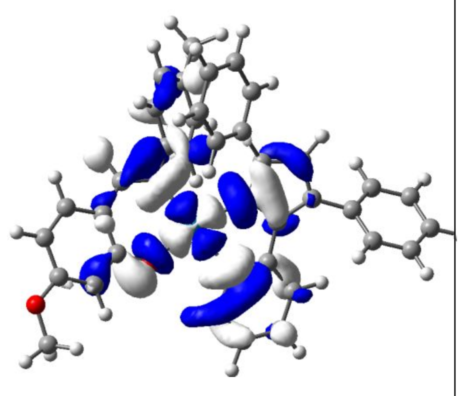	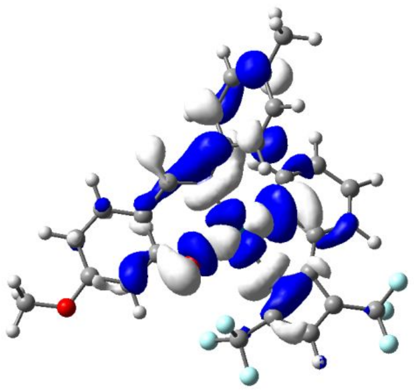
LUMO+2 -0.04646 hartree	LUMO+2 -0.04773 hartree	LUMO+2 -0.05702 hartree

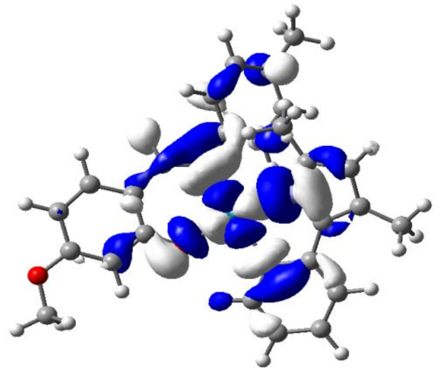	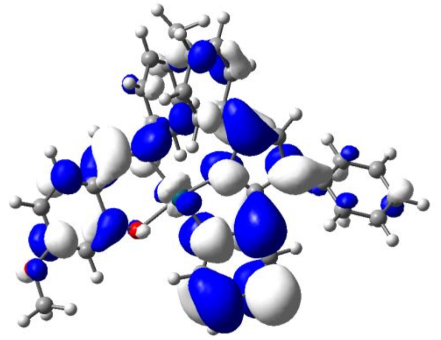	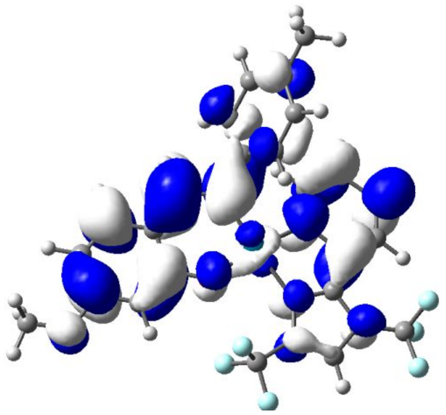
LUMO+1 -0.04989 hartree	LUMO+1 -0.06255 hartree	LUMO+1 -0.06572 hartree
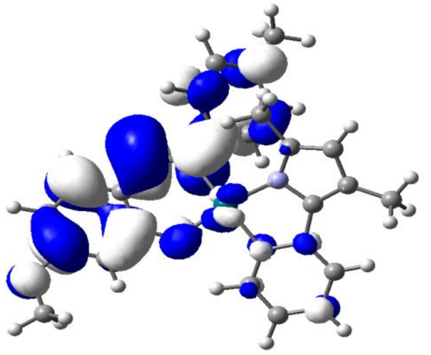	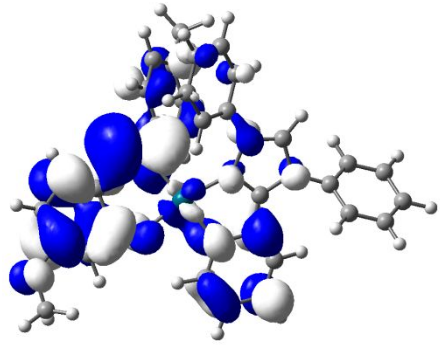	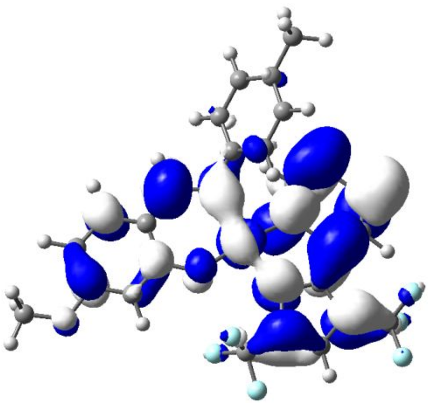
LUMO -0.06267 hartree	LUMO -0.06220 hartree	LUMO -0.06903 hartree
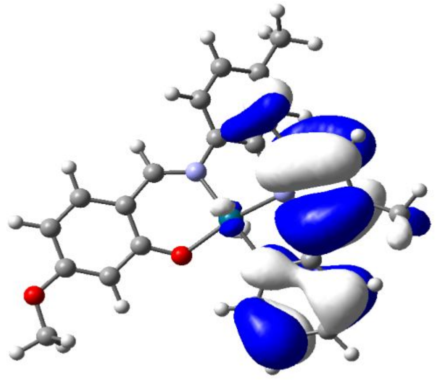	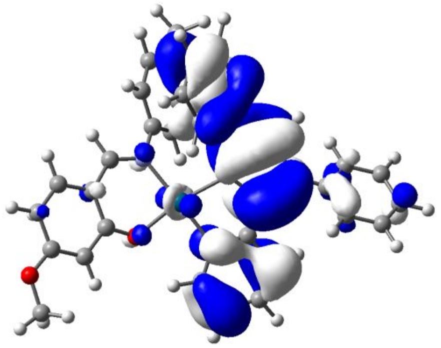	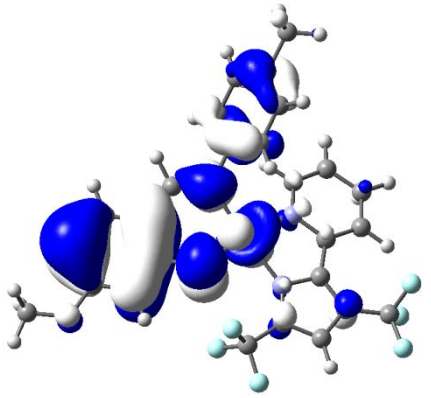
HOMO -0.19407 hartree	HOMO -0.19768 hartree	HOMO -0.22193 hartree

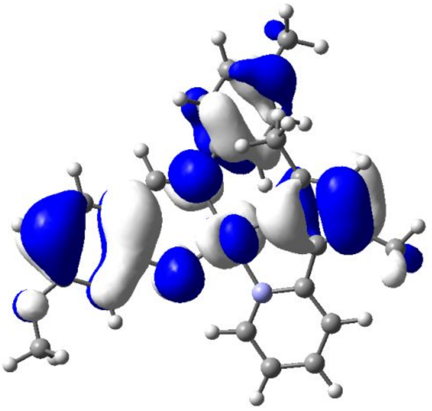	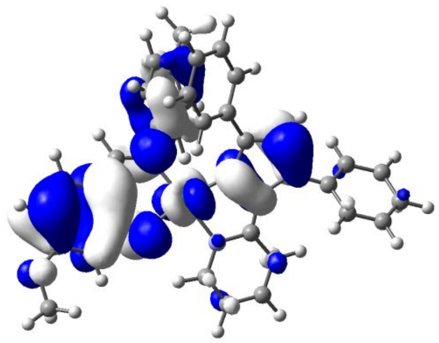	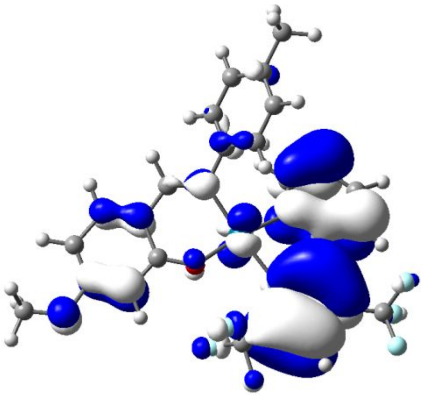
HOMO-1 -0.21185 hartree	HOMO-1 -0.21290 hartree	HOMO-1 -0.23367 hartree

Figure S18. Electronic absorption spectra of **1**-**3** in dichloromethane solution.

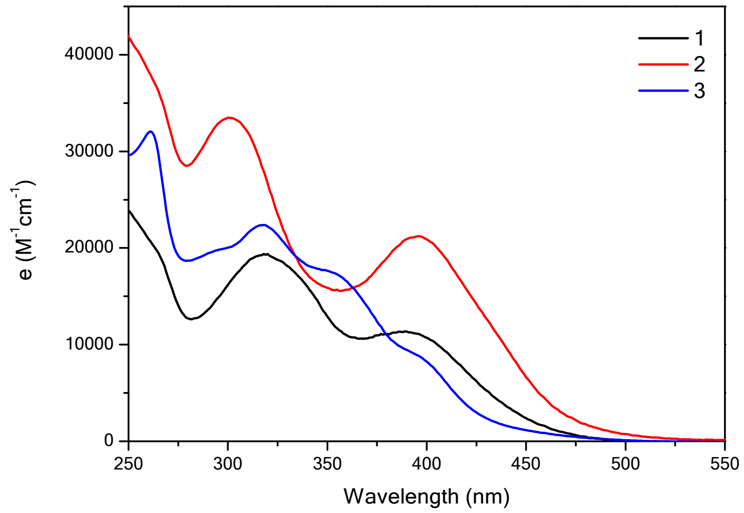

Table S4. Absorption data of **1**-**3**recorded in dichloromethane solution

Compound	λ/nm(ε,$M^{-1}cm^{-1}$)
**1**	320 (19342), 390 (11315)
**2**	300 (34285), 396 (21714)
**3**	261 (31294), 317 (21823), 350(17200, sh), 395 (8600, sh)

Figure S19. UV-Vis spectra in dichloromethane of **1** (black),**2** (red) and **3** (green) and the computed transitions by TD-DFT at the mPW1PW91/SDD09/D95d/DCM level of theory (vertical arrows, same colour convention). (The number of computed states has been extended up to 32500, 36000, 42500 cm^{-1} for **1, 2** and **3** respectively).

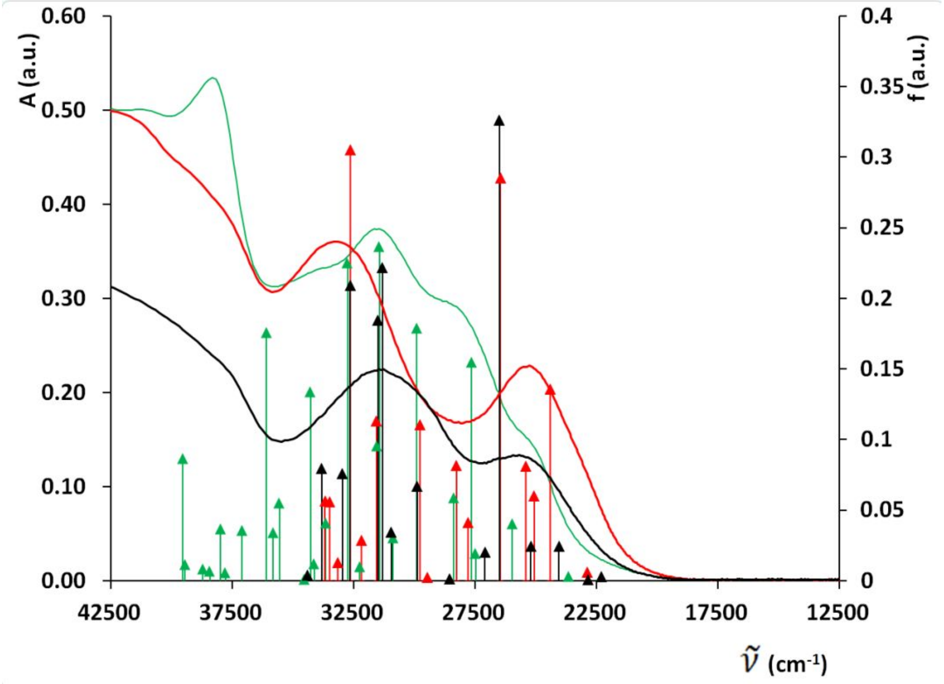

Table S5. Computed Singlet excited states at the mPW1PW91/D95(d)SDD09/DCM level of theory for **1**, **2** and **3**. Structure optimizations at the same level of approximation were performed before TD-DFT computations. Number on the left indicates orbital involved in the excitation, coefficient of the Slater determinant defining the excitation, % contribution of the same determinant and occupied and virtual MOs which defines the Slater determinant (MOs on the last two columns are indicated numbering HOMO on the left of the arrow and LUMO on the right of the arrow as 0).

Complex 1

HOMO is orbital 118 and LUMO is orbital 119

>>>> Singlet states <<<<

eV	nm	cm^{-1}	f
2.7646	448.47	22298.0	0.0026
2.8311	437.93	22834.7	0.0008
2.9805	415.98	24039.6	0.0246
3.1243	396.84	25199.1	0.0244
3.2845	377.48	26491.5	0.3263
3.3570	369.33	27076.1	0.0201
3.5377	350.47	28533.1	0.0014
3.7032	334.80	29868.6	0.0668
3.8368	323.15	30945.4	0.0341
3.8807	319.49	31299.9	0.2220
3.9036	317.62	31484.2	0.1843
4.0467	306.38	32639.2	0.2089
4.0853	303.49	32950.0	0.0756
4.1914	295.81	33805.5	0.0796
4.2631	290.83	34384.3	0.0038

1 Excited State: Singlet
2.7646 eV 448.47 nm 22298.cm^{-1} f=0.0026
108 -> 120 -0.11777 2.77 % -10 -> 1
110 -> 120 0.12731 3.24 % -8 -> 1
116 -> 120 -0.15032 4.52 % -2 -> 1
117 -> 119 -0.21102 8.91 % -1 -> 0
117 -> 120 0.56414 63.65 % -1 -> 1
118 -> 120 0.20767 8.63 % 0 -> 1

2 Excited State: Singlet
2.8311 eV 437.93 nm 22835.cm^{-1} f=0.0008
117 -> 120 -0.18429 6.79 % -1 -> 1
118 -> 119 -0.28254 15.97 % 0 -> 0
118 -> 120 0.59523 70.86 % 0 -> 1

3 Excited State: Singlet
2.9805 eV 415.98 nm 24040.cm^{-1} f=0.0246
118 -> 119 0.63348 80.26 % 0 -> 0
118 -> 120 0.29135 16.98 % 0 -> 1

4 Excited State: Singlet
3.1243 eV 396.84 nm 25199.cm^{-1} f=0.0244
113 -> 120 0.10132 2.05 % -5 -> 1
114 -> 120 0.43185 37.30 % -4 -> 1
117 -> 119 0.40872 33.41 % -1 -> 0
117 -> 120 0.12271 3.01 % -1 -> 1
118 -> 121 0.27955 15.63 % 0 -> 2

5 Excited State: Singlet
3.2845 eV 377.48 nm 26491.cm^{-1} f=0.3263
114 -> 120 0.11811 2.79 % -4 -> 1
117 -> 119 -0.44151 38.99 % -1 -> 0
117 -> 120 -0.12858 3.31 % -1 -> 1
118 -> 121 0.48258 46.58 % 0 -> 2
118 -> 122 -0.10270 2.11 % 0 -> 3

6 Excited State: Singlet
3.3570 eV 369.33 nm 27076.cm^{-1} f=0.0201
113 -> 120 0.11530 2.66 % -5 -> 1
114 -> 120 0.45539 41.48 % -4 -> 1
117 -> 119 -0.26622 14.17 % -1 -> 0
117 -> 120 -0.11660 2.72 % -1 -> 1
118 -> 121 -0.38072 28.99 % 0 -> 2
118 -> 122 0.11496 2.64 % 0 -> 3

7 Excited State: Singlet
3.5377 eV 350.47 nm 28533.cm^{-1} f=0.0014
110 -> 120 -0.19923 7.94 % -8 -> 1
111 -> 120 -0.31360 19.67 % -7 -> 1
114 -> 120 0.14910 4.45 % -4 -> 1
115 -> 120 0.12377 3.06 % -3 -> 1
116 -> 119 -0.15272 4.66 % -2 -> 0
116 -> 120 0.47418 44.97 % -2 -> 1
117 -> 120 0.15927 5.07 % -1 -> 1

8 Excited State: Singlet
3.7032 eV 334.80 nm 29869.cm^{-1} f=0.0668
116 -> 119 0.14151 4.01 % -2 -> 0
116 -> 121 -0.17176 5.90 % -2 -> 2
117 -> 121 0.65089 84.73 % -1 -> 2

9 Excited State: Singlet
3.8368 eV 323.15 nm 30945.cm^{-1} f=0.0341
106 -> 120 0.13519 3.66 % -12 -> 1
107 -> 120 0.20956 8.78 % -11 -> 1
109 -> 120 0.20816 8.67 % -9 -> 1
111 -> 120 0.34700 24.08 % -7 -> 1
115 -> 120 -0.31337 19.64 % -3 -> 1
116 -> 119 -0.24315 11.82 % -2 -> 0
116 -> 120 0.15686 4.92 % -2 -> 1
117 -> 121 0.13580 3.69 % -1 -> 2
118 -> 122 0.14334 4.11 % 0 -> 3

10 Excited State: Singlet
3.8807 eV 319.49 nm 31300.cm^{-1} f=0.2220
107 -> 120 0.17560 6.17 % -11 -> 1
109 -> 120 0.22238 9.89 % -9 -> 1
110 -> 120 -0.16726 5.60 % -8 -> 1
116 -> 119 0.56247 63.27 % -2 -> 0
116 -> 120 0.12198 2.98 % -2 -> 1
117 -> 121 -0.11410 2.60 % -1 -> 2

11 Excited State: Singlet
3.9036 eV 317.62 nm 31484.cm^{-1} f=0.1843
116 -> 119 0.17720 6.28 % -2 -> 0
118 -> 121 0.14648 4.29 % 0 -> 2
118 -> 122 0.63619 80.95 % 0 -> 3

12 Excited State: Singlet
4.0467 eV 306.38 nm 32639.cm^{-1} f=0.2089
107 -> 120 -0.22801 10.40 % -11 -> 1
109 -> 120 -0.24632 12.13 % -9 -> 1
110 -> 120 0.19130 7.32 % -8 -> 1
113 -> 120 0.10327 2.13 % -5 -> 1
115 -> 119 0.25162 12.66 % -3 -> 0
115 -> 120 -0.23178 10.74 % -3 -> 1
116 -> 119 0.14628 4.28 % -2 -> 0
116 -> 120 0.34651 24.01 % -2 -> 1
117 -> 120 0.12583 3.17 % -1 -> 1

13 Excited State: Singlet
4.0853 eV 303.49 nm 32950.cm^{-1} f=0.0756
111 -> 120 -0.13440 3.61 % -7 -> 1
114 -> 119 -0.17605 6.20 % -4 -> 0
115 -> 119 0.59620 71.09 % -3 -> 0
115 -> 120 0.10643 2.27 % -3 -> 1
116 -> 120 -0.13356 3.57 % -2 -> 1

14 Excited State: Singlet
4.1914 eV 295.81 nm 33805.cm^{-1} f=0.0796
114 -> 119 0.64997 84.49 % -4 -> 0
114 -> 120 0.12503 3.13 % -4 -> 1
115 -> 119 0.18605 6.92 % -3 -> 0

15 Excited State: Singlet
4.2631 eV 290.83 nm 34384.cm^{-1} f=0.0038
118 -> 123 0.70461 99.30 % 0 -> 4

Complex 2

HOMO is orbital 150 and LUMO is orbital 151

>>>> Singlet states <<<<

eV	nm	cm^{-1}	f
2.8359	437.20	22872.8	0.0058
3.0240	410.00	24390.2	0.1359
3.1059	399.19	25050.7	0.0600
3.1486	393.78	25394.9	0.0809
3.2794	378.07	26450.1	0.2853
3.4442	359.98	27779.3	0.0411
3.5026	353.97	28251.0	0.0818
3.6507	339.62	29444.7	0.0024
3.6890	336.09	29753.9	0.1106
3.9117	316.96	31549.7	0.1132
3.9887	310.84	32170.9	0.0288
4.0450	306.52	32624.3	0.3055
4.1094	301.71	33144.4	0.0128
4.1505	298.72	33476.2	0.0561
4.1749	296.97	33673.4	0.0566

1 Excited State: Singlet
2.8359 eV 437.20 nm 22873.cm^{-1} f=0.0058
137 -> 153 0.14992 4.50 % -13 -> 2
140 -> 153 0.11305 2.56 % -10 -> 2
148 -> 153 -0.13352 3.57 % -2 -> 2
149 -> 151 -0.20639 8.52 % -1 -> 0
149 -> 153 0.49393 48.79 % -1 -> 2
150 -> 151 -0.15582 4.86 % 0 -> 0
150 -> 153 0.31647 20.03 % 0 -> 2

2 Excited State: Singlet
3.0240 eV 410.00 nm 24390.cm^{-1} f=0.1359
149 -> 153 0.20095 8.08 % -1 -> 2
150 -> 151 0.63179 79.83 % 0 -> 0
150 -> 153 -0.15820 5.01 % 0 -> 2

3 Excited State: Singlet
3.1059 eV 399.19 nm 25051.cm^{-1} f=0.0600
149 -> 151 0.18338 6.73 % -1 -> 0
149 -> 153 -0.16493 5.44 % -1 -> 2
150 -> 151 0.25065 12.57 % 0 -> 0
150 -> 153 0.58633 68.76 % 0 -> 2

4 Excited State: Singlet
3.1486 eV 393.78 nm 25395.cm^{-1} f=0.0809
146 -> 153 -0.21230 9.01 % -4 -> 2
149 -> 151 -0.26940 14.52 % -1 -> 0
149 -> 153 -0.18600 6.92 % -1 -> 2
150 -> 152 0.53729 57.74 % 0 -> 1

5 Excited State: Singlet
3.2794 eV 378.07 nm 26450.cm^{-1} f=0.2853
146 -> 153 0.18628 6.94 % -4 -> 2
148 -> 153 -0.10448 2.18 % -2 -> 2
149 -> 151 0.47391 44.92 % -1 -> 0
149 -> 153 0.11876 2.82 % -1 -> 2
150 -> 152 0.42215 35.64 % 0 -> 1

6 Excited State: Singlet
3.4442 eV 359.98 nm 27779.cm^{-1} f=0.0411
142 -> 153 0.11474 2.63 % -8 -> 2
145 -> 153 0.20680 8.55 % -5 -> 2
146 -> 153 0.46140 42.58 % -4 -> 2
147 -> 153 -0.10072 2.03 % -3 -> 2
149 -> 151 -0.27826 15.49 % -1 -> 0
149 -> 152 0.20281 8.23 % -1 -> 1
149 -> 153 -0.16007 5.12 % -1 -> 2

7 Excited State: Singlet
3.5026 eV 353.97 nm 28251.cm^{-1} f=0.0818
146 -> 153 -0.15334 4.70 % -4 -> 2
148 -> 152 -0.11768 2.77 % -2 -> 1
149 -> 151 0.17104 5.85 % -1 -> 0
149 -> 152 0.63338 80.23 % -1 -> 1

8 Excited State: Singlet
3.6507 eV 339.62 nm 29445.cm^{-1} f=0.0024
138 -> 153 -0.16692 5.57 % -12 -> 2
139 -> 153 0.25517 13.02 % -11 -> 2
140 -> 153 0.16805 5.65 % -10 -> 2
146 -> 153 0.18970 7.20 % -4 -> 2

147 -> 153 0.13679 3.74 % -3 -> 2
148 -> 151 -0.18711 7.00 % -2 -> 0
148 -> 153 0.43567 37.96 % -2 -> 2
149 -> 153 0.19119 7.31 % -1 -> 2

9 Excited State: Singlet
3.6890 eV 336.09 nm 29754.cm^{-1} f=0.1106
150 -> 154 0.68882 94.89 % 0 -> 3

10 Excited State: Singlet
3.9117 eV 316.96 nm 31550.cm^{-1} f=0.1132
139 -> 153 0.12175 2.96 % -11 -> 2
140 -> 153 0.12494 3.12 % -10 -> 2
147 -> 153 0.14299 4.09 % -3 -> 2
148 -> 151 0.62575 78.31 % -2 -> 0
149 -> 152 0.11694 2.73 % -1 -> 1

11 Excited State: Singlet
3.9887 eV 310.84 nm 32171.cm^{-1} f=0.0288
134 -> 153 -0.13331 3.55 % -16 -> 2
135 -> 153 -0.23448 11.00 % -15 -> 2
137 -> 153 -0.15021 4.51 % -13 -> 2
138 -> 153 0.30769 18.93 % -12 -> 2
139 -> 153 0.20703 8.57 % -11 -> 2
140 -> 153 0.24648 12.15 % -10 -> 2
147 -> 151 -0.15236 4.64 % -3 -> 0
147 -> 153 0.25077 12.58 % -3 -> 2
148 -> 151 -0.11570 2.68 % -2 -> 0
148 -> 153 -0.24532 12.04 % -2 -> 2

12 Excited State: Singlet
4.0450 eV 306.52 nm 32624.cm^{-1} f=0.3055
147 -> 151 -0.37202 27.68 % -3 -> 0
148 -> 152 0.55574 61.77 % -2 -> 1
149 -> 152 0.13178 3.47 % -1 -> 1

13 Excited State: Singlet
4.1094 eV 301.71 nm 33144.cm^{-1} f=0.0128
135 -> 153 -0.15943 5.08 % -15 -> 2
138 -> 153 0.25909 13.43 % -12 -> 2
147 -> 151 0.42032 35.33 % -3 -> 0
147 -> 153 -0.13582 3.69 % -3 -> 2
148 -> 152 0.30523 18.63 % -2 -> 1
148 -> 153 0.15576 4.85 % -2 -> 2
150 -> 155 0.10015 2.01 % 0 -> 4

14 Excited State: Singlet
4.1505 eV 298.72 nm 33476.cm^{-1} f=0.0561
147 -> 152 -0.10724 2.30 % -3 -> 1
149 -> 154 0.50324 50.65 % -1 -> 3
150 -> 155 -0.44244 39.15 % 0 -> 4

15 Excited State: Singlet
4.1749 eV 296.97 nm 33673.cm^{-1} f=0.0566
134 -> 153 0.10097 2.04 % -16 -> 2
135 -> 153 0.14583 4.25 % -15 -> 2
138 -> 153 -0.23735 11.27 % -12 -> 2
140 -> 153 0.12113 2.93 % -10 -> 2
146 -> 151 -0.11327 2.57 % -4 -> 0
147 -> 151 0.27865 15.53 % -3 -> 0
147 -> 153 0.19736 7.79 % -3 -> 2
148 -> 151 -0.15237 4.64 % -2 -> 0
148 -> 152 0.22138 9.80 % -2 -> 1
148 -> 153 -0.26807 14.37 % -2 -> 2
149 -> 153 -0.12990 3.37 % -1 -> 2
149 -> 154 -0.18190 6.62 % -1 -> 3

Complex 3

HOMO is orbital 142 and LUMO is orbital 143
>>>> Singlet states <<<<

eV	nm	cm^{-1}	f
2.9313	422.97	23642.3	0.0030
3.2187	385.20	25960.5	0.0400
3.4044	364.19	27458.2	0.0192
3.4259	361.90	27631.9	0.1547
3.5160	352.63	28358.3	0.0585
3.7062	334.53	29892.7	0.1788
3.8272	323.95	30869.0	0.0299
3.8968	318.17	31429.7	0.2366
3.9099	317.10	31535.8	0.0950
3.9972	310.17	32240.4	0.0099
4.0584	305.50	32733.2	0.2247
4.1699	297.33	33632.7	0.0405
4.2295	293.14	34113.4	0.0117
4.2485	291.83	34266.5	0.1333
4.2787	289.77	34510.1	0.0006
4.4071	281.33	35545.4	0.0548
4.4390	279.30	35803.8	0.0340
4.4738	277.13	36084.1	0.1753
4.5986	269.61	37090.6	0.0354
4.6835	264.72	37775.8	0.0057
4.7068	263.41	37963.6	0.0362
4.7621	260.35	38409.8	0.0064
4.7983	258.39	38701.2	0.0081
4.8886	253.62	39429.1	0.0110
4.9003	253.02	39522.6	0.0864

1 Excited State: Singlet
2.9313 eV 422.97 nm 23642.cm^{-1} f=0.0030
134 -> 145 0.12843 3.30 % -8 -> 2
135 -> 145 -0.17922 6.42 % -7 -> 2
142 -> 143 0.16530 5.46 % 0 -> 0
142 -> 144 0.24450 11.96 % 0 -> 1
142 -> 145 0.57414 65.93 % 0 -> 2

2 Excited State: Singlet
3.2187 eV 385.20 nm 25961.cm^{-1} f=0.0400
137 -> 145 0.27435 15.05 % -5 -> 2
138 -> 145 0.23381 10.93 % -4 -> 2
139 -> 145 0.38199 29.18 % -3 -> 2
142 -> 143 0.25546 13.05 % 0 -> 0
142 -> 144 0.27337 14.95 % 0 -> 1
142 -> 145 -0.17559 6.17 % 0 -> 2

3 12 Excited State: Singlet
3.4044 eV 364.19 nm 27458.cm^{-1} f=0.0192
133 -> 145 -0.19428 7.55 % -9 -> 2
135 -> 145 -0.13477 3.63 % -7 -> 2
137 -> 145 -0.12710 3.23 % -5 -> 2
138 -> 145 -0.26155 13.68 % -4 -> 2
139 -> 145 0.13839 3.83 % -3 -> 2

140 -> 145 -0.25603 13.11 % -2 -> 2
141 -> 144 0.11685 2.73 % -1 -> 1
141 -> 145 0.38102 29.04 % -1 -> 2
142 -> 144 0.23265 10.83 % 0 -> 1
142 -> 145 -0.13079 3.42 % 0 -> 2

4 Excited State: Singlet
3.4259 eV 361.90 nm 27632.cm^{-1} f=0.1547
134 -> 145 -0.13613 3.71 % -8 -> 2
137 -> 145 -0.20120 8.10 % -5 -> 2
139 -> 145 -0.30510 18.62 % -3 -> 2
141 -> 143 -0.10857 2.36 % -1 -> 0
141 -> 145 -0.16648 5.54 % -1 -> 2
142 -> 143 0.32668 21.34 % 0 -> 0
142 -> 144 0.35381 25.04 % 0 -> 1
142 -> 145 -0.17505 6.13 % 0 -> 2

5 Excited State: Singlet
3.5160 eV 352.63 nm 28358.cm^{-1} f=0.0585
141 -> 145 0.14086 3.97 % -1 -> 2
142 -> 143 0.53302 56.82 % 0 -> 0
142 -> 144 -0.40461 32.74 % 0 -> 1

6 Excited State: Singlet
3.7062 eV 334.53 nm 29893.cm^{-1} f=0.1788
139 -> 145 -0.14362 4.13 % -3 -> 2
141 -> 143 0.63062 79.54 % -1 -> 0
141 -> 144 -0.20560 8.45 % -1 -> 1

7 Excited State: Singlet
3.8272 eV 323.95 nm 30869.cm^{-1} f=0.0299
133 -> 145 0.11206 2.51 % -9 -> 2
134 -> 145 0.10620 2.26 % -8 -> 2
135 -> 145 0.17981 6.47 % -7 -> 2
140 -> 143 0.15414 4.75 % -2 -> 0
140 -> 144 0.11235 2.52 % -2 -> 1
140 -> 145 0.42397 35.95 % -2 -> 2
141 -> 144 0.21306 9.08 % -1 -> 1
141 -> 145 0.38706 29.96 % -1 -> 2

8 Excited State: Singlet
3.8968 eV 318.17 nm 31430.cm^{-1} f=0.2366
131 -> 145 -0.18084 6.54 % -11 -> 2
134 -> 145 -0.16523 5.46 % -8 -> 2
135 -> 145 -0.19225 7.39 % -7 -> 2
138 -> 145 0.22815 10.41 % -4 -> 2
139 -> 145 -0.10179 2.07 % -3 -> 2
140 -> 143 -0.29642 17.57 % -2 -> 0
140 -> 144 -0.15687 4.92 % -2 -> 1
141 -> 143 0.11333 2.57 % -1 -> 0
141 -> 144 0.40465 32.75 % -1 -> 1

9 Excited State: Singlet
3.9099 eV 317.10 nm 31536.cm^{-1} f=0.0950
130 -> 145 0.11780 2.78 % -12 -> 2
131 -> 145 0.21646 9.37 % -11 -> 2
134 -> 145 0.26522 14.07 % -8 -> 2
135 -> 145 0.25004 12.50 % -7 -> 2
138 -> 145 -0.16068 5.16 % -4 -> 2
139 -> 145 0.11177 2.50 % -3 -> 2
140 -> 143 -0.26446 13.99 % -2 -> 0
140 -> 144 -0.16933 5.73 % -2 -> 1
141 -> 143 0.11906 2.84 % -1 -> 0
141 -> 144 0.24179 11.69 % -1 -> 1
141 -> 145 -0.24022 11.54 % -1 -> 2

10 Excited State: Singlet
3.9972 eV 310.17 nm 32240.cm^{-1} f=0.0099
140 -> 143 0.47349 44.84 % -2 -> 0
140 -> 145 -0.15124 4.57 % -2 -> 2
141 -> 143 0.16372 5.36 % -1 -> 0
141 -> 144 0.39114 30.60 % -1 -> 1
141 -> 145 -0.21115 8.92 % -1 -> 2

11 Excited State: Singlet
4.0584 eV 305.50 nm 32733.cm^{-1} f=0.2247
140 -> 143 -0.23929 11.45 % -2 -> 0
140 -> 144 0.60373 72.90 % -2 -> 1
142 -> 146 -0.11075 2.45 % 0 -> 3

12 Excited State: Singlet
4.1699 eV 297.33 nm 33633.cm^{-1} f=0.0405
137 -> 143 0.25831 13.34 % -5 -> 0
138 -> 143 0.21618 9.35 % -4 -> 0
139 -> 143 0.58892 69.37 % -3 -> 0

13 Excited State: Singlet
4.2295 eV 293.14 nm 34113.cm^{-1} f=0.0117
134 -> 145 -0.19670 7.74 % -8 -> 2
137 -> 145 -0.14148 4.00 % -5 -> 2
138 -> 145 -0.15225 4.64 % -4 -> 2
139 -> 144 0.19962 7.97 % -3 -> 1
139 -> 145 0.15786 4.98 % -3 -> 2
140 -> 145 0.22747 10.35 % -2 -> 2
142 -> 145 0.16858 5.68 % 0 -> 2
142 -> 146 0.46320 42.91 % 0 -> 3

14 Excited State: Singlet
4.2485 eV 291.83 nm 34267.cm^{-1} f=0.1333
134 -> 145 0.15817 5.00 % -8 -> 2
137 -> 145 0.10123 2.05 % -5 -> 2
138 -> 145 0.13680 3.74 % -4 -> 2
139 -> 144 -0.17469 6.10 % -3 -> 1
139 -> 145 -0.14765 4.36 % -3 -> 2
140 -> 144 0.13439 3.61 % -2 -> 1
140 -> 145 -0.22252 9.90 % -2 -> 2
142 -> 145 -0.11729 2.75 % 0 -> 2
142 -> 146 0.50291 50.58 % 0 -> 3

15 Excited State: Singlet
4.2787 eV 289.77 nm 34510.cm^{-1} f=0.0006
137 -> 144 0.28608 16.37 % -5 -> 1
138 -> 144 0.28013 15.69 % -4 -> 1
139 -> 144 0.49585 49.17 % -3 -> 1
139 -> 145 -0.19710 7.77 % -3 -> 2
140 -> 145 -0.10813 2.34 % -2 -> 2

16 Excited State: Singlet
4.4071 eV 281.33 nm 35545.cm^{-1} f=0.0548
135 -> 143 0.10354 2.14 % -7 -> 0
135 -> 145 0.11049 2.44 % -7 -> 2
137 -> 143 0.22478 10.11 % -5 -> 0
138 -> 143 0.27053 14.64 % -4 -> 0

139 -> 143 -0.17041 5.81 % -3 -> 0
140 -> 143 -0.10792 2.33 % -2 -> 0
141 -> 146 0.51030 52.08 % -1 -> 3

17 Excited State: Singlet
4.4390 eV 279.30 nm 35804.cm^{-1} f=0.0340
129 -> 145 0.11797 2.78 % -13 -> 2
134 -> 145 -0.22590 10.21 % -8 -> 2
135 -> 144 0.13735 3.77 % -7 -> 1
135 -> 145 0.44781 40.11 % -7 -> 2
138 -> 143 -0.11303 2.56 % -4 -> 0
138 -> 145 0.11662 2.72 % -4 -> 2
140 -> 145 -0.26374 13.91 % -2 -> 2
141 -> 145 0.14627 4.28 % -1 -> 2
141 -> 146 -0.10995 2.42 % -1 -> 3
142 -> 145 0.17627 6.21 % 0 -> 2

18 Excited State: Singlet
4.4738 eV 277.13 nm 36084.cm^{-1} f=0.1753
137 -> 143 -0.24185 11.70 % -5 -> 0
138 -> 143 -0.36493 26.63 % -4 -> 0
138 -> 144 0.11331 2.57 % -4 -> 1
139 -> 143 0.27997 15.68 % -3 -> 0
141 -> 143 -0.10411 2.17 % -1 -> 0
141 -> 146 0.41262 34.05 % -1 -> 3

19 Excited State: Singlet
4.5986 eV 269.61 nm 37091.cm^{-1} f=0.0354
137 -> 143 0.18845 7.10 % -5 -> 0
137 -> 144 0.39896 31.83 % -5 -> 1
138 -> 144 0.31715 20.12 % -4 -> 1
138 -> 145 -0.17428 6.07 % -4 -> 2
139 -> 144 -0.34580 23.92 % -3 -> 1

20 Excited State: Singlet
4.6835 eV 264.72 nm 37776.cm^{-1} f=0.0057
136 -> 143 0.16480 5.43 % -6 -> 0
136 -> 144 0.24889 12.39 % -6 -> 1
136 -> 145 -0.12351 3.05 % -6 -> 2
137 -> 143 -0.13935 3.88 % -5 -> 0
138 -> 143 0.12168 2.96 % -4 -> 0
140 -> 146 -0.12087 2.92 % -2 -> 3
140 -> 147 0.12669 3.21 % -2 -> 4
142 -> 147 0.53237 56.68 % 0 -> 4

21 Excited State: Singlet
4.7068 eV 263.41 nm 37964.cm^{-1} f=0.0362
137 -> 143 0.40407 32.65 % -5 -> 0
137 -> 144 0.10993 2.42 % -5 -> 1
138 -> 143 -0.37816 28.60 % -4 -> 0
138 -> 144 -0.28908 16.71 % -4 -> 1
139 -> 144 0.11769 2.77 % -3 -> 1
140 -> 146 -0.11248 2.53 % -2 -> 3
142 -> 147 0.14631 4.28 % 0 -> 4
142 -> 148 0.10292 2.12 % 0 -> 5

22 Excited State: Singlet
4.7621 eV 260.35 nm 38410.cm^{-1} f=0.0064
137 -> 143 -0.11180 2.50 % -5 -> 0
137 -> 144 0.26771 14.33 % -5 -> 1
138 -> 144 -0.24268 11.78 % -4 -> 1
138 -> 146 0.10224 2.09 % -4 -> 3
140 -> 146 0.54872 60.22 % -2 -> 3
142 -> 147 0.10004 2.00 % 0 -> 4

23 Excited State: Singlet
4.7983 eV 258.39 nm 38701.cm^{-1} f=0.0081
137 -> 143 0.24423 11.93 % -5 -> 0
137 -> 144 -0.35436 25.11 % -5 -> 1
138 -> 143 -0.16897 5.71 % -4 -> 0
138 -> 144 0.32044 20.54 % -4 -> 1
140 -> 146 0.36261 26.30 % -2 -> 3
142 -> 147 0.12260 3.01 % 0 -> 4

24 Excited State: Singlet
4.8886 eV 253.62 nm 39429.cm^{-1} f=0.0110
134 -> 143 -0.16608 5.52 % -8 -> 0
134 -> 144 -0.16019 5.13 % -8 -> 1
135 -> 143 -0.21427 9.18 % -7 -> 0
135 -> 144 -0.12957 3.36 % -7 -> 1
136 -> 143 0.37222 27.71 % -6 -> 0
136 -> 144 0.23856 11.38 % -6 -> 1
137 -> 145 -0.23550 11.09 % -5 -> 2
138 -> 145 0.17275 5.97 % -4 -> 2
142 -> 147 -0.25505 13.01 % 0 -> 4

25 Excited State: Singlet
4.9003 eV 253.02 nm 39523.cm^{-1} f=0.0864
136 -> 143 0.21665 9.39 % -6 -> 0
136 -> 144 0.17822 6.35 % -6 -> 1
137 -> 145 0.46237 42.76 % -5 -> 2
138 -> 145 -0.29672 17.61 % -4 -> 2
139 -> 145 -0.17054 5.82 % -3 -> 2
142 -> 147 -0.15508 4.81 % 0 -> 4

Table S6. Computed Singlet excited states at the M06/D95(d)SDD09/DCM level of theory for **1**, **2** and **3**. Structure optimizations at the same level of approximation were performed before TD-DFT computations. Number on the left indicates orbital involved in the excitation, coefficient of the Slater determinant defining the excitation, % contribution of the same determinant and occupied and virtual MOs which defines the Slater determinant (MOs on the last two columns are indicated numbering HOMO on the left of the arrow and LUMO on the right of the arrow as 0).

Complex 1

HOMO is orbital 118 and LUMO is orbital 119
>>>> Singlet states <<<<

eV	nm	cm-1	f
2.4176;	512.84;	19499.3;	0.0010
2.4730;	501.35;	19946.1;	0.0002
2.9986;	413.47;	24185.6;	0.0274
3.0355;	408.45;	24482.8;	0.0377
3.1819;	389.65;	25664.1;	0.0125
3.2316;	383.66;	26064.7;	0.2523
3.2668;	379.53;	26348.4;	0.0698
3.4354;	360.90;	27708.5;	0.0002
3.6116;	343.30;	29129.0;	0.0105
3.6761;	337.27;	29649.8;	0.0654
3.8209;	324.49;	30817.6;	0.2250
3.8784;	319.68;	31281.3;	0.3778
3.9693;	312.36;	32014.3;	0.0157
4.0775;	304.07;	32887.2;	0.1126
4.2527;	291.54;	34300.6;	0.0879

1 4 Excited State: Singlet-?
2.4176 eV 512.84 nm 19499.cm-1 f=0.0010
109 -> 119 -0.14315 4.10 % -9 -> 0
116 -> 119 -0.12073 2.92 % -2 -> 0
117 -> 119 0.50170 50.34 % -1 -> 0
117 -> 120 0.24580 12.08 % -1 -> 1
118 -> 119 0.30848 19.03 % 0 -> 0
118 -> 120 0.16155 5.22 % 0 -> 1

2 6 Excited State: Singlet-?
2.4730 eV 501.35 nm 19946.cm-1 f=0.0002
116 -> 119 0.10069 2.03 % -2 -> 0
117 -> 119 -0.28187 15.89 % -1 -> 0
117 -> 120 -0.13536 3.66 % -1 -> 1
118 -> 119 0.53380 56.99 % 0 -> 0
118 -> 120 0.28828 16.62 % 0 -> 1

3 10 Excited State: Singlet-?
2.9986 eV 413.47 nm 24186.cm-1 f=0.0274
113 -> 119 -0.16974 5.76 % -5 -> 0
113 -> 120 -0.10115 2.05 % -5 -> 1
114 -> 119 -0.17147 5.88 % -4 -> 0
118 -> 119 -0.28488 16.23 % 0 -> 0
118 -> 120 0.51666 53.39 % 0 -> 1
118 -> 121 -0.22358 10.00 % 0 -> 2

4 11 Excited State: Singlet-?
3.0355 eV 408.45 nm 24483.cm-1 f=0.0377
113 -> 119 0.28025 15.71 % -5 -> 0
113 -> 120 0.15685 4.92 % -5 -> 1
114 -> 119 0.29246 17.11 % -4 -> 0
114 -> 120 0.16943 5.74 % -4 -> 1
116 -> 119 -0.12332 3.04 % -2 -> 0
117 -> 119 -0.19889 7.91 % -1 -> 0
117 -> 120 0.25633 13.14 % -1 -> 1
118 -> 119 -0.15861 5.03 % 0 -> 0
118 -> 120 0.31642 20.02 % 0 -> 1
118 -> 121 0.13459 3.62 % 0 -> 2

5 15 Excited State: Singlet-?
3.1819 eV 389.65 nm 25664.cm-1 f=0.0125
110 -> 119 0.20166 8.13 % -8 -> 0
110 -> 120 0.12084 2.92 % -8 -> 1
111 -> 119 -0.21913 9.60 % -7 -> 0
111 -> 120 -0.12774 3.26 % -7 -> 1
114 -> 119 0.14884 4.43 % -4 -> 0
116 -> 119 0.45966 42.26 % -2 -> 0
116 -> 120 0.24157 11.67 % -2 -> 1
117 -> 119 0.14837 4.40 % -1 -> 0
118 -> 121 0.13356 3.57 % 0 -> 2

6 16 Excited State: Singlet-?
3.2316 eV 383.66 nm 26065.cm-1 f=0.2523
113 -> 119 -0.15733 4.95 % -5 -> 0
115 -> 119 0.12497 3.12 % -3 -> 0
117 -> 119 -0.23545 11.09 % -1 -> 0
117 -> 120 0.52983 56.14 % -1 -> 1
118 -> 120 -0.11264 2.54 % 0 -> 1
118 -> 121 -0.22954 10.54 % 0 -> 2

7 17 Excited State: Singlet-?
3.2668 eV 379.53 nm 26348.cm-1 f=0.0698
113 -> 119 -0.18477 6.83 % -5 -> 0
114 -> 119 -0.21626 9.35 % -4 -> 0
114 -> 120 -0.13581 3.69 % -4 -> 1
117 -> 120 0.13989 3.91 % -1 -> 1
118 -> 121 0.57383 65.86 % 0 -> 2
118 -> 122 -0.11709 2.74 % 0 -> 3

8 20 Excited State: Singlet-?
3.4354 eV 360.90 nm 27709.cm-1 f=0.0002
107 -> 119 -0.16053 5.15 % -11 -> 0
109 -> 119 -0.11753 2.76 % -9 -> 0
110 -> 119 0.40394 32.63 % -8 -> 0
110 -> 120 0.24213 11.73 % -8 -> 1
111 -> 119 0.29552 17.47 % -7 -> 0
111 -> 120 0.16579 5.50 % -7 -> 1
115 -> 119 -0.23787 11.32 % -3 -> 0
115 -> 120 -0.12470 3.11 % -3 -> 1

9 22 Excited State: Singlet-?
3.6116 eV 343.30 nm 29129.cm-1 f=0.0105
110 -> 119 -0.28082 15.77 % -8 -> 0
110 -> 120 -0.16505 5.45 % -8 -> 1
111 -> 119 0.19126 7.32 % -7 -> 0
111 -> 120 0.10691 2.29 % -7 -> 1

114 -> 119 -0.10442 2.18 % -4 -> 0
115 -> 119 -0.29059 16.89 % -3 -> 0
115 -> 120 -0.17040 5.81 % -3 -> 1
116 -> 119 0.35560 25.29 % -2 -> 0
117 -> 120 0.14960 4.48 % -1 -> 1
117 -> 121 -0.11529 2.66 % -1 -> 2

10 23 Excited State: Singlet-?
3.6761 eV 337.27 nm 29650.cm-1 f=0.0654
116 -> 120 0.11106 2.47 % -2 -> 1
116 -> 121 -0.19075 7.28 % -2 -> 2
117 -> 121 0.64348 82.81 % -1 -> 2

11 25 Excited State: Singlet-?
3.8209 eV 324.49 nm 30818.cm-1 f=0.2250
117 -> 121 0.11113 2.47 % -1 -> 2
118 -> 121 0.12398 3.07 % 0 -> 2
118 -> 122 0.66788 89.21 % 0 -> 3

12 27 Excited State: Singlet-?
3.8784 eV 319.68 nm 31281.cm-1 f=0.3778
115 -> 119 -0.21105 8.91 % -3 -> 0
116 -> 119 -0.27136 14.73 % -2 -> 0
116 -> 120 0.58424 68.27 % -2 -> 1

13 28 Excited State: Singlet-?
3.9693 eV 312.36 nm 32014.cm-1 f=0.0157
111 -> 119 0.33672 22.68 % -7 -> 0
111 -> 120 0.17542 6.15 % -7 -> 1
113 -> 119 0.10751 2.31 % -5 -> 0
115 -> 119 0.47968 46.02 % -3 -> 0
116 -> 120 0.21185 8.98 % -2 -> 1
117 -> 119 0.13518 3.65 % -1 -> 0

14 29 Excited State: Singlet-?
4.0775 eV 304.07 nm 32887.cm-1 f=0.1126
109 -> 119 0.10000 2.00 % -9 -> 0
111 -> 119 0.13381 3.58 % -7 -> 0
115 -> 119 -0.14650 4.29 % -3 -> 0
115 -> 120 0.62099 77.13 % -3 -> 1

15 30 Excited State: Singlet-?
4.2527 eV 291.54 nm 34301.cm-1 f=0.0879
115 -> 121 -0.11960 2.86 % -3 -> 2
116 -> 121 0.62343 77.73 % -2 -> 2
117 -> 121 0.17020 5.79 % -1 -> 2
117 -> 122 -0.17547 6.16 % -1 -> 3
118 -> 122 -0.10209 2.08 % 0 -> 3

Complex 2

HOMO is orbital 150 and LUMO is orbital 151
>>>> Singlet states <<<<

eV	nm	cm^{-1}	f
2.3679;	523.61;	19098.2;	0.0013
2.5819;	480.21;	20824.2;	0.0023
2.9946;	414.03;	24152.8;	0.0579
3.0432;	407.41;	24545.3;	0.2556
3.1357;	395.39;	25291.5;	0.0298
3.1745;	390.57;	25603.6;	0.0797
3.2978;	375.96;	26598.6;	0.1846
3.4288;	361.59;	27655.6;	0.0013
3.4787;	356.41;	28057.6;	0.0805
3.5610;	348.18;	28720.8;	0.0326
3.6235;	342.17;	29225.2;	0.0856
3.8334;	323.43;	30918.6;	0.0850
3.8947;	318.34;	31413.0;	0.1474
3.9712;	312.21;	32029.7;	0.2737
4.0931;	302.91;	33013.1;	0.0199
4.1147;	301.32;	33187.3;	0.2362
4.1607;	297.99;	33558.2;	0.1911
4.2700;	290.36;	34440.0;	0.0009
4.2890;	289.08;	34592.5;	0.0458
4.3029;	288.14;	34705.4;	0.0086
4.3381;	285.80;	34989.5;	0.0336
4.3682;	283.83;	35232.4;	0.0283
4.4014;	281.69;	35500.0;	0.0066
4.4307;	279.83;	35736.0;	0.0087
4.4583;	278.10;	35958.3;	0.0101

1 3 Excited State: Singlet-A
2.3679 eV 523.61 nm 19098.cm-1 f=0.0013
137 -> 151 0.13845 3.83 % -13 -> 0
140 -> 151 -0.12231 2.99 % -10 -> 0
148 -> 151 0.12429 3.09 % -2 -> 0
149 -> 151 0.47810 45.72 % -1 -> 0
150 -> 151 -0.42721 36.50 % 0 -> 0

2 7 Excited State: Singlet-A
2.5819 eV 480.21 nm 20824.cm-1 f=0.0023
148 -> 151 0.14330 4.11 % -2 -> 0
149 -> 151 0.38367 29.44 % -1 -> 0
150 -> 151 0.53011 56.20 % 0 -> 0
150 -> 152 0.11776 2.77 % 0 -> 1

3 9 Excited State: Singlet-A
2.9946 eV 414.03 nm 24153.cm-1 f=0.0579
141 -> 151 0.25776 13.29 % -9 -> 0
144 -> 151 -0.20997 8.82 % -6 -> 0
145 -> 151 -0.33566 22.53 % -5 -> 0
148 -> 151 0.10124 2.05 % -2 -> 0
149 -> 151 -0.10765 2.32 % -1 -> 0
149 -> 152 0.20050 8.04 % -1 -> 1
150 -> 152 0.17071 5.83 % 0 -> 1
150 -> 153 0.36529 26.69 % 0 -> 2

4 12 Excited State: Singlet-A
3.0432 eV 407.41 nm 24545.cm-1 f=0.2556
145 -> 151 0.12179 2.97 % -5 -> 0
148 -> 151 -0.11810 2.79 % -2 -> 0
149 -> 152 -0.15262 4.66 % -1 -> 1
150 -> 151 -0.12852 3.30 % 0 -> 0
150 -> 152 0.61962 76.79 % 0 -> 1

5 14 Excited State: Singlet-A
3.1357 eV 395.39 nm 25291.cm-1 f=0.0298
138 -> 151 -0.14689 4.32 % -12 -> 0

139 -> 151 0.17460 6.10 % -11 -> 0
140 -> 151 0.14511 4.21 % -10 -> 0
147 -> 151 0.14237 4.05 % -3 -> 0
148 -> 151 0.40251 32.40 % -2 -> 0
149 -> 151 -0.18117 6.56 % -1 -> 0
150 -> 151 -0.10334 2.14 % 0 -> 0
150 -> 152 0.14664 4.30 % 0 -> 1
150 -> 153 -0.36485 26.62 % 0 -> 2

6 17 Excited State: Singlet-A
3.1745 eV 390.57 nm 25604.cm-1 f=0.0797
138 -> 151 -0.10544 2.22 % -12 -> 0
139 -> 151 0.13635 3.72 % -11 -> 0
141 -> 151 -0.13749 3.78 % -9 -> 0
144 -> 151 0.23299 10.86 % -6 -> 0
145 -> 151 0.27914 15.58 % -5 -> 0
148 -> 151 0.23388 10.94 % -2 -> 0
150 -> 152 -0.11069 2.45 % 0 -> 1
150 -> 153 0.43865 38.48 % 0 -> 2

7 19 Excited State: Singlet-A
3.2978 eV 375.96 nm 26599.cm-1 f=0.1846
144 -> 151 0.10029 2.01 % -6 -> 0
145 -> 151 0.11390 2.59 % -5 -> 0
147 -> 151 -0.11580 2.68 % -3 -> 0
149 -> 151 -0.11001 2.42 % -1 -> 0
149 -> 152 0.57986 67.25 % -1 -> 1
149 -> 153 -0.18322 6.71 % -1 -> 2
150 -> 152 0.14812 4.39 % 0 -> 1
150 -> 153 -0.13075 3.42 % 0 -> 2

8 21 Excited State: Singlet-A
3.4288 eV 361.59 nm 27656.cm-1 f=0.0013
135 -> 151 -0.18414 6.78 % -15 -> 0
137 -> 151 0.15744 4.96 % -13 -> 0
138 -> 151 0.47327 44.80 % -12 -> 0
138 -> 152 0.13174 3.47 % -12 -> 1
139 -> 151 0.18645 6.95 % -11 -> 0
140 -> 151 0.23347 10.90 % -10 -> 0
141 -> 151 -0.10353 2.14 % -9 -> 0
147 -> 151 0.21092 8.90 % -3 -> 0
148 -> 151 -0.11113 2.47 % -2 -> 0

9 24 Excited State: Singlet-A
3.4787 eV 356.41 nm 28058.cm-1 f=0.0805
138 -> 151 -0.12946 3.35 % -12 -> 0
148 -> 153 0.15321 4.69 % -2 -> 2
149 -> 152 0.21139 8.94 % -1 -> 1
149 -> 153 0.61044 74.53 % -1 -> 2

10 25 Excited State: Singlet-A
3.5610 eV 348.18 nm 28721.cm-1 f=0.0326
138 -> 151 0.29342 17.22 % -12 -> 0
140 -> 151 -0.22964 10.55 % -10 -> 0
146 -> 151 -0.15115 4.57 % -4 -> 0
147 -> 151 -0.30374 18.45 % -3 -> 0
148 -> 151 0.33185 22.02 % -2 -> 0
149 -> 151 -0.10353 2.14 % -1 -> 0
149 -> 152 -0.10125 2.05 % -1 -> 1
149 -> 153 0.17155 5.89 % -1 -> 2

11 26 Excited State: Singlet-A
3.6235 eV 342.17 nm 29225.cm-1 f=0.0856
150 -> 154 0.68801 94.67 % 0 -> 3

12 30 Excited State: Singlet-A
3.8334 eV 323.43 nm 30919.cm-1 f=0.0850
139 -> 151 -0.17589 6.19 % -11 -> 0
140 -> 151 -0.30614 18.74 % -10 -> 0
146 -> 151 -0.11152 2.49 % -4 -> 0
147 -> 151 0.50855 51.72 % -3 -> 0
148 -> 152 -0.18180 6.61 % -2 -> 1
149 -> 151 -0.13260 3.52 % -1 -> 0

13 31 Excited State: Singlet-A
3.8947 eV 318.34 nm 31413.cm-1 f=0.1474
140 -> 151 -0.11777 2.77 % -10 -> 0
147 -> 152 0.17672 6.25 % -3 -> 1
148 -> 151 -0.11289 2.55 % -2 -> 0
148 -> 152 0.60365 72.88 % -2 -> 1
149 -> 153 -0.10959 2.40 % -1 -> 2

14 34 Excited State: Singlet-A
3.9712 eV 312.21 nm 32030.cm-1 f=0.2737
147 -> 152 -0.31451 19.78 % -3 -> 1
148 -> 153 0.59142 69.96 % -2 -> 2
149 -> 153 -0.15847 5.02 % -1 -> 2

15 40 Excited State: Singlet-A
4.0931 eV 302.91 nm 33013.cm-1 f=0.0199
147 -> 152 0.47388 44.91 % -3 -> 1
147 -> 153 -0.17367 6.03 % -3 -> 2
148 -> 152 -0.22542 10.16 % -2 -> 1
148 -> 153 0.25557 13.06 % -2 -> 2
149 -> 154 -0.18307 6.70 % -1 -> 3
150 -> 155 -0.15776 4.98 % 0 -> 4

16 41 Excited State: Singlet-A
4.1147 eV 301.32 nm 33187.cm-1 f=0.2362
147 -> 152 0.19223 7.39 % -3 -> 1
148 -> 153 0.13691 3.75 % -2 -> 2
149 -> 154 0.17790 6.33 % -1 -> 3
150 -> 155 0.62673 78.56 % 0 -> 4

17 42 Excited State: Singlet-A
4.1607 eV 297.99 nm 33558.cm-1 f=0.1911
147 -> 152 0.13486 3.64 % -3 -> 1
148 -> 154 0.14537 4.23 % -2 -> 3
149 -> 154 0.60482 73.16 % -1 -> 3
150 -> 155 -0.24324 11.83 % 0 -> 4

18 43 Excited State: Singlet-A
4.2700 eV   290.36 nm   34440.cm-1   f=0.0009
137 -> 151   0.27033 14.62 % -13 -> 0
138 -> 151   -0.12367   3.06 % -12 -> 0
139 -> 151   0.15386   4.73 % -11 -> 0
141 -> 151   0.21618   9.35 % -9 -> 0
144 -> 151   0.32616 21.28 % -6 -> 0
145 -> 151   -0.13509   3.65 % -5 -> 0
146 -> 151   -0.28646 16.41 % -4 -> 0
147 -> 152   -0.11996   2.88 % -3 -> 1
148 -> 151   -0.22871 10.46 % -2 -> 0
149 -> 154   0.12220   2.99 % -1 -> 3

19 44 Excited State: Singlet-A
4.2890 eV   289.08 nm   34593.cm-1   f=0.0458
146 -> 151   0.17992   6.47 % -4 -> 0
147 -> 152   0.14214   4.04 % -3 -> 1
147 -> 153   0.59265 70.25 % -3 -> 2
149 -> 154   -0.10540   2.22 % -1 -> 3

20 45 Excited State: Singlet-A
4.3029 eV   288.14 nm   34705.cm-1   f=0.0086
137 -> 151   0.17426   6.07 % -13 -> 0
140 -> 151   -0.17131   5.87 % -10 -> 0
141 -> 151   0.11416   2.61 % -9 -> 0
144 -> 151   0.10999   2.42 % -6 -> 0
146 -> 151   0.53718 57.71 % -4 -> 0
147 -> 153   -0.24719 12.22 % -3 -> 2

21 46 Excited State: Singlet-A
4.3381 eV   285.80 nm   34990.cm-1   f=0.0336
141 -> 152   -0.22452 10.08 % -9 -> 1
144 -> 151   -0.10578   2.24 % -6 -> 0
144 -> 152   0.25424 12.93 % -6 -> 1
145 -> 151   -0.14045   3.95 % -5 -> 0
145 -> 152   0.49474 48.95 % -5 -> 1
146 -> 152   0.23591 11.13 % -4 -> 1

22 47 Excited State: Singlet-A
4.3682 eV   283.83 nm   35232.cm-1   f=0.0283
145 -> 153   0.11433   2.61 % -5 -> 2
146 -> 152   -0.19211   7.38 % -4 -> 1
150 -> 156   0.50194 50.39 %   0 -> 5
150 -> 158   0.36632 26.84 %   0 -> 7

23 48 Excited State: Singlet-A
4.4014 eV   281.69 nm   35500.cm-1   f=0.0066
150 -> 156   0.12764   3.26 %   0 -> 5
150 -> 157   0.67118 90.10 %   0 -> 6

24 49 Excited State: Singlet-A
4.4307 eV   279.83 nm   35736.cm-1   f=0.0087
145 -> 153   -0.10446   2.18 % -5 -> 2
146 -> 152   0.22343   9.98 % -4 -> 1
150 -> 156   0.44676 39.92 %   0 -> 5
150 -> 157   -0.10496   2.20 %   0 -> 6
150 -> 158   -0.40906 33.47 %   0 -> 7

25 50 Excited State: Singlet-A
4.4583 eV   278.10 nm   35958.cm-1   f=0.0101
140 -> 152   0.11736   2.75 % -10 -> 1
141 -> 152   0.16193   5.24 % -9 -> 1
144 -> 152   -0.14783   4.37 % -6 -> 1
145 -> 152   -0.11415   2.61 % -5 -> 1
146 -> 152   0.46565 43.37 % -4 -> 1
146 -> 153   -0.17920   6.42 % -4 -> 2
150 -> 158   0.27703 15.35 %   0 -> 7

Complex 3

HOMO is orbital 142 and LUMO is orbital 143

>>>> Singlet states <<<<

eV	nm	cm-1	f
2.4746;	501.02;	19959.3;	0.0013
2.9428;	421.31;	23735.5;	0.0004
2.9639;	418.31;	23905.7;	0.0018
3.2861;	377.30;	26504.1;	0.0261
3.3468;	370.45;	26994.2;	0.1358
3.3747;	367.39;	27219.0;	0.0399
3.5233;	351.90;	28417.2;	0.0626
3.6242;	342.10;	29231.2;	0.1143
3.6853;	336.43;	29723.9;	0.0935
3.8831;	319.29;	31319.5;	0.3939
3.9007;	317.85;	31461.4;	0.0076
4.0140;	308.88;	32375.0;	0.0510
4.0617;	305.25;	32760.0;	0.1746
4.2426;	292.24;	34218.5;	0.0450
4.2492;	291.79;	34271.2;	0.0461

1   1 Excited State: Singlet-A
2.4746 eV   501.02 nm   19959.cm-1   f=0.0013
134 -> 143   0.13763   3.79 % -8 -> 0
135 -> 143   -0.18572   6.90 % -7 -> 0
139 -> 143   -0.10650   2.27 % -3 -> 0
142 -> 143   0.64477 83.15 %   0 -> 0

2   2 Excited State: Singlet-A
2.9428 eV   421.31 nm   23735.cm-1   f=0.0004
133 -> 143   -0.19385   7.52 % -9 -> 0
135 -> 143   -0.16488   5.44 % -7 -> 0
138 -> 143   -0.22480 10.11 % -4 -> 0
139 -> 143   0.26167 13.69 % -3 -> 0
140 -> 143   -0.26423 13.96 % -2 -> 0
141 -> 143   0.47301 44.75 % -1 -> 0

3   3 Excited State: Singlet-A
2.9639 eV   418.31 nm   23906.cm-1   f=0.0018
136 -> 143   0.11848   2.81 % -6 -> 0

137 -> 143 0.52430 54.98 % -5 -> 0
138 -> 143 0.14008 3.92 % -4 -> 0
139 -> 143 0.33765 22.80 % -3 -> 0
142 -> 145 0.16141 5.21 % 0 -> 2

4 4 Excited State: Singlet-A
3.2861 eV 377.30 nm 26504.cm-1 f=0.0261
134 -> 143 0.24680 12.18 % -8 -> 0
135 -> 143 0.15638 4.89 % -7 -> 0
140 -> 143 0.41526 34.49 % -2 -> 0
141 -> 143 0.36042 25.98 % -1 -> 0
142 -> 144 -0.12383 3.07 % 0 -> 1
142 -> 145 -0.24771 12.27 % 0 -> 2

5 5 Excited State: Singlet-A
3.3468 eV 370.45 nm 26994.cm-1 f=0.1358
134 -> 143 -0.14790 4.37 % -8 -> 0
137 -> 143 -0.13046 3.40 % -5 -> 0
139 -> 143 -0.11906 2.84 % -3 -> 0
140 -> 143 0.22270 9.92 % -2 -> 0
141 -> 143 0.24966 12.47 % -1 -> 0
142 -> 144 0.28007 15.69 % 0 -> 1
142 -> 145 0.47169 44.50 % 0 -> 2

6 6 Excited State: Singlet-A
3.3747 eV 367.39 nm 27219.cm-1 f=0.0399
131 -> 143 0.27779 15.43 % -11 -> 0
134 -> 143 0.40204 32.33 % -8 -> 0
135 -> 143 0.29481 17.38 % -7 -> 0
138 -> 143 -0.24479 11.98 % -4 -> 0
141 -> 143 -0.17391 6.05 % -1 -> 0
142 -> 144 0.10835 2.35 % 0 -> 1
142 -> 145 0.19138 7.33 % 0 -> 2

7 7 Excited State: Singlet-A
3.5233 eV 351.90 nm 28417.cm-1 f=0.0626
142 -> 144 0.60704 73.70 % 0 -> 1
142 -> 145 -0.34373 23.63 % 0 -> 2

8 8 Excited State: Singlet-A
3.6242 eV 342.10 nm 29231.cm-1 f=0.1143
134 -> 143 0.13891 3.86 % -8 -> 0
138 -> 143 0.10429 2.18 % -4 -> 0
139 -> 143 -0.20735 8.60 % -3 -> 0
140 -> 143 -0.18035 6.51 % -2 -> 0
141 -> 144 0.55620 61.87 % -1 -> 1
141 -> 145 -0.13222 3.50 % -1 -> 2
142 -> 143 -0.11144 2.48 % 0 -> 0

9 9 Excited State: Singlet-A
3.6853 eV 336.43 nm 29724.cm-1 f=0.0935
134 -> 143 -0.22438 10.07 % -8 -> 0
137 -> 143 -0.18877 7.13 % -5 -> 0
138 -> 143 -0.23894 11.42 % -4 -> 0
139 -> 143 0.25762 13.27 % -3 -> 0
140 -> 143 0.24414 11.92 % -2 -> 0
141 -> 144 0.34668 24.04 % -1 -> 1
141 -> 145 -0.13014 3.39 % -1 -> 2
142 -> 143 0.17916 6.42 % 0 -> 0

10 10 Excited State: Singlet-A
3.8831 eV 319.29 nm 31319.cm-1 f=0.3939
135 -> 143 -0.17451 6.09 % -7 -> 0
140 -> 143 0.11682 2.73 % -2 -> 0
140 -> 144 0.40241 32.39 % -2 -> 1
140 -> 145 0.33664 22.67 % -2 -> 2
141 -> 144 -0.11878 2.82 % -1 -> 1
141 -> 145 -0.33247 22.11 % -1 -> 2

11 11 Excited State: Singlet-A
3.9007 eV 317.85 nm 31461.cm-1 f=0.0076
134 -> 143 -0.16584 5.50 % -8 -> 0
135 -> 143 0.48929 47.88 % -7 -> 0
138 -> 143 0.13968 3.90 % -4 -> 0
140 -> 143 -0.27057 14.64 % -2 -> 0
140 -> 144 0.12201 2.98 % -2 -> 1
140 -> 145 0.15672 4.91 % -2 -> 2
141 -> 143 0.14274 4.07 % -1 -> 0
141 -> 145 -0.16543 5.47 % -1 -> 2
142 -> 143 0.12420 3.09 % 0 -> 0

12 12 Excited State: Singlet-A
4.0140 eV 308.88 nm 32375.cm-1 f=0.0510
140 -> 144 0.48681 47.40 % -2 -> 1
141 -> 145 0.47158 44.48 % -1 -> 2

13 13 Excited State: Singlet-A
4.0617 eV 305.25 nm 32760.cm-1 f=0.1746
140 -> 144 -0.20178 8.14 % -2 -> 1
140 -> 145 0.55789 62.25 % -2 -> 2
141 -> 144 0.11460 2.63 % -1 -> 1
141 -> 145 0.30282 18.34 % -1 -> 2

14 14 Excited State: Singlet-A
4.2426 eV 292.24 nm 34218.cm-1 f=0.0450
137 -> 144 -0.17751 6.30 % -5 -> 1
139 -> 144 -0.35385 25.04 % -3 -> 1
141 -> 146 0.10237 2.10 % -1 -> 3
142 -> 146 0.53697 57.67 % 0 -> 3

15 15 Excited State: Singlet-A
4.2492 eV 291.79 nm 34271.cm-1 f=0.0461
137 -> 144 0.25077 12.58 % -5 -> 1
139 -> 144 0.47883 45.86 % -3 -> 1
142 -> 146 0.40232 32.37 % 0 -> 3

Table S7. Computed Cartesian coordinates at the MPW1PW91/SD09/D95(d)/DCM level of approximation. Neutral and charged compounds were computed and reported here. These structures were used for the evaluation of the oxidation and reduction potentials (Figure 4 in the paper) as energy difference between charged and neutral compounds (see "Computational Details" for more information).

Complex 1 –Neutral Singlet State

Pd	0.043398	0.452395	0.006825
N	-0.050347	2.447208	-0.364896
N	-1.810711	0.845579	0.703056
N	0.079498	-1.592942	0.029929
O	1.975726	0.474670	-0.516783
O	6.522266	-0.599818	0.362126
C	0.960694	3.170548	-0.867772
H	1.908442	2.648066	-0.949909
C	0.807065	4.491061	-1.261809
H	1.654001	5.040620	-1.658311
C	-0.465528	5.071709	-1.140047
H	-0.632472	6.100085	-1.450103
C	-1.513662	4.327348	-0.625621
H	-2.502889	4.758240	-0.533417
C	-1.294790	2.992425	-0.221865
C	-2.255126	2.122415	0.377801
C	-3.535396	2.340055	0.924887
C	-3.840421	1.158243	1.618442
H	-4.738673	0.963472	2.194676
C	-2.757343	0.269393	1.477621
C	-2.631397	-1.064767	2.138460
H	-1.583750	-1.328993	2.304896
H	-3.135461	-1.038581	3.109987
H	-3.091251	-1.866098	1.550348
C	-4.395217	3.566515	0.852211
H	-3.907890	4.439815	1.303964
H	-4.651864	3.832977	-0.180351
H	-5.333326	3.401697	1.390254
C	-1.075606	-2.361945	-0.303893
C	-1.961171	-1.875696	-1.270762
H	-1.767076	-0.911958	-1.733186
C	-3.081451	-2.620226	-1.640145
H	-3.754043	-2.219647	-2.395666
C	-3.355274	-3.866123	-1.060469
C	-2.466040	-4.336908	-0.083017
H	-2.661593	-5.288177	0.407840
C	-1.343752	-3.602011	0.294228
H	-0.705533	-3.979351	1.089271
C	-4.551672	-4.679041	-1.479865
H	-5.340400	-4.044307	-1.894046
H	-4.276111	-5.408106	-2.251886
C	1.197292	-2.252334	0.184701
H	1.137907	-3.341603	0.160530
C	2.515828	-1.724054	0.306538
C	2.850330	-0.391782	-0.094757
C	4.216986	-0.010186	-0.089579
H	4.449058	1.000560	-0.406697
C	5.206824	-0.898932	0.314559
C	4.877331	-2.222862	0.707325
H	5.670467	-2.900374	1.007476
C	3.559479	-2.611829	0.677339
H	3.299508	-3.631698	0.953871
C	6.928416	0.703241	-0.030283
H	6.668197	0.902262	-1.076325
H	6.478876	1.469510	0.611728
H	8.012993	0.727065	0.083295
H	-4.968967	-5.239162	-0.637335

Complex 1 –Anionic Doublet State

Pd	-0.103338	0.433198	-0.053571
N	-0.503447	2.540379	-0.497702
N	-2.155614	0.763615	0.679508
N	0.430446	-1.688912	-0.190406
O	2.073821	0.791435	-0.313524
O	6.722059	0.151255	0.535865
C	0.380802	3.355898	-1.082096
H	1.367121	2.925191	-1.240726
C	0.087720	4.662539	-1.460563
H	0.843885	5.283348	-1.931096
C	-1.211553	5.130341	-1.212079
H	-1.498527	6.140997	-1.494342
C	-2.135448	4.294199	-0.603751
H	-3.141931	4.645814	-0.414218
C	-1.765933	2.977544	-0.236328
C	-2.637013	2.035366	0.421326
C	-3.939593	2.188296	0.958174
C	-4.229323	0.953938	1.566370
H	-5.140773	0.696137	2.098196
C	-3.110619	0.115276	1.373476
C	-2.953919	-1.294160	1.854978
H	-1.954483	-1.461830	2.271720
H	-3.692295	-1.515081	2.633292
H	-3.088318	-2.023727	1.047268
C	-4.854634	3.380046	0.948140
H	-4.404332	4.260488	1.425291
H	-5.149957	3.678426	-0.066664
H	-5.774451	3.150594	1.496235
C	-0.582913	-2.660914	-0.372357
C	-1.541790	-2.461902	-1.378061
H	-1.481939	-1.557953	-1.979668
C	-2.547028	-3.399906	-1.598370
H	-3.272860	-3.222765	-2.390380
C	-2.647020	-4.563350	-0.817578
C	-1.697767	-4.746814	0.195008
H	-1.757194	-5.627411	0.832300
C	-0.679397	-3.815549	0.418312
H	0.021765	-3.972021	1.234908
C	-3.749409	-5.562949	-1.057191
H	-4.735983	-5.112860	-0.895399
H	-3.731024	-5.935223	-2.088083
C	1.641987	-2.154255	-0.044958
H	1.764686	-3.244326	-0.063478
C	2.876159	-1.441293	0.105347
C	3.032175	-0.010148	-0.039169
C	4.359136	0.517065	0.115112
H	4.463313	1.592240	0.010754
C	5.448029	-0.296957	0.380073
C	5.290585	-1.702870	0.502255
H	6.158166	-2.324307	0.702755
C	4.027784	-2.232709	0.356201
H	3.899655	-3.311210	0.443712
C	6.949784	1.545053	0.424810
H	6.677120	1.917836	-0.570094
H	6.390868	2.104268	1.184981
H	8.019790	1.691808	0.583541
H	-3.660323	-6.423356	-0.387548

Complex 1 –Cationic Doublet State

Pd	0.084564	0.432304	0.042055
N	-0.022829	2.446196	-0.268073
N	-1.820900	0.814380	0.666323
N	0.125454	-1.593948	0.013797
O	2.007295	0.511073	-0.404931
O	6.568052	-0.623521	0.263813
C	0.994752	3.195620	-0.685293
H	1.959406	2.700015	-0.725121
C	0.827162	4.532262	-1.059426
H	1.687559	5.104986	-1.388366
C	-0.448448	5.091890	-1.009084
H	-0.613880	6.123235	-1.304981
C	-1.514143	4.309557	-0.571208
H	-2.514406	4.719498	-0.524516
C	-1.276797	2.981275	-0.193311
C	-2.257882	2.079508	0.342434
C	-3.621984	2.286845	0.803658
C	-3.938878	1.129631	1.459165
H	-4.862677	0.902292	1.977307
C	-2.795784	0.243425	1.394592
C	-2.687611	-1.045864	2.109363
H	-1.649667	-1.358680	2.221755
H	-3.151089	-0.949305	3.096368
H	-3.236027	-1.829442	1.571672
C	-4.478867	3.498413	0.644094
H	-4.043742	4.367437	1.150458
H	-4.619212	3.756611	-0.411166
H	-5.463754	3.317863	1.079688
C	-1.049004	-2.348277	-0.287848
C	-1.935217	-1.877654	-1.261857
H	-1.718579	-0.946458	-1.778610
C	-3.080754	-2.607191	-1.582366
H	-3.753361	-2.222912	-2.346013
C	-3.374425	-3.821966	-0.948717
C	-2.477553	-4.279853	0.028635
H	-2.684547	-5.209621	0.554181
C	-1.331717	-3.559403	0.359524
H	-0.683171	-3.922948	1.152712
C	-4.596536	-4.621156	-1.314946
H	-5.362126	-3.992122	-1.777698
H	-4.341589	-5.411974	-2.030807
C	1.245523	-2.262541	0.121111
H	1.172826	-3.349663	0.070058
C	2.566176	-1.747449	0.228858
C	2.893304	-0.390491	-0.075115
C	4.254085	-0.002440	-0.075846
H	4.477385	1.030178	-0.319736
C	5.255402	-0.919182	0.230139
C	4.935507	-2.270480	0.528913
H	5.737219	-2.966302	0.754526
C	3.619585	-2.660048	0.506944
H	3.366911	-3.697479	0.714956
C	6.969135	0.706146	-0.039148
H	6.681870	0.983177	-1.059776
H	6.539889	1.420653	0.672281
H	8.056115	0.717979	0.046837
H	-5.033644	-5.106424	-0.436871

Complex 2 – Neutral singlet state

Pd	-0.585589	-0.473766	-0.105779
N	0.020840	-2.388983	-0.409564
N	1.341509	-0.331971	0.467920
N	-1.101018	1.490733	-0.248776
O	-2.477262	-1.012095	-0.459767
O	-7.095070	-0.922315	0.663278
C	-0.788781	-3.377491	-0.814408
H	-1.850890	-3.160372	-0.759651
C	-0.302407	-4.586949	-1.289005
H	-0.992462	-5.365414	-1.596304
C	1.087349	-4.748328	-1.379928
H	1.510171	-5.666974	-1.777924
C	1.926392	-3.725097	-0.965717
H	3.002374	-3.824993	-1.040866
C	1.376676	-2.537769	-0.448612
C	2.105312	-1.415845	0.083834
C	3.461328	-1.177250	0.396106
C	3.485239	0.094549	0.995625
H	4.350567	0.562431	1.449708
C	2.164010	0.570947	1.062184
C	-0.162730	2.457205	-0.722243
C	0.726665	2.098928	-1.741343
H	0.714930	1.082824	-2.126293
C	1.603154	3.042096	-2.275155
H	2.277044	2.742411	-3.075236
C	1.625581	4.363945	-1.808836
C	0.736190	4.705779	-0.781087
H	0.739220	5.718473	-0.382995
C	-0.142514	3.770696	-0.236836
H	-0.787220	4.059868	0.588624
C	2.596210	5.370852	-2.367846
H	2.801892	5.182248	-3.426042
H	2.214477	6.391486	-2.269132
C	-2.331498	1.890411	-0.081607
H	-2.528985	2.955995	-0.209533
C	-3.481054	1.085938	0.182052
C	-3.509253	-0.324208	-0.059381
C	-4.742743	-1.008319	0.090839
H	-4.742547	-2.074943	-0.105101
C	-5.893886	-0.333166	0.481902
C	-5.869551	1.066488	0.714298
H	-6.784202	1.572136	1.007451
C	-4.685196	1.743615	0.543681
H	-4.661282	2.820599	0.698068
C	-7.197346	-2.320843	0.439053
H	-6.955012	-2.575799	-0.599162
H	-6.542559	-2.880685	1.116804
H	-8.236578	-2.582690	0.642183
H	3.554785	5.326097	-1.836062
C	1.722381	1.731982	1.841532
C	2.561189	2.851910	1.981355
C	0.513673	1.711511	2.559685
C	2.200369	3.919269	2.802555
H	3.497648	2.890270	1.430112
C	0.148796	2.782898	3.375451
H	-0.129767	0.837761	2.496745
C	0.989091	3.893850	3.502617
H	2.863028	4.776969	2.892695
H	-0.788103	2.743309	3.926622
H	0.707968	4.725119	4.144729
C	4.649517	-2.040106	0.244875
C	4.706242	-3.327276	0.806501
C	5.791321	-1.551665	-0.412908
C	5.858906	-4.107856	0.697184
H	3.846598	-3.710854	1.351254
C	6.946428	-2.329676	-0.521247
H	5.765358	-0.556135	-0.850071
C	6.984734	-3.614160	0.029774
H	5.881410	-5.099303	1.143472
H	7.816413	-1.933361	-1.039662
H	7.882776	-4.221216	-0.054527

Complex 2 –Anionic Doublet State

Pd	-0.473405	-0.281070	-0.379917
N	0.221414	-2.260450	-0.936970
N	1.561012	-0.396960	0.468231
N	-1.274521	1.766726	-0.578571
O	-2.604594	-0.808451	-0.743776
O	-6.823072	-1.073288	1.477037
C	-0.501757	-3.091101	-1.697830
H	-1.562972	-2.859876	-1.754261
C	0.046346	-4.172063	-2.380846
H	-0.585023	-4.819277	-2.981685
C	1.429926	-4.370389	-2.279695
H	1.914139	-5.180659	-2.819623
C	2.185808	-3.514165	-1.490611
H	3.259404	-3.640868	-1.416653
C	1.556251	-2.462462	-0.793940
C	2.240564	-1.522286	0.078319
C	3.553353	-1.521454	0.622549
C	3.646410	-0.325274	1.356317
H	4.486025	-0.025342	1.973820
C	2.399408	0.322357	1.259624
C	-0.511623	2.832639	-1.096619
C	0.889066	2.722661	-1.152008
H	1.352184	1.818983	-0.766997
C	1.666372	3.746953	-1.681784
H	2.749173	3.632232	-1.696604
C	1.089653	4.918050	-2.201027
C	-0.306384	5.009402	-2.178849
H	-0.793620	5.885969	-2.602732
C	-1.098391	3.987855	-1.646678
H	-2.180113	4.079017	-1.702464
C	1.945559	6.014046	-2.782061
H	2.531742	5.652630	-3.635372
H	1.336651	6.855205	-3.126475
C	-2.430981	2.060754	-0.042558
H	-2.648671	3.117011	0.160458
C	-3.478384	1.159908	0.331677
C	-3.521312	-0.234992	-0.054059
C	-4.675647	-0.985168	0.347335
H	-4.696576	-2.030591	0.056399
C	-5.709465	-0.408986	1.069194
C	-5.666058	0.962679	1.433916
H	-6.491400	1.393989	1.992470
C	-4.569351	1.705734	1.055469
H	-4.532305	2.761475	1.322544
C	-6.930614	-2.447255	1.148786
H	-6.938119	-2.600698	0.062734
H	-6.112693	-3.029800	1.589940
H	-7.880400	-2.786004	1.566795
H	2.658165	6.395917	-2.041545
C	2.005059	1.542110	1.970595
C	2.972487	2.488635	2.361596
C	0.664806	1.788199	2.321011
C	2.614714	3.634949	3.071500
H	4.013875	2.330795	2.090376
C	0.305553	2.937959	3.024833
H	-0.093962	1.063513	2.035727
C	1.276340	3.870049	3.407667
H	3.381497	4.352184	3.356893
H	-0.738194	3.103233	3.284219
H	0.995907	4.764529	3.958981
C	4.619011	-2.539434	0.592901
C	4.369286	-3.889370	0.903134
C	5.948835	-2.164411	0.326513
C	5.402174	-4.828052	0.928298
H	3.354985	-4.199943	1.142691
C	6.985707	-3.100047	0.356321
H	6.164795	-1.126455	0.083265
C	6.718399	-4.440193	0.653317
H	5.180523	-5.864196	1.174702
H	8.003818	-2.782711	0.141351
H	7.523466	-5.170880	0.674864

Complex 2 –Cationic Doublet State

Pd	-0.634554	-0.462983	-0.041817
N	-0.004546	-2.388585	-0.273575
N	1.327388	-0.295121	0.474278
N	-1.157051	1.479215	-0.253307
O	-2.496774	-1.041701	-0.356700
O	-7.161999	-0.934583	0.512687
C	-0.800817	-3.403695	-0.604575
H	-1.866174	-3.213375	-0.522596
C	-0.294351	-4.625783	-1.052834
H	-0.979832	-5.428511	-1.302402
C	1.084846	-4.769454	-1.193663
H	1.510589	-5.694127	-1.571206
C	1.918265	-3.707748	-0.847395
H	2.992350	-3.786127	-0.959170
C	1.350282	-2.525726	-0.361110
C	2.075470	-1.366711	0.108976
C	3.486952	-1.103665	0.343650
C	3.529216	0.170817	0.855434
H	4.410972	0.676986	1.225483
C	2.176469	0.631497	1.007789
C	-0.197044	2.436115	-0.703736
C	0.706954	2.077213	-1.708367
H	0.675071	1.074491	-2.126229
C	1.623431	3.009885	-2.195924
H	2.307945	2.711881	-2.987097
C	1.667516	4.318298	-1.697688
C	0.754770	4.662151	-0.689321
H	0.768276	5.667293	-0.273225
C	-0.163500	3.740255	-0.192584
H	-0.834198	4.031078	0.611346
C	2.672973	5.317474	-2.205169
H	3.075015	5.020898	-3.177951
H	2.228730	6.312705	-2.306340
C	-2.395743	1.879733	-0.136936
H	-2.585171	2.942258	-0.295268
C	-3.548958	1.082069	0.106219
C	-3.555668	-0.338794	-0.051359
C	-4.783823	-1.030283	0.075033
H	-4.764984	-2.106129	-0.058468
C	-5.960571	-0.345851	0.364047
C	-5.959330	1.065921	0.517775
H	-6.892903	1.575570	0.733467
C	-4.776323	1.746912	0.371478
H	-4.769699	2.830541	0.467092
C	-7.246727	-2.345119	0.358339
H	-6.944801	-2.651992	-0.649515
H	-6.629890	-2.862012	1.102145
H	-8.294919	-2.601142	0.516053
H	3.517538	5.408129	-1.511187
C	1.774391	1.756321	1.816328
C	2.668497	2.830897	2.021551
C	0.540864	1.748646	2.504756
C	2.326945	3.875550	2.871409
H	3.618155	2.855232	1.494844
C	0.214598	2.786796	3.368346
H	-0.135442	0.907632	2.385225
C	1.101736	3.856029	3.550623
H	3.013922	4.705189	3.011969
H	-0.727747	2.763920	3.908497
H	0.842839	4.667340	4.225765

C	4.655403	-1.979252	0.154756
C	4.736065	-3.237245	0.775597
C	5.748877	-1.518010	-0.596313
C	5.881384	-4.021373	0.635009
H	3.914652	-3.591114	1.393901
C	6.890280	-2.307788	-0.741759
H	5.694955	-0.545254	-1.078692
C	6.959675	-3.562193	-0.128396
H	5.934752	-4.988052	1.128697
H	7.725166	-1.943708	-1.334644
H	7.849619	-4.176002	-0.239885

Complex 3 –Neutral Singlet State

N	0.944579	-1.525837	0.042571
C	2.282850	-1.340571	-0.169066
C	2.977968	-2.523129	0.146790
C	2.012361	-3.453823	0.556378
C	0.782192	-2.805233	0.478009
C	2.676297	-0.056566	-0.714910
C	3.956568	0.261799	-1.187645
C	4.179354	1.484803	-1.806018
C	3.111449	2.370744	-1.964061
C	1.875032	2.007363	-1.450002
N	1.662058	0.846321	-0.813479
Pd	-0.131418	0.203534	-0.078234
O	-1.808086	-0.689698	0.454568
C	-2.994742	-0.302981	0.079542
C	-4.037738	-1.252329	0.068776
N	-1.085204	2.001354	0.014073
C	-2.379692	2.110487	-0.126926
C	-3.321682	1.052604	-0.260086
C	4.431674	-2.817295	0.092972
H	4.763036	-0.452236	-1.093355
H	1.024150	2.668300	-1.549223
H	3.223248	3.324400	-2.468336
H	5.168638	1.733495	-2.179498
H	2.183911	-4.475176	0.865302
C	-0.514858	-3.468405	0.804229
C	-4.660185	1.382068	-0.576814
C	-5.340568	-0.899860	-0.278403
H	-3.812726	-2.280656	0.331408
C	-5.666676	0.438950	-0.612134
H	-2.798233	3.117287	-0.080490
C	-0.376828	3.196054	0.346305
C	0.532152	3.178411	1.411469
C	1.225664	4.335658	1.757433
C	1.040653	5.537722	1.056479
C	0.134333	5.537147	-0.011513
C	-0.569150	4.384724	-0.368280
H	0.684875	2.257584	1.968711
H	1.923521	4.303072	2.591549
C	1.788118	6.783843	1.451573
H	-0.020749	6.447738	-0.586066
H	-1.243581	4.406723	-1.221198
H	1.680895	7.570106	0.699404
H	1.412636	7.178104	2.403284
H	2.855909	6.580899	1.584614
H	-4.898993	2.418843	-0.805679
H	-6.677115	0.729737	-0.874368
O	-6.242608	-1.904889	-0.265713
C	-7.587896	-1.614532	-0.618737
H	-8.125364	-2.560957	-0.546484
H	-8.036122	-0.892141	0.072855
H	-7.659572	-1.236546	-1.644902
F	-1.115130	-2.979650	1.905725
F	-1.409569	-3.415120	-0.199978
F	-0.299037	-4.781080	1.050110

F	4.705719	-4.031499	0.600099
F	5.179474	-1.928178	0.789761
F	4.934984	-2.808427	-1.167620

Complex 3 –Anionic Doublet State

N	1.135262	-1.554056	0.609351
C	2.272331	-1.530234	-0.137810
C	3.094438	-2.631808	0.208217
C	2.409766	-3.342442	1.208329
C	1.221624	-2.641412	1.413670
C	2.441129	-0.434706	-1.091853
C	3.395790	-0.425695	-2.122955
C	3.454740	0.652571	-2.998229
C	2.552545	1.708487	-2.840611
C	1.632406	1.624702	-1.800512
N	1.574351	0.596077	-0.946688
Pd	-0.119237	0.214522	0.434869
O	-2.152895	-0.496721	1.024258
C	-3.129049	-0.151626	0.269361
C	-4.191242	-1.067164	0.002431
N	-1.156871	2.145062	0.487512
C	-2.317181	2.225744	-0.114801
C	-3.248332	1.165148	-0.336527
C	4.435964	-3.017960	-0.283128
H	4.069762	-1.262219	-2.253074
H	0.907511	2.417902	-1.636246
H	2.554190	2.570020	-3.500850
H	4.188250	0.663020	-3.800297
H	2.741361	-4.236951	1.717996
C	0.149303	-3.025287	2.367987
C	-4.383845	1.461452	-1.125882
C	-5.283146	-0.736425	-0.788965
H	-4.134289	-2.060639	0.439657
C	-5.394918	0.551559	-1.373726
H	-2.637274	3.199907	-0.503351
C	-0.496174	3.342454	0.830297
C	0.883456	3.302367	1.104521
C	1.582178	4.449222	1.467886
C	0.938637	5.689764	1.599897
C	-0.441733	5.721729	1.367945
C	-1.152998	4.576899	1.001096
H	1.395383	2.345439	1.032186
H	2.650685	4.375673	1.663732
C	1.700875	6.922993	2.010587
H	-0.984260	6.656917	1.495256
H	-2.230980	4.651659	0.888822
H	1.056704	7.807104	2.013466
H	2.123006	6.812854	3.016565
H	2.537967	7.117769	1.330282
H	-4.463044	2.458903	-1.557484
H	-6.243288	0.829205	-1.988773
O	-6.217010	-1.712396	-0.955264
C	-7.344354	-1.430940	-1.765976
H	-7.948005	-2.340513	-1.768314
H	-7.941604	-0.606707	-1.357312
H	-7.052671	-1.192323	-2.796005
F	-0.220817	-2.021741	3.190774
F	-0.981696	-3.457599	1.764223
F	0.560421	-4.041853	3.161849
F	4.952902	-4.038166	0.430983
F	5.346192	-2.013106	-0.223719
F	4.448558	-3.433368	-1.580880

Complex 3 – Cationic Doublet State

N	0.928725	-1.479129	0.150142
C	2.248333	-1.323167	-0.177960
C	2.953033	-2.490978	0.157740
C	2.016752	-3.379429	0.707511
C	0.790346	-2.723725	0.688057
C	2.602444	-0.070935	-0.810788
C	3.841153	0.231264	-1.388726
C	4.011147	1.432192	-2.064823
C	2.932713	2.313169	-2.176072
C	1.737536	1.969788	-1.563572
N	1.581716	0.829613	-0.872111
Pd	-0.140604	0.229462	0.004776
O	-1.844876	-0.624902	0.571161
C	-2.991944	-0.287576	0.122855
C	-4.028833	-1.255374	0.118973
N	-1.082549	2.036790	0.076553
C	-2.360888	2.152257	-0.166221
C	-3.286374	1.075858	-0.326736
C	4.399067	-2.801662	0.003760
H	4.655126	-0.478065	-1.327600
H	0.877925	2.623449	-1.631917
H	3.005746	3.247345	-2.721693
H	4.968763	1.668414	-2.519378
H	2.205062	-4.379472	1.071575
C	-0.483168	-3.328421	1.177492
C	-4.590734	1.368146	-0.746309
C	-5.294075	-0.940377	-0.346728
H	-3.802047	-2.258295	0.462486
C	-5.577953	0.394171	-0.789449
H	-2.796325	3.150894	-0.166759
C	-0.359090	3.208785	0.414326
C	0.650424	3.111782	1.386224
C	1.350147	4.245917	1.775270
C	1.091342	5.499460	1.193526
C	0.100792	5.574013	0.201738
C	-0.624428	4.451220	-0.185265
H	0.860709	2.151670	1.849107
H	2.113858	4.158065	2.544079
C	1.857066	6.716779	1.626644
H	-0.100983	6.524394	-0.285534
H	-1.357991	4.540069	-0.981827
H	1.641937	7.576598	0.987823
H	1.596343	6.985997	2.656914
H	2.935906	6.530574	1.606530
H	-4.837396	2.380989	-1.052080
H	-6.567143	0.659297	-1.145102
O	-6.197629	-1.919850	-0.351694
C	-7.514784	-1.666873	-0.845910
H	-8.042891	-2.615693	-0.758990
H	-8.023244	-0.911308	-0.239386
H	-7.488309	-1.360202	-1.896165
F	-1.011324	-2.681239	2.234181
F	-1.439304	-3.384238	0.227519
F	-0.263002	-4.595403	1.575616
F	4.702707	-3.991395	0.544971
F	5.197802	-1.884875	0.597307
F	4.794044	-2.852780	-1.291287

Figure S20. Emission spectrum of **1** dissolved in a 5:5:2 mixture of cyclohexane, ethanol and 2-methylbutane, at 77 K (a); time-resolved luminescence intensity of **1** at 77 K (b).

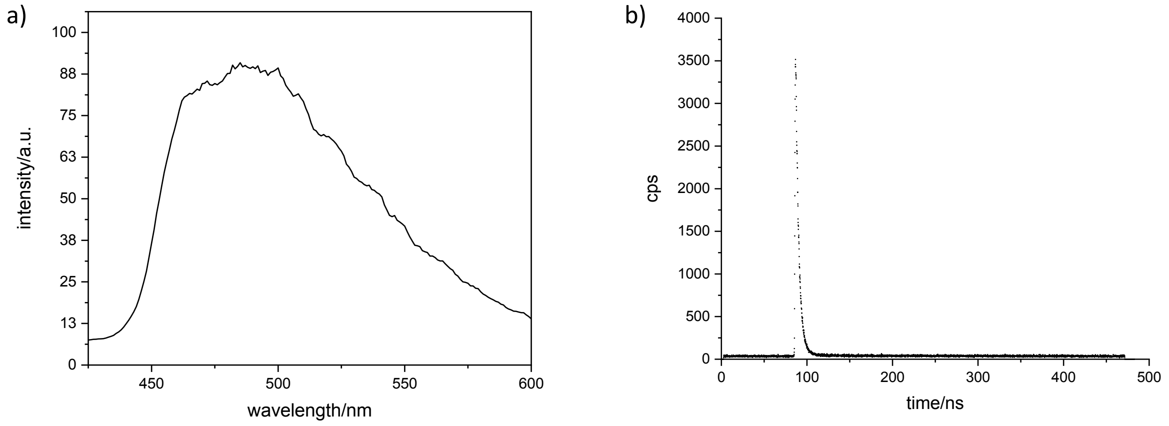

Effect of packing on the internal torsion angles

Structure optimizations of **1** ground and T_1 states were repeated after placing the molecule in a cage of five nearest neighbour molecules and modelling the system with the ONIOM approach (see "Computational Details" for more information). Using Figure S22 as reference, the ONIOM computations were performed by modelling the molecule b in the stack at the MPW1PW91/ SDD09/D95d level, molecules a and b along the stack plus three additional molecules surrounding molecule b in the crystal were modelled at the semiempirical PM6 level. Figure S21 (top part) shows the cluster of molecules used in the ONIOM investigation and the way in which molecule b is surrounded.

Figure S21. Results from the structure optimization of **1** ground and T_1 excited states. The experimental (crystal), the ONIOM and the in-vacuum structures are overlapped and sketched from two different viewpoints. (a) and (b) are two views of the ground state optimizations. (c) and (d) are two views of the T_1 optimized structures. The cluster of molecules of the ONIOM computations is visible in the top of the figure. Molecule b (the same visible in the stack of Figure 8) in the center of the cluster was modelled at the DFT level.

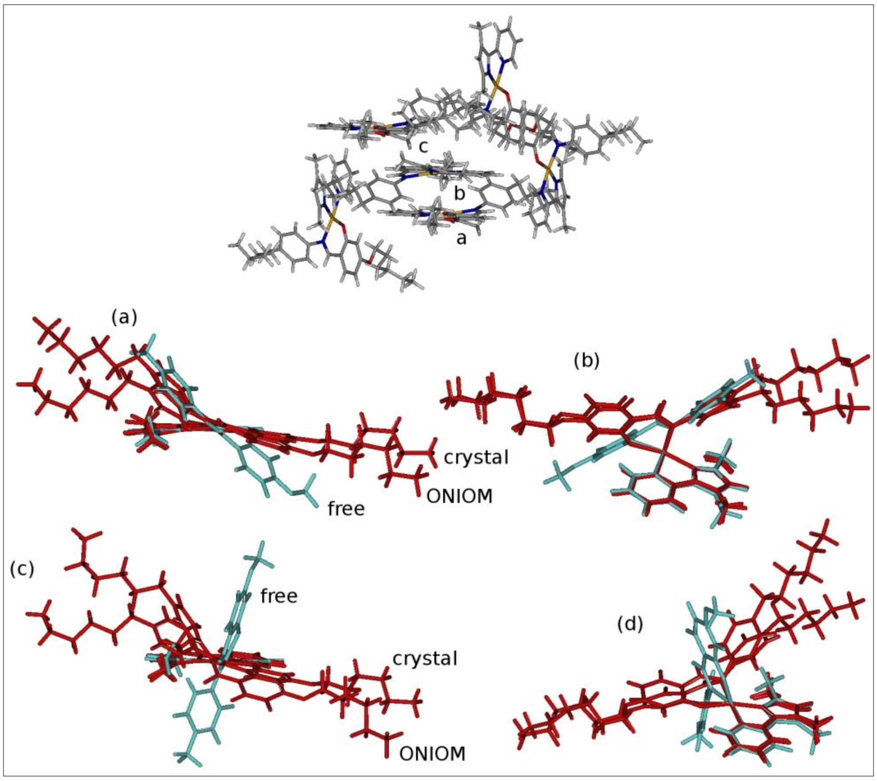

Geometry optimization was performed on all the internal degree of freedom, both in the low (PM6) and in the high-level layers (DFT) of ONIOM, apart from all the Pd-Pd distances, which were frozen to the experimental value for preventing drifts of the molecules which are impossible in solid phase.

Figures S21a and S21b show the obtained ground state structure from two different viewpoints (the structure of molecule b, the one computed at high level, referred to as "ONIOM structure" in the following) which is overlapped to the experimental structure (labelled as "crystal" in the figure). The two structures are in turn overlapped to the one computed *in vacuo*. The ONIOM structure appears very similar to the experimental one, with higher planarity in comparison to the structure computed *in vacuo*. The average unsigned variation of the torsion angles (dihedral angles among four connected atoms) is 4 degrees, if we exclude from this average the lateral aliphatic chains on the O^N ligand. The larger distortion is confined to one of the O^N phenyl, which points outside the cage of molecules in the ONIOM computations and is consequently expected to be relatively free to distort in comparison to the real crystal.

In the case of the T_1 state, (Figure S21c and S21d), the difference between the *in vacuo* and crystal structures is more evident. Conversely, the T_1 ONIOM structure is comparable to the crystal one, similarly to what it is observed in the ground state. Also, in this case, the values of torsion angles remain substantially unchanged. We can then conclude that the crystal packing substantially hampers any significant modification of the values of molecular torsion angles both in the ground and in the excited states. The results allow to assume that even in the case of the other compounds packing constraints can be mimicked in a single molecule excited state calculation, freezing the torsional variable while allowing all other variables free to relax.

ONIOM computations: optimized structures

Table S8. Cartesian coordinates (Å) obtained after geometry optimization with ONIOM. Compound **1** is reported. The high level layer was set to MPW1PW91/SDD09/D95(d), as all the DFT computations in this paper, and it was specified in the coordinates below with "H" at the end of the lines. The low level layer was treated at the PM6 levels ("L" label in the end of the line).

Singlet: the high level layer was computed as an unrestricted **singlet** state. The low level layer was computed as a restricted singlet state.

Pd	-1.292894	-1.633627	-0.454578	H
N	0.250552	-2.411711	-1.519540	H
N	-1.747616	-3.612836	-0.334591	H
N	-3.008629	-0.814943	0.293513	H
O	-0.402977	0.161435	-0.508931	H
O	0.750925	4.527712	1.098372	H
C	1.265464	-1.668461	-2.011631	H
H	1.235027	-0.616283	-1.741397	H
C	2.252749	-2.225511	-2.824501	H
H	3.066416	-1.609259	-3.186106	H
C	2.140298	-3.583962	-3.177493	H
H	2.880604	-4.039337	-3.826708	H
C	1.072191	-4.340695	-2.702586	H
H	0.960116	-5.376697	-2.984921	H
C	0.111897	-3.743878	-1.851937	H
C	-0.991637	-4.397760	-1.222395	H
C	-1.415969	-5.743331	-1.148297	H
C	-2.411170	-5.767129	-0.149292	H
H	-2.944718	-6.641783	0.209473	H
C	-2.587303	-4.455025	0.344884	H
C	-3.488798	-4.069620	1.471550	H
H	-3.145810	-3.156930	1.977434	H
H	-3.521263	-4.876749	2.217428	H
H	-4.517408	-3.894607	1.140301	H
C	-0.939632	-6.927192	-1.939723	H
H	0.093964	-7.218456	-1.683938	H
H	-0.972256	-6.737036	-3.019446	H
H	-1.577391	-7.793169	-1.742244	H
C	-4.289606	-1.424450	0.039459	H
C	-4.476896	-2.167965	-1.134605	H
H	-3.635922	-2.336111	-1.794406	H
C	-5.736966	-2.688886	-1.450432	H
H	-5.855721	-3.257027	-2.367096	H
C	-6.848798	-2.478657	-0.618176	H
C	-6.640264	-1.759546	0.575200	H
H	-7.477549	-1.577884	1.246484	H
C	-5.383928	-1.251764	0.909429	H
H	-5.256621	-0.748057	1.864135	H
C	-8.228743	-2.969791	-1.000858	H
H	-8.169619	-3.544403	-1.931753	H
H	-8.865343	-2.095617	-1.209936	H
C	-8.923321	-3.815793	0.082045	H
H	-8.307703	-4.695757	0.309796	H
H	-8.989463	-3.230300	1.010480	H
C	-10.337696	-4.255366	-0.322774	H
H	-10.278323	-4.889570	-1.216213	H
H	-10.922823	-3.360465	-0.607162	H
C	-11.065634	-4.993901	0.811220	H
H	-11.154450	-4.309442	1.667256	H
H	-10.436171	-5.835049	1.156588	H
C	-12.464485	-5.517762	0.441786	H
H	-12.958477	-5.879522	1.352897	H
H	-13.074874	-4.682832	0.070736	H
C	-12.454086	-6.647634	-0.599459	H
H	-12.044407	-6.311892	-1.557717	H
H	-13.466987	-7.020448	-0.785952	H
H	-11.845049	-7.490623	-0.252517	H
C	-3.006479	0.367123	0.881620	H
H	-3.961255	0.739011	1.253391	H
C	-1.929375	1.294479	1.008767	H
C	-0.726030	1.209782	0.239649	H
C	0.171727	2.306934	0.250421	H
H	1.020186	2.244204	-0.423396	H
C	-0.084940	3.432176	1.028031	H
C	-1.242527	3.497774	1.846448	H
H	-1.419037	4.375270	2.460898	H
C	-2.145915	2.456887	1.807343	H
H	-3.050239	2.520372	2.411233	H
C	1.803847	4.686746	0.098440	H
H	1.344062	4.672899	-0.904616	H
H	2.513668	3.851018	0.175621	H
C	2.497727	6.013969	0.368906	H
H	3.311139	6.116329	-0.363751	H
H	2.957735	5.975251	1.361427	H

C	1.551362	7.219013	0.258533	H
H	0.782258	7.137144	1.042984	H
H	1.021001	7.159413	-0.704342	H
C	2.259411	8.579886	0.363890	H
H	3.028781	8.655545	-0.418709	H
H	1.529076	9.373627	0.154077	H
C	2.900692	8.857317	1.734244	H
H	3.681944	8.115048	1.939728	H
H	2.139147	8.732107	2.516633	H
C	3.504357	10.265956	1.824333	H
H	4.283475	10.408257	1.066331	H
H	3.952462	10.449930	2.806843	H
H	2.736259	11.030466	1.657904	H
Pd	-0.114017	1.161749	-3.567014	L
N	-1.986772	1.444109	-2.873548	L
N	0.003326	3.178684	-3.320961	L
N	1.779081	0.790270	-4.268163	L
O	-0.550549	-0.780878	-3.965084	L
O	-2.249444	-2.540113	-8.067735	L
C	-2.933692	0.480280	-2.764814	L
H	-2.588551	-0.552075	-2.982347	L
C	-4.249910	0.788565	-2.390904	L
H	-5.010895	0.012157	-2.359234	L
C	-4.568078	2.109619	-2.055813	L
H	-5.592367	2.364382	-1.767230	L
C	-3.582876	3.093939	-2.101226	L
H	-3.795097	4.123283	-1.806362	L
C	-2.282783	2.758747	-2.540506	L
C	-1.199404	3.676801	-2.748276	L
C	-1.085131	5.088171	-2.580623	L
C	0.166942	5.455321	-3.092675	L
H	0.579978	6.435861	-3.145882	L
C	0.799711	4.257767	-3.557631	L
C	2.110357	4.197373	-4.248047	L
H	2.137433	3.395329	-4.999902	L
H	2.322407	5.141158	-4.768607	L
H	2.932906	4.018931	-3.539280	L
C	-2.064670	6.004758	-1.955067	L
H	-2.084982	6.980953	-2.456492	L
H	-3.087740	5.598515	-1.963758	L
H	-1.797781	6.183678	-0.900088	L
C	2.917700	1.282700	-3.543126	L
C	2.704077	1.636857	-2.197641	L
H	1.699050	1.510458	-1.741943	L
C	3.751240	2.167855	-1.444293	L
H	3.577828	2.442685	-0.401616	L
C	5.018391	2.358910	-2.016146	L
C	5.224509	1.999428	-3.357943	L
H	6.213930	2.131515	-3.808515	L
C	4.190642	1.458867	-4.119191	L
H	4.379028	1.182388	-5.155519	L
C	6.146837	2.954353	-1.220473	L
H	6.154721	2.546913	-0.189336	L
H	7.123241	2.646369	-1.655927	L
C	6.039469	4.487283	-1.190684	L
H	5.182094	4.786399	-0.557446	L
H	5.809040	4.862037	-2.208238	L
C	7.329823	5.143533	-0.681780	L
H	7.615041	4.719434	0.299668	L
H	8.168140	4.903558	-1.364892	L
C	7.165074	6.665152	-0.563936	L
H	6.670196	7.058681	-1.474363	L
H	6.480955	6.901893	0.273733	L
C	8.497702	7.402789	-0.371460	L
H	8.310893	8.494397	-0.427150	L
H	9.178429	7.172305	-1.214655	L
C	9.187730	7.074517	0.949805	L
H	9.623544	6.059149	0.932684	L
H	10.002694	7.775844	1.157546	L
H	8.490553	7.117970	1.793387	L
C	1.920921	-0.014044	-5.298381	L
H	2.908848	-0.283610	-5.717292	L
C	0.782942	-0.618025	-5.950713	L
C	-0.420446	-0.963944	-5.227315	L
C	-1.473948	-1.639917	-5.931830	L
H	-2.351120	-1.907971	-5.361012	L
C	-1.323303	-1.914622	-7.274736	L
C	-0.146403	-1.567760	-8.003089	L

H	-0.089805	-1.805514	-9.057857	L
C	0.873808	-0.939448	-7.324832	L
H	1.785820	-0.673401	-7.863899	L
C	-3.517230	-2.954642	-7.461230	L
H	-3.297972	-3.636750	-6.623096	L
H	-4.029834	-2.048854	-7.094374	L
C	-4.261182	-3.621527	-8.614431	L
H	-5.352339	-3.520784	-8.460134	L
H	-4.032797	-3.069339	-9.553258	L
C	-3.877539	-5.096903	-8.780770	L
H	-2.778999	-5.175011	-8.915161	L
H	-4.106227	-5.651691	-7.850241	L
C	-4.601824	-5.761964	-9.958307	L
H	-5.688314	-5.559619	-9.899949	L
H	-4.499233	-6.861676	-9.873274	L
C	-4.057992	-5.301746	-11.318221	L
H	-4.152438	-4.201441	-11.408215	L
H	-2.972214	-5.513105	-11.378573	L
C	-4.783461	-5.980378	-12.477907	L
H	-5.856208	-5.757532	-12.469265	L
H	-4.386580	-5.644586	-13.442509	L
H	-4.674476	-7.070067	-12.440236	L
Pd	-2.214790	3.730899	5.187136	L
N	-4.080272	4.406136	5.627295	L
N	-1.942003	5.670194	4.585663	L
N	-0.363311	2.896102	4.963440	L
O	-2.843927	1.946804	5.885082	L
O	-4.330910	-2.141640	3.933903	L
C	-5.075985	3.662918	6.173693	L
H	-4.846547	2.588718	6.309436	L
C	-6.300450	4.226652	6.547785	L
H	-7.083217	3.610177	6.975808	L
C	-6.488631	5.605348	6.363414	L
H	-7.434419	6.065628	6.649833	L
C	-5.471694	6.379940	5.820953	L
H	-5.590585	7.456667	5.679353	L
C	-4.246106	5.772711	5.446866	L
C	-3.116033	6.431602	4.879545	L
C	-2.897731	7.789648	4.486138	L
C	-1.610585	7.853403	3.949413	L
H	-1.114034	8.709254	3.552468	L
C	-1.055479	6.531262	4.018393	L
C	0.283836	6.164873	3.509949	L
H	0.384928	5.087556	3.299111	L
H	0.494271	6.670989	2.545079	L
H	1.076054	6.442605	4.218142	L
C	-3.841025	8.926031	4.594179	L
H	-4.300012	9.142846	3.618116	L
H	-4.656625	8.732008	5.308329	L
H	-3.326282	9.840183	4.921658	L
C	0.780831	3.665705	5.371725	L
C	0.561224	4.609405	6.396032	L
H	-0.443967	4.715922	6.833845	L
C	1.614507	5.412133	6.828928	L
H	1.441643	6.146946	7.614590	L
C	2.890238	5.290127	6.251851	L
C	3.099807	4.347747	5.233684	L
H	4.084072	4.249886	4.777570	L
C	2.054890	3.534399	4.792268	L
H	2.226854	2.821011	3.987389	L
C	4.016225	6.178511	6.704057	L
H	3.887992	6.459582	7.768972	L
H	4.980882	5.633267	6.662122	L
C	4.088110	7.443675	5.833262	L
H	3.126429	7.991151	5.892662	L
H	4.202934	7.158481	4.769151	L
C	5.242520	8.357219	6.262866	L
H	5.116618	8.654223	7.322150	L
H	6.200912	7.805623	6.217127	L
C	5.320019	9.609911	5.379728	L
H	5.438328	9.310028	4.319611	L
H	4.362533	10.164274	5.429958	L
C	6.478498	10.536875	5.775276	L
H	6.655410	11.263522	4.957197	L
H	7.415278	9.952702	5.864137	L
C	6.217139	11.293203	7.075976	L
H	6.074461	10.607239	7.918816	L
H	7.054180	11.953852	7.326437	L

H	5.316227	11.913522	7.006572 L
C	-0.233711	1.629419	4.638505 L
H	0.755660	1.144134	4.535058 L
C	-1.365364	0.753622	4.431210 L
C	-2.615523	0.944108	5.121369 L
C	-3.637165	-0.056885	5.006352 L
H	-4.535307	0.080713	5.588540 L
C	-3.423560	-1.140759	4.177877 L
C	-2.220904	-1.311278	3.440311 L
H	-2.119700	-2.161056	2.759612 L
C	-1.217427	-0.376801	3.593092 L
H	-0.285145	-0.505642	3.034115 L
C	-5.590150	-2.142942	4.683752 L
H	-5.350469	-2.458237	5.712688 L
H	-6.006866	-1.121449	4.689536 L
C	-6.471751	-3.124924	3.917142 L
H	-7.449688	-2.640925	3.682170 L
H	-6.002662	-3.330201	2.926131 L
C	-6.694195	-4.440020	4.670584 L
H	-5.729359	-4.976458	4.773110 L
H	-7.031202	-4.228121	5.704477 L
C	-7.728632	-5.345590	3.987464 L
H	-8.636639	-4.763579	3.736465 L
H	-8.061683	-6.118828	4.708036 L
C	-7.186812	-6.027219	2.724906 L
H	-6.870777	-5.263424	1.987000 L
H	-6.263359	-6.607834	2.969712 L
C	-8.215467	-6.965498	2.099070 L
H	-9.132390	-6.429396	1.796334 L
H	-7.812964	-7.448356	1.201243 L
H	-8.515111	-7.758584	2.792478 L
Pd	7.750947	-2.409667	-3.062671 L
N	6.478725	-2.336615	-4.641069 L
N	8.104321	-0.472960	-3.601979 L
N	9.089400	-2.675391	-1.549183 L
O	7.152195	-4.310908	-2.808775 L
O	4.124777	-6.474972	0.175925 L
C	5.630209	-3.322424	-5.020876 L
H	5.606568	-4.209133	-4.358532 L
C	4.851941	-3.221714	-6.182196 L
H	4.172861	-4.021443	-6.461139 L
C	4.976198	-2.073711	-6.975469 L
H	4.385970	-1.979139	-7.887586 L
C	5.852886	-1.059537	-6.602029 L
H	5.975646	-0.161293	-7.211098 L
C	6.614697	-1.191997	-5.416618 L
C	7.518545	-0.223153	-4.879211 L
C	7.911065	1.077730	-5.321617 L
C	8.683862	1.636590	-4.298133 L
H	9.142207	2.598659	-4.284226 L
C	8.766383	0.660538	-3.249428 L
C	9.442339	0.862309	-1.949375 L
H	8.973093	0.276800	-1.133600 L
H	9.427276	1.923256	-1.641780 L
H	10.500888	0.564545	-1.999663 L
C	7.587465	1.727889	-6.611826 L
H	7.393255	2.801734	-6.484897 L
H	6.705459	1.284083	-7.099557 L
H	8.431043	1.631659	-7.312561 L
C	10.400072	-2.099325	-1.667806 L
C	10.919941	-1.973451	-2.972204 L
H	10.328320	-2.330349	-3.828254 L
C	12.161346	-1.369431	-3.161232 L
H	12.556775	-1.258488	-4.170358 L
C	12.896466	-0.887179	-2.064101 L
C	12.378372	-1.031247	-0.768428 L
H	12.944585	-0.665717	0.086532 L
C	11.134269	-1.629699	-0.564562 L
H	10.714887	-1.685312	0.441470 L
C	14.211380	-0.189399	-2.273953 L
H	14.743572	-0.616185	-3.148394 L
H	14.885944	-0.364201	-1.411463 L
C	13.999074	1.320044	-2.478783 L
H	13.382639	1.488805	-3.383956 L
H	13.406144	1.742704	-1.637074 L
C	15.339206	2.054561	-2.606387 L
H	15.919100	1.637592	-3.452929 L
H	15.956139	1.878424	-1.704424 L
C	15.138030	3.561629	-2.811201 L
H	14.566057	3.978348	-1.958402 L
H	14.512590	3.739635	-3.707389 L
C	16.467428	4.318113	-2.946793 L
H	16.281139	5.402801	-2.815607 L
H	17.150668	4.030316	-2.123901 L
C	17.151763	4.081589	-4.291380 L
H	17.386020	3.021647	-4.442986 L
H	18.091365	4.640198	-4.362533 L
H	16.516523	4.396298	-5.127168 L
C	8.806221	-3.480565	-0.548113 L
H	9.518235	-3.661579	0.279749 L
C	7.561633	-4.200607	-0.453144 L
C	6.797856	-4.594967	-1.608037 L
C	5.625920	-5.403042	-1.429445 L
H	5.097141	-5.715909	-2.316158 L
C	5.237487	-5.747055	-0.149533 L
C	5.967151	-5.342696	1.005013 L
H	5.618591	-5.628701	1.999649 L
C	7.106178	-4.592120	0.832628 L
H	7.666297	-4.272129	1.718022 L
C	3.261666	-6.961009	-0.902696 L
H	3.853296	-7.643750	-1.534217 L
H	2.919326	-6.089215	-1.488713 L
C	2.114806	-7.647781	-0.169192 L
H	1.230225	-7.701529	-0.849099 L
H	1.788662	-7.009532	0.681714 L
C	2.487705	-9.042604	0.343125 L
H	3.280732	-8.936213	1.120824 L
H	2.945144	-9.635963	-0.471054 L
C	1.283773	-9.804848	0.913570 L
H	0.393870	-9.650198	0.273779 L
H	1.491801	-10.892168	0.875064 L
C	0.960702	-9.400750	2.358755 L
H	0.787308	-8.306799	2.413543 L
H	1.838800	-9.597601	3.006486 L
C	-0.256635	-10.142977	2.905311 L
H	-1.196118	-9.767702	2.461849 L
H	-0.340975	-10.013601	3.990556 L
H	-0.202953	-11.218370	2.707781 L
Pd	-9.047510	2.391577	-1.045306 L
N	-7.670568	2.723516	0.405356 L
N	-9.589530	0.867814	0.183668 L
N	-10.476756	2.257278	-2.499671 L
O	-8.199692	3.891126	-2.089688 L
O	-5.295932	4.269019	-5.851769 L
C	-6.708172	3.680556	0.379227 L
H	-6.669508	4.291168	-0.542692 L
C	-5.833657	3.876971	1.454369 L
H	-5.061113	4.641509	1.407223 L
C	-5.974015	3.070542	2.592465 L
H	-5.298080	3.207780	3.441516 L
C	-6.970533	2.100099	2.637581 L
H	-7.106909	1.468873	3.518642 L
C	-7.828298	1.922723	1.527066 L
C	-8.870803	0.944824	1.410537 L
C	-9.317201	-0.100842	2.273278 L
C	-10.275124	-0.832978	1.560522 L
H	-10.820521	-1.685291	1.895999 L
C	-10.402802	-0.219692	0.272576 L
C	-11.257397	-0.718197	-0.831110 L
H	-10.879863	-0.412207	-1.816824 L
H	-11.301438	-1.825438	-0.816383 L
H	-12.288542	-0.344847	-0.736712 L
C	-8.872942	-0.393661	3.655062 L
H	-8.415070	-1.400030	3.718087 L
H	-8.134811	0.335105	4.024880 L
H	-9.724449	-0.380273	4.350648 L
C	-11.843020	2.081422	-2.088232 L
C	-12.170472	2.541287	-0.795689 L
H	-11.394875	3.012743	-0.170104 L
C	-13.468546	2.382850	-0.316787 L
H	-13.717899	2.732240	0.685128 L
C	-14.454924	1.769907	-1.109437 L
C	-14.120543	1.312997	-2.392586 L
H	-14.876070	0.830741	-3.011358 L
C	-12.823808	1.467770	-2.886949 L
H	-12.578775	1.099559	-3.880825 L

C	-15.850765	1.593739	-0.579033	L
H	-16.125056	2.444499	0.077891	L
H	-16.588560	1.618146	-1.406472	L
C	-15.976040	0.272165	0.196497	L
H	-15.239660	0.254641	1.024341	L
H	-15.704364	-0.574689	-0.463157	L
C	-17.393910	0.078348	0.748046	L
H	-17.663067	0.925896	1.408059	L
H	-18.129159	0.096633	-0.079114	L
C	-17.516650	-1.239743	1.524330	L
H	-17.240601	-2.086628	0.866207	L
H	-16.785775	-1.256096	2.356075	L
C	-18.932731	-1.467387	2.073051	L
H	-19.035816	-2.528103	2.377931	L
H	-19.678225	-1.313954	1.268342	L
C	-19.262646	-0.565531	3.260397	L
H	-19.210229	0.494736	2.987883	L
H	-20.272938	-0.759188	3.637172	L
H	-18.565152	-0.722409	4.091072	L
C	-10.189563	2.544966	-3.751886	L
H	-10.958084	2.529926	-4.548145	L
C	-8.871241	2.929251	-4.180368	L
C	-7.942737	3.616153	-3.315238	L
C	-6.708292	4.097589	-3.865430	L
H	-6.045450	4.635904	-3.203360	L
C	-6.420706	3.867571	-5.196050	L
C	-7.318725	3.160636	-6.057986	L
H	-7.032212	2.998804	-7.090850	L
C	-8.511261	2.722664	-5.538600	L
H	-9.213811	2.193666	-6.186914	L
C	-4.251941	4.978215	-5.097050	L
H	-4.705189	5.855058	-4.608749	L
H	-3.851119	4.274875	-4.344486	L
C	-3.219335	5.335620	-6.158818	L
H	-2.223742	5.435467	-5.676241	L
H	-3.123125	4.486822	-6.870625	L
C	-3.580086	6.615784	-6.921215	L
H	-4.589053	6.504162	-7.367479	L
H	-3.654793	7.463883	-6.213336	L
C	-2.554954	6.956649	-8.010771	L
H	-1.530277	6.910618	-7.593510	L
H	-2.699644	8.006648	-8.331130	L
C	-2.662929	6.029128	-9.229286	L
H	-2.513445	4.977274	-8.913589	L
H	-3.688491	6.073049	-9.644939	L
C	-1.648348	6.393705	-10.311030	L
H	-0.620184	6.321358	-9.938788	L
H	-1.732828	5.723219	-11.173339	L
H	-1.794992	7.416946	-10.673826	L
Pd	5.551851	-3.957360	4.301132	L
N	7.004387	-4.280925	5.683960	L
N	5.210392	-5.943068	4.581834	L
N	4.079385	-3.407162	3.002037	L
O	6.175764	-2.025770	4.251912	L
O	9.245996	0.085043	1.259321	L
C	7.857034	-3.342032	6.163546	L
H	7.776678	-2.343682	5.693877	L
C	8.766754	-3.623722	7.190018	L
H	9.441066	-2.856485	7.552943	L
C	8.779548	-4.914434	7.739636	L
H	9.476991	-5.154459	8.542079	L
C	7.903814	-5.882679	7.264513	L
H	7.888766	-6.892091	7.681793	L
C	7.001827	-5.563084	6.219169	L
C	6.052583	-6.443675	5.621785	L
C	5.767925	-7.831910	5.815699	L
C	4.773737	-8.174560	4.896146	L
H	4.320959	-9.129267	4.755597	L
C	4.462584	-6.990774	4.146696	L
C	3.488082	-6.919751	3.037478	L
H	3.694623	-6.087281	2.341868	L
H	3.519099	-7.837283	2.419924	L
H	2.461215	-6.794635	3.409476	L
C	6.400610	-8.756777	6.783824	L
H	7.091250	-9.442000	6.270224	L
H	6.974762	-8.227836	7.560517	L
H	5.647166	-9.374431	7.292450	L
C	2.731978	-3.845874	3.243462	L
C	2.428738	-4.242904	4.559671	L
H	3.201187	-4.188350	5.342826	L
C	1.152290	-4.721167	4.858565	L
H	0.926550	-5.041763	5.874703	L
C	0.167141	-4.797819	3.861826	L
C	0.470983	-4.370567	2.557269	L
H	-0.298511	-4.401613	1.777471	L
C	1.744707	-3.905596	2.241767	L
H	1.965983	-3.602398	1.218730	L
C	-1.203223	-5.332825	4.170917	L
H	-1.310604	-5.567766	5.247577	L
H	-1.956627	-4.539265	3.967856	L
C	-1.507036	-6.582571	3.329552	L
H	-0.821921	-7.402776	3.619168	L
H	-1.292778	-6.373859	2.258030	L
C	-2.965557	-7.031559	3.475600	L
H	-3.157281	-7.385164	4.504989	L
H	-3.639215	-6.159223	3.313813	L
C	-3.306880	-8.128999	2.458996	L
H	-3.247689	-7.700389	1.429882	L
H	-2.533960	-8.931690	2.489415	L
C	-4.698680	-8.728918	2.685745	L
H	-5.024290	-9.256108	1.767895	L
H	-5.440177	-7.907167	2.838478	L
C	-4.747087	-9.690655	3.870620	L
H	-4.444798	-9.195881	4.800949	L
H	-5.756704	-10.087547	4.019564	L
H	-4.073921	-10.542793	3.723821	L
C	4.343163	-2.521113	2.070527	L
H	3.579825	-2.158233	1.357073	L
C	5.654894	-1.922358	1.921975	L
C	6.506367	-1.684096	3.061807	L
C	7.735834	-0.968286	2.870638	L
H	8.338273	-0.772098	3.743791	L
C	8.084630	-0.570609	1.595595	L
C	7.277952	-0.833906	0.458704	L
H	7.615914	-0.523913	-0.533381	L
C	6.079799	-1.496704	0.645732	L
H	5.450698	-1.705993	-0.225152	L
C	10.108167	0.589081	2.328062	L
H	9.488577	1.048099	3.116591	L
H	10.662262	-0.273081	2.734241	L
C	11.009272	1.591713	1.610435	L
H	11.941597	1.738131	2.186847	L
H	11.312687	1.163972	0.628959	L
C	10.295894	2.930123	1.386872	L
H	9.349331	2.745918	0.835443	L
H	9.994484	3.360655	2.362027	L
C	11.151866	3.951416	0.630238	L
H	12.118116	4.102363	1.145826	L
H	10.631050	4.940647	0.673506	L
C	11.396448	3.570388	-0.835357	L
H	12.165146	2.768549	-0.902353	L
H	10.469607	3.129452	-1.272657	L
C	11.831944	4.772811	-1.668578	L
H	12.737536	5.239959	-1.265945	L
H	12.041553	4.480287	-2.704204	L
H	11.052727	5.544025	-1.699871	L

Triplet: the high level layer was computed as an unrestricted **triplet** state. The low level layer as a restricted singlet state.

Pd	1.201396	-1.745439	0.576035	H
N	-0.475603	-2.300842	1.590253	H
N	1.386912	-3.751504	0.486118	H
N	2.935762	-1.216941	-0.239771	H
O	0.740725	0.202167	0.688381	H
O	0.023669	4.559731	-1.217342	H
C	-1.429183	-1.436936	1.995764	H
H	-1.250909	-0.397859	1.725811	H
C	-2.538890	-1.867164	2.727031	H
H	-3.304562	-1.158541	3.016921	H
C	-2.612834	-3.223707	3.091788	H
H	-3.452749	-3.582731	3.677020	H
C	-1.607294	-4.108454	2.704041	H

H	-1.646656	-5.149437	2.987570	H
C	-0.525568	-3.638142	1.927679	H
C	0.529712	-4.428390	1.363666	H
C	0.853782	-5.801960	1.371277	H
C	1.898550	-5.948754	0.428066	H
H	2.388270	-6.873421	0.158211	H
C	2.199514	-4.679587	-0.111285	H
C	3.187212	-4.377655	-1.193215	H
H	2.867517	-3.521802	-1.798922	H
H	3.286427	-5.240663	-1.865198	H
H	4.183553	-4.154599	-0.797409	H
C	0.265360	-6.902245	2.206009	H
H	-0.762940	-7.162389	1.902002	H
H	0.237593	-6.634668	3.269107	H
H	0.868013	-7.809796	2.112862	H
C	4.152825	-1.593157	0.268900	H
C	4.226895	-2.488747	1.381415	H
H	3.307988	-2.873956	1.802170	H
C	5.455344	-2.875396	1.904079	H
H	5.478222	-3.557468	2.747811	H
C	6.668673	-2.398547	1.363889	H
C	6.595471	-1.504078	0.269366	H
H	7.519851	-1.110826	-0.151272	H
C	5.382700	-1.100690	-0.269173	H
H	5.364289	-0.386743	-1.084184	H
C	8.011544	-2.831585	1.896222	H
H	7.933156	-3.106483	2.953947	H
H	8.711428	-1.987124	1.831966	H
C	8.610239	-4.019534	1.106953	H
H	7.998083	-4.914512	1.277558	H
H	8.547398	-3.800270	0.031643	H
C	10.073780	-4.296044	1.483314	H
H	10.159983	-4.386875	2.573793	H
H	10.682658	-3.420616	1.190747	H
C	10.633178	-5.554546	0.802316	H
H	10.389721	-5.507427	-0.273776	H
H	10.111388	-6.439073	1.195090	H
C	12.150794	-5.744981	0.968697	H
H	12.467971	-6.604700	0.364313	H
H	12.670930	-4.868911	0.557454	H
C	12.593209	-5.966772	2.423272	H
H	12.377523	-5.094196	3.048457	H
H	13.669926	-6.158528	2.482595	H
H	12.074995	-6.828325	2.860953	H
C	2.848067	-0.318277	-1.339010	H
H	3.432694	-0.545541	-2.227422	H
C	2.055404	0.844279	-1.283878	H
C	1.089849	1.111563	-0.233619	H
C	0.431712	2.361075	-0.183770	H
H	-0.221627	2.525849	0.673725	H
C	0.649561	3.325411	-1.165539	H
C	1.526375	3.047945	-2.247021	H
H	1.669767	3.803960	-3.014600	H
C	2.209021	1.850820	-2.297492	H
H	2.898567	1.667526	-3.122610	H
C	-0.724848	5.027760	-0.055659	H
H	-0.041538	5.094331	0.808164	H
H	-1.520517	4.311033	0.185327	H
C	-1.315889	6.386545	-0.404825	H
H	-1.930593	6.706863	0.448757	H
H	-1.986608	6.264059	-1.260821	H
C	-0.251746	7.454131	-0.698931	H
H	0.366035	7.114324	-1.546048	H
H	0.419930	7.523990	0.169342	H
C	-0.829908	8.846215	-1.007060	H
H	-1.472234	9.170988	-0.175018	H
H	-0.002978	9.568090	-1.057002	H
C	-1.623993	8.922804	-2.322599	H
H	-2.496152	8.259388	-2.272071	H
H	-0.994273	8.546632	-3.140861	H
C	-2.091242	10.348064	-2.649176	H
H	-2.732696	10.744066	-1.853154	H
H	-2.663955	10.377461	-3.590651	H
H	-1.236079	11.025781	-2.755460	H
Pd	0.102605	1.373890	3.396284	L
N	2.059283	1.495627	2.915402	L
N	0.198323	3.403463	3.221367	L
N	-1.875634	1.199024	3.931099	L
O	0.282562	-0.601493	3.852474	L
O	1.783055	-1.946173	8.188116	L
C	2.903359	0.441805	2.782362	L
H	2.434344	-0.563708	2.868448	L
C	4.269847	0.631302	2.526814	L
H	4.941666	-0.225009	2.466648	L
C	4.752903	1.928317	2.327937	L
H	5.814752	2.091020	2.112752	L
C	3.876656	3.011170	2.404530	L
H	4.220793	4.029529	2.213655	L
C	2.521759	2.788774	2.725330	L
C	1.523466	3.809731	2.913290	L
C	1.592227	5.231309	2.929595	L
C	0.309686	5.693443	3.266680	L
H	0.001285	6.706831	3.379251	L
C	-0.516077	4.542449	3.455777	L
C	-1.937396	4.565949	3.877112	L
H	-2.146325	3.794051	4.632160	L
H	-2.201243	5.535683	4.319802	L
H	-2.615607	4.393068	3.028572	L
C	2.764602	6.093132	2.652573	L
H	3.083034	6.625225	3.560114	L
H	3.628163	5.521902	2.276110	L
H	2.523484	6.854347	1.896802	L
C	-2.913284	1.704906	3.077999	L
C	-2.541454	2.007265	1.753483	L
H	-1.497413	1.834629	1.420610	L
C	-3.481924	2.534347	0.868657	L
H	-3.191726	2.751274	-0.158821	L
C	-4.797787	2.779097	1.291013	L
C	-5.162782	2.471177	2.611395	L
H	-6.192281	2.641371	2.945233	L
C	-4.236491	1.932286	3.501497	L
H	-4.552008	1.692095	4.516829	L
C	-5.814634	3.380338	0.360423	L
H	-5.664657	3.016700	-0.675317	L
H	-6.832829	3.032000	0.639060	L
C	-5.744243	4.916005	0.403304	L
H	-4.876169	5.262756	-0.188609	L
H	-5.552723	5.251448	1.442668	L
C	-7.036328	5.564497	-0.110645	L
H	-7.306686	5.150396	-1.100289	L
H	-7.876773	5.304191	0.562641	L
C	-6.892705	7.089889	-0.207244	L
H	-6.355038	7.473033	0.683164	L
H	-6.256370	7.348831	-1.074988	L
C	-8.241178	7.816116	-0.314851	L
H	-8.059032	8.909643	-0.299952	L
H	-8.853148	7.602321	0.583925	L
C	-9.029378	7.449438	-1.569353	L
H	-9.448081	6.430136	-1.492799	L
H	-9.868555	8.135273	-1.726461	L
H	-8.402719	7.481493	-2.466765	L
C	-2.140671	0.493779	5.009219	L
H	-3.166135	0.325364	5.386904	L
C	-1.077321	-0.122397	5.768171	L
C	0.124268	-0.615441	5.127272	L
C	1.119701	-1.255718	5.939617	L
H	1.999165	-1.627102	5.432519	L
C	0.914265	-1.370518	7.298479	L
C	-0.266007	-0.890749	7.941327	L
H	-0.367918	-1.009885	9.012466	L
C	-1.228793	-0.286988	7.163331	L
H	-2.139135	0.086329	7.637344	L
C	3.051628	-2.473638	7.677632	L
H	2.830759	-3.236168	6.912432	L
H	3.613171	-1.634682	7.231502	L
C	3.730116	-3.034953	8.923572	L
H	4.828818	-2.998801	8.795443	L
H	3.500686	-2.369663	9.785490	L
C	3.274588	-4.464104	9.242301	L
H	2.170170	-4.479956	9.346376	L
H	3.505173	-5.130666	8.388731	L
C	3.932670	-5.020122	10.511660	L
H	5.028481	-4.871117	10.468304	L
H	3.782922	-6.117063	10.547645	L
C	3.370330	-4.385189	11.791324	L
H	3.512189	-3.286727	11.759563	L

H	2.274773	-4.542529	11.837492 L
C	4.029558	-4.956507	13.044661 L
H	5.111119	-4.781039	13.047724 L
H	3.620099	-4.496274	13.950830 L
H	3.872451	-6.037841	13.126374 L
Pd	2.416931	3.027613	-5.523120 L
N	4.402181	3.290955	-5.866648 L
N	2.522759	4.998598	-4.986106 L
N	0.420468	2.596978	-5.370400 L
O	2.707322	1.124770	-6.121942 L
O	3.108040	-3.200445	-4.158055 L
C	5.261729	2.330619	-6.292339 L
H	4.833886	1.313818	-6.378871 L
C	6.593894	2.618071	-6.608428 L
H	7.264740	1.831057	-6.936474 L
C	7.038774	3.944775	-6.500264 L
H	8.072058	4.191399	-6.746296 L
C	6.164341	4.939616	-6.081485 L
H	6.484740	5.981153	-5.998530 L
C	4.826033	4.608322	-5.754053 L
C	3.825268	5.507877	-5.279056 L
C	3.861806	6.898721	-4.948790 L
C	2.603471	7.227062	-4.437734 L
H	2.274679	8.178149	-4.085070 L
C	1.810909	6.031924	-4.460462 L
C	0.433798	5.925058	-3.930633 L
H	0.145641	4.888379	-3.692996 L
H	0.340093	6.481888	-2.973751 L
H	-0.306442	6.329902	-4.633605 L
C	5.001854	7.832611	-5.090263 L
H	5.441654	8.061119	-4.107931 L
H	5.804896	7.428255	-5.726284 L
H	4.680468	8.786620	-5.531817 L
C	-0.523185	3.584829	-5.819806 L
C	-0.070976	4.472635	-6.816538 L
H	0.947677	4.367286	-7.222356 L
C	-0.910029	5.492977	-7.261706 L
H	-0.550593	6.187184	-8.021050 L
C	-2.202001	5.640972	-6.729473 L
C	-2.651724	4.737574	-5.753400 L
H	-3.655669	4.838209	-5.342725 L
C	-1.821689	3.714208	-5.295319 L
H	-2.177074	3.036349	-4.520798 L
C	-3.092084	6.762912	-7.188532 L
H	-2.656431	7.275225	-8.070372 L
H	-4.062415	6.354377	-7.539176 L
C	-3.326581	7.781532	-6.060725 L
H	-2.352734	8.106687	-5.642544 L
H	-3.863449	7.295513	-5.223299 L
C	-4.113907	9.000928	-6.556635 L
H	-3.506413	9.567600	-7.289243 L
H	-5.018843	8.675965	-7.104873 L
C	-4.516459	9.917239	-5.394104 L
H	-5.214654	9.380770	-4.722865 L
H	-3.623459	10.152937	-4.766413 L
C	-5.170840	11.220802	-5.871700 L
H	-5.700780	11.693453	-5.020620 L
H	-5.953063	10.996843	-6.623320 L
C	-4.164509	12.210952	-6.453981 L
H	-3.632997	11.786363	-7.313600 L
H	-4.659381	13.127732	-6.792485 L
H	-3.407894	12.498126	-5.714886 L
C	0.016422	1.392566	-5.033193 L
H	-1.056598	1.126021	-4.972031 L
C	0.926889	0.306444	-4.749615 L
C	2.224042	0.209888	-5.369772 L
C	2.995550	-0.986044	-5.186916 L
H	3.943618	-1.057807	-5.701827 L
C	2.494090	-1.997043	-4.393592 L
C	1.252246	-1.883666	-3.706712 L
H	0.921655	-2.685742	-3.049312 L
C	0.491590	-0.753565	-3.917472 L
H	-0.478462	-0.666894	-3.419645 L
C	4.313499	-3.560430	-4.897931 L
H	4.111420	-4.625959	-5.126465 L
H	4.390072	-2.987857	-5.835941 L
C	5.519183	-3.382535	-3.979659 L
H	5.763511	-2.309630	-3.839627 L

H	5.259779	-3.749600	-2.963387 L
C	6.733222	-4.138920	-4.535117 L
H	6.410129	-5.083212	-5.018556 L
H	7.205081	-3.539178	-5.337849 L
C	7.781693	-4.462579	-3.462803 L
H	8.053006	-3.545571	-2.901565 L
H	8.714292	-4.785270	-3.966590 L
C	7.314949	-5.549514	-2.482864 L
H	6.736134	-5.085777	-1.659061 L
H	6.605375	-6.253175	-2.983191 L
C	8.482788	-6.346634	-1.909130 L
H	9.210406	-5.696086	-1.394897 L
H	8.134289	-7.083376	-1.176337 L
H	9.024575	-6.890074	-2.690620 L
Pd	-7.898490	-1.906509	3.098242 L
N	-6.550731	-1.759962	4.607895 L
N	-8.085004	0.092332	3.431797 L
N	-9.346708	-2.235942	1.695270 L
O	-7.462354	-3.869151	3.020177 L
O	-4.752599	-6.544006	0.151435 L
C	-5.785341	-2.772648	5.081915 L
H	-5.870078	-3.728864	4.530519 L
C	-4.960205	-2.613323	6.203511 L
H	-4.345136	-3.435638	6.556891 L
C	-4.950362	-1.375232	6.858641 L
H	-4.321373	-1.233670	7.738696 L
C	-5.743136	-0.331467	6.389579 L
H	-5.769551	0.635207	6.897652 L
C	-6.554814	-0.526095	5.247179 L
C	-7.396524	0.450808	4.629198 L
C	-7.662697	1.824111	4.919254 L
C	-8.472562	2.307382	3.885043 L
H	-8.866209	3.291914	3.777469 L
C	-8.697114	1.217829	2.978941 L
C	-9.447585	1.297199	1.706781 L
H	-9.174357	0.486962	1.004412 L
H	-9.245562	2.242383	1.172811 L
H	-10.532469	1.235746	1.876962 L
C	-7.180054	2.616021	6.073501 L
H	-6.530901	3.440016	5.743989 L
H	-6.611635	2.010655	6.796728 L
H	-8.023249	3.066901	6.617673 L
C	-10.620909	-1.592782	1.860343 L
C	-10.999644	-1.289024	3.184087 L
H	-10.329038	-1.553277	4.017030 L
C	-12.209085	-0.640189	3.422413 L
H	-12.492933	-0.397352	4.446132 L
C	-13.056349	-0.289936	2.356879 L
C	-12.671454	-0.595977	1.042619 L
H	-13.321813	-0.328180	0.211610 L
C	-11.459845	-1.240786	0.788165 L
H	-11.158121	-1.445816	-0.237990 L
C	-14.360420	0.409891	2.624673 L
H	-14.587132	0.410944	3.710578 L
H	-15.193133	-0.153636	2.155333 L
C	-14.340281	1.853951	2.098198 L
H	-13.418973	2.364516	2.444095 L
H	-14.284076	1.847786	0.992758 L
C	-15.574103	2.639080	2.561484 L
H	-15.543156	2.770276	3.661053 L
H	-16.496011	2.062779	2.354313 L
C	-15.656601	4.008580	1.875260 L
H	-15.839851	3.869338	0.792086 L
H	-14.672926	4.529705	1.944509 L
C	-16.758635	4.898036	2.466859 L
H	-16.974444	5.725811	1.762184 L
H	-17.702100	4.323519	2.550468 L
C	-16.385499	5.477533	3.829579 L
H	-16.180466	4.686778	4.560785 L
H	-17.193876	6.097263	4.232690 L
H	-15.488164	6.103698	3.769581 L
C	-9.171905	-3.150722	0.765908 L
H	-9.942584	-3.371080	0.003409 L
C	-7.978798	-3.955462	0.682583 L
C	-7.194265	-4.298248	1.840266 L
C	-6.103737	-5.218329	1.694815 L
H	-5.570301	-5.500528	2.589347 L
C	-5.792445	-5.699529	0.437877 L

C	-6.524943	-5.329698	-0.726210 L
H	-6.224645	-5.707581	-1.706487 L
C	-7.599295	-4.482878	-0.579242 L
H	-8.168724	-4.192581	-1.467447 L
C	-3.946586	-7.058214	1.261526 L
H	-4.594976	-7.704679	1.875682 L
H	-3.583009	-6.200858	1.855528 L
C	-2.807373	-7.803194	0.576939 L
H	-1.928343	-7.833377	1.266114 L
H	-2.464354	-7.214504	-0.303651 L
C	-3.187701	-9.218933	0.132703 L
H	-3.878563	-9.148615	-0.741953 L
H	-3.764339	-9.728532	0.928009 L
C	-1.964548	-10.071051	-0.229112 L
H	-1.249972	-10.084832	0.616398 L
H	-2.285003	-11.122863	-0.365346 L
C	-1.252376	-9.586464	-1.499805 L
H	-0.925441	-8.534723	-1.373280 L
H	-1.962398	-9.579768	-2.350562 L
C	-0.046970	-10.460859	-1.839633 L
H	0.695347	-10.454651	-1.033655 L
H	0.453411	-10.105032	-2.747685 L
H	-0.338799	-11.502563	-2.012069 L
Pd	8.996820	2.290367	1.067328 L
N	7.535720	2.823054	-0.234443 L
N	9.330230	0.835101	-0.306586 L
N	10.514513	1.943149	2.399780 L
O	8.350085	3.756245	2.293966 L
O	5.606898	3.819101	6.195028 L
C	6.679393	3.864905	-0.077207 L
H	6.766297	4.410453	0.880690 L
C	5.756141	4.216035	-1.068814 L
H	5.067196	5.043099	-0.916507 L
C	5.731800	3.477486	-2.260519 L
H	5.016628	3.739256	-3.046796 L
C	6.615079	2.415948	-2.437278 L
H	6.627100	1.837935	-3.363870 L
C	7.522318	2.080575	-1.405807 L
C	8.461815	0.996927	-1.423327 L
C	8.670658	-0.082916	-2.333188 L
C	9.654107	-0.904571	-1.766512 L
H	10.058721	-1.802895	-2.172630 L
C	10.022875	-0.319248	-0.512002 L
C	10.976574	-0.896646	0.464050 L
H	10.750537	-0.582521	1.493172 L
H	10.937783	-2.003317	0.448900 L
H	12.010353	-0.594155	0.240011 L
C	7.959317	-0.333532	-3.608450 L
H	7.154966	-1.076939	-3.468915 L
H	7.499514	0.581981	-4.013612 L
H	8.638640	-0.731443	-4.374215 L
C	11.841269	1.748548	1.881770 L
C	12.097389	2.289312	0.604504 L
H	11.300369	2.833376	0.070956 L
C	13.351496	2.119261	0.023251 L
H	13.545153	2.532132	-0.966649 L
C	14.363896	1.413980	0.697230 L
C	14.100332	0.876344	1.965559 L
H	14.875835	0.321813	2.492177 L
C	12.848731	1.041948	2.561866 L
H	12.658179	0.610641	3.542245 L
C	15.711267	1.226213	0.056752 L
H	15.971908	2.109124	-0.562089 L
H	16.505326	1.169753	0.828686 L
C	15.730496	-0.043961	-0.809245 L
H	14.938684	0.019710	-1.581756 L
H	15.471515	-0.923134	-0.187770 L
C	17.098228	-0.249872	-1.471849 L
H	17.354714	0.630094	-2.093304 L
H	17.888737	-0.312974	-0.699693 L
C	17.114804	-1.516428	-2.338221 L
H	16.850642	-2.395229	-1.718105 L
H	16.328595	-1.451158	-3.115309 L
C	18.479771	-1.756872	-2.999435 L
H	18.519049	-2.797684	-3.378719 L
H	19.284043	-1.684495	-2.241315 L
C	18.763729	-0.789513	-4.146450 L
H	18.772622	0.251185	-3.802883 L
H	19.737260	-0.993218	-4.605609 L
H	18.005222	-0.865280	-4.933884 L
C	10.316876	2.120700	3.688845 L
H	11.125980	1.993616	4.432439 L
C	9.043437	2.521373	4.227218 L
C	8.121140	3.350717	3.488901 L
C	6.936832	3.822984	4.145779 L
H	6.278864	4.467045	3.580637 L
C	6.687571	3.448654	5.451236 L
C	7.580590	2.606497	6.186255 L
H	7.326852	2.338607	7.205310 L
C	8.728187	2.175853	5.566959 L
H	9.426806	1.542122	6.118226 L
C	4.569653	4.650773	5.567755 L
H	5.044786	5.554316	5.154582 L
H	4.104323	4.051750	4.763090 L
C	3.600655	4.937440	6.708292 L
H	2.593085	5.136752	6.287361 L
H	3.490397	4.020407	7.327867 L
C	4.058958	6.106426	7.587964 L
H	5.077009	5.895949	7.974230 L
H	4.152438	7.022867	6.973895 L
C	3.096980	6.374949	8.752714 L
H	2.056283	6.430952	8.378787 L
H	3.313998	7.372554	9.181449 L
C	3.197322	5.310563	9.854429 L
H	2.975040	4.310742	9.430713 L
H	4.238788	5.252105	10.226439 L
C	2.247483	5.603432	11.013904 L
H	1.203358	5.628152	10.681989 L
H	2.325750	4.835520	11.791461 L
H	2.466628	6.569456	11.481663 L
Pd	-5.675731	-4.222593	-4.053848 L
N	-7.140345	-4.374252	-5.455994 L
N	-5.614246	-6.233681	-4.280687 L
N	-4.168514	-3.875898	-2.726305 L
O	-5.999070	-2.220085	-4.047035 L
O	-8.861973	0.310188	-1.184159 L
C	-7.842937	-3.338810	-5.977216 L
H	-7.617125	-2.346988	-5.543011 L
C	-8.781474	-3.521989	-7.000429 L
H	-9.332734	-2.677881	-7.398468 L
C	-8.985078	-4.817021	-7.499905 L
H	-9.708644	-4.981907	-8.298140 L
C	-8.264778	-5.885807	-6.980805 L
H	-8.399879	-6.902196	-7.357715 L
C	-7.327101	-5.663067	-5.942043 L
C	-6.517801	-6.649590	-5.305394 L
C	-6.426524	-8.070211	-5.446542 L
C	-5.486476	-8.512077	-4.511911 L
H	-5.168462	-9.514118	-4.336086 L
C	-5.013656	-7.355111	-3.806159 L
C	-4.030123	-7.359481	-2.702723 L
H	-4.205385	-6.540293	-1.981838 L
H	-4.087673	-8.295836	-2.116266 L
H	-3.001504	-7.258454	-3.078181 L
C	-7.181498	-8.933532	-6.383358 L
H	-7.932883	-9.528072	-5.842979 L
H	-7.710010	-8.356032	-7.157868 L
H	-6.513633	-9.640324	-6.895643 L
C	-2.870681	-4.442276	-2.980277 L
C	-2.594087	-4.773215	-4.321242 L
H	-3.372102	-4.645589	-5.090920 L
C	-1.330622	-5.251674	-4.665890 L
H	-1.118372	-5.496701	-5.705589 L
C	-0.330904	-5.404089	-3.690033 L
C	-0.635284	-5.124030	-2.347322 L
H	0.128547	-5.260781	-1.572744 L
C	-1.895563	-4.649198	-1.987276 L
H	-2.110780	-4.438561	-0.939942 L
C	1.063913	-5.787806	-4.095333 L
H	1.122876	-5.962298	-5.187951 L
H	1.728302	-4.906731	-3.907339 L
C	1.607706	-7.017423	-3.353349 L
H	1.083355	-7.927461	-3.699259 L
H	1.390072	-6.939050	-2.267144 L
C	3.122274	-7.146925	-3.568719 L
H	3.350826	-7.182659	-4.650049 L

H	3.623173	-6.233536	-3.171108	L
C	3.702994	-8.382509	-2.871336	L
H	3.523850	-8.309100	-1.780095	L
H	3.166146	-9.292480	-3.201083	L
C	5.208339	-8.538562	-3.131308	L
H	5.645320	-9.200615	-2.358381	L
H	5.722254	-7.554230	-3.005729	L
C	5.516502	-9.101867	-4.516694	L
H	5.101297	-8.469357	-5.309343	L
H	6.596375	-9.176923	-4.683520	L
H	5.091585	-10.103773	-4.646228	L
C	-4.331361	-2.956247	-1.803408	L
H	-3.541415	-2.691021	-1.076818	L
C	-5.553173	-2.182917	-1.696892	L
C	-6.324541	-1.838406	-2.865900	L
C	-7.450758	-0.962635	-2.719415	L
H	-7.987537	-0.696185	-3.616306	L
C	-7.787737	-0.507409	-1.459237	L
C	-7.060911	-0.863687	-0.294775	L
H	-7.375494	-0.506183	0.687685	L
C	-5.959646	-1.687652	-0.439332	L
H	-5.393724	-1.971160	0.453639	L
C	-9.627120	0.838061	-2.313648	L
H	-8.928473	1.301777	-3.031138	L
H	-10.151393	-0.009336	-2.787374	L
C	-10.590592	1.841660	-1.686739	L
H	-11.408885	2.051702	-2.403021	L
H	-11.070371	1.382154	-0.795742	L
C	-9.889434	3.143521	-1.277266	L
H	-9.277686	2.951571	-0.364337	L
H	-9.166214	3.446938	-2.059189	L
C	-10.870409	4.293376	-1.026594	L
H	-11.543342	4.416107	-1.896662	L
H	-10.295591	5.249533	-0.961268	L
C	-11.700639	4.103809	0.249165	L
H	-12.303475	3.177912	0.169474	L
H	-11.030162	3.949732	1.118401	L
C	-12.616659	5.296947	0.509762	L
H	-13.227521	5.540435	-0.366352	L
H	-13.311300	5.088969	1.342654	L
H	-12.046536	6.194718	0.772188	L

ONIOM computations: reliability of PM6 on 1

Table S9. Some structural parameters obtained after geometry optimization performed with PM6 on **1**. A comparison with the MPW1PW91/SDD09/D95(d) (*in vacuo*) structure and the crystal structure is present. Figure 1a defines the atomic labels. A very good match between DFT and PM6 results is evident.

**Ground state – single molecule *in vacuo***			
**parameter**	**PM6**	**MPW1PW91**	**Experimental (this paper)**
Pd(1)-N(1)	2.025	2.029	2.022(10), 2.029(11)
Pd(1)-N(2)	2.015	2.015	2.042(10), 2.026(10)
Pd(1)-N(3)	2.046	2.046	2.054(10), 2.034(11)
Pd(1)-O(1)	2.029	2.000	2.000(8), 1.988(8)
N(1)-Pd(1)-N(3)	174.1	168.2	172.8(4), 172.2(4)
N(1)-Pd(1)-N(2)	82.7	80.2	80.0(4), 81.1(4)
N(1)-Pd(1)-O(1)	90.0	80.9	86.3(4), 86.2(4)
N(3)-Pd(1)-N(2)	101.1	101.8	102.4(4), 101.5(4)
N(3)-Pd(1)-O(1)	87.5	90.5	91.7(4), 91.6(4)
N(2)-Pd(1)-O(1)	171.1	166.3	165.7(4), 166.6(4)

Table S10. The same structural parameters of Table S9 obtained after geometry optimization of couple a-b of **1** in the crystal (see Figure 8a in the paper). The experimental parameters are reported for the molecules in the couple (an inversion centre exchange the two molecules both in the crystal and in our computations).

With the aim to avoid the drifting and tilting of one molecule respect to the other (impossible in solid phase), some structural parameters were frozen during optimization: the Pd-Pd experimental distance was frozen, like all the angles of D1-Pd1-D2 type, where D1 and D2 are donor atoms on different molecules and Pd1 is the central metal connected to D1.
PM6 and DFT computations are in very good match also in this test and both are accurate in comparison to the experimental values, being the computed values inside the experimental error. This makes confident that PM6 is sufficiently able to describe the intermolecular interactions in the solid phase and their effect on the structure of the single molecule as the DFT would be.

Ground state – couple a-b in the conductive stack of 1			
**parameter**	**PM6**	**MPW1PW91**	**Experimental (this paper)**
Pd(1)-N(1)	2.030	2.027	2.029(11)
Pd(1)-N(2)	2.025	2.021	2.026(10)
Pd(1)-N(3)	2.030	2.027	2.034(11)
Pd(1)-O(1)	1.987	1.983	1.988(8)
N(1)-Pd(1)-N(3)	172.2	172.2	172.2(4)
N(1)-Pd(1)-N(2)	81.1	81.1	81.1(4)
N(1)-Pd(1)-O(1)	86.2	86.2	86.2(4)
N(3)-Pd(1)-N(2)	101.6	101.6	101.5(4)
N(3)-Pd(1)-O(1)	91.6	91.6	91.6(4)
N(2)-Pd(1)-O(1)	166.6	166.5	166.6(4)

Table S11. Computed Cartesian coordinates (MPW1PW91/SD09/D95(d), *in vacuo*) level of approximation of the T_1 excited state of the studied compounds. In all the cases (as discussed in the paper), all the dihedral angles were frozen during the structure optimization. In **1** and **3**, structure optimization was performed starting from the experimental structure, in **2** the computed (*in vacuo*) structure was used as starting point. The Gaussian09 revision D01 was used within the TD-DFT approach. The obtained structure was used for the characterization of the T_1 excited states (Figure 11 and associated discussions in the "Photoconductivity Studies" paragraph).

Complex 1 –TD-DFT optimized T_1

Pd	-0.033721	0.449717	-0.136425
O	-1.991682	0.581077	0.201541
N	0.013508	2.464433	0.151036
N	1.923163	0.781998	-0.555388
N	-0.114424	-1.584625	-0.151180
O	-6.558135	-0.606884	0.008583
C	-1.070361	3.207413	0.405079
H	-2.047405	2.742590	0.349674
C	-0.987393	4.574677	0.741435
H	-1.872678	5.175787	0.913910
C	0.302235	5.110978	0.842573
H	0.421619	6.149200	1.145355
C	1.441498	4.360080	0.564298
H	2.426294	4.807508	0.613701
C	1.281396	3.002176	0.218412
C	2.314736	2.114410	-0.176136
C	3.710911	2.311199	-0.490851
C	4.115384	1.136443	-1.098498
H	5.111478	0.929406	-1.473800
C	2.993628	0.230403	-1.151452
C	4.537710	3.546489	-0.339364
H	5.488752	3.433522	-0.866493
H	4.029404	4.428557	-0.746977
H	4.760345	3.769959	0.713177
C	3.014102	-1.123532	-1.759517
H	2.009423	-1.425703	-2.062673
H	3.659985	-1.125346	-2.644415
H	3.391801	-1.878888	-1.061594
C	1.015190	-2.386316	0.215211
C	1.839529	-1.908861	1.219290
H	1.635532	-0.955757	1.695499
C	2.940478	-2.674336	1.608193
H	3.579012	-2.308715	2.410798
C	3.249024	-3.886560	1.003579
C	2.386698	-4.360854	-0.021847
H	2.584574	-5.320849	-0.493677
C	1.286346	-3.611300	-0.428078
H	0.645470	-3.964554	-1.231191
C	4.460316	-4.699371	1.382399
H	4.334559	-5.744802	1.084237
H	4.600275	-4.715182	2.469173
H	5.376601	-4.313367	0.922138
C	-1.241743	-2.215754	-0.333216
H	-1.165744	-3.302202	-0.414075
C	-2.577449	-1.680083	-0.441339
C	-2.865194	-0.344226	-0.061755
C	-4.233206	0.030040	0.052183
H	-4.490256	1.050794	0.308268
C	-5.246518	-0.895962	-0.159985
C	-4.962271	-2.210218	-0.594313
H	-5.762161	-2.900076	-0.841890
C	-3.644096	-2.586303	-0.687735
H	-3.406421	-3.612930	-0.960385
C	-6.911470	0.638667	0.573431
H	-6.354936	0.823863	1.501576
H	-6.675115	1.466271	-0.109451
H	-7.983306	0.601961	0.770427

Complex 2 –TD-DFT optimized T_1

Pd	-0.703629	-0.649374	-0.159002
N	0.260167	-2.467837	-0.284089
N	1.190652	-0.091070	0.385056
N	-1.579150	1.190517	-0.499237
O	-2.486414	-1.582518	-0.433994
O	-7.112546	-2.199219	0.511118
C	-0.347590	-3.612384	-0.585407
H	-1.432785	-3.578421	-0.558275
C	0.363354	-4.768688	-0.932629
H	-0.170413	-5.685709	-1.161824
C	1.759444	-4.686791	-0.998270
H	2.348095	-5.551343	-1.295578

C	2.398258	-3.488171	-0.690118
H	3.476984	-3.397977	-0.748978
C	1.635038	-2.371060	-0.304140
C	2.150301	-1.061027	0.113104
C	3.429857	-0.553889	0.397171
C	3.214398	0.765005	0.864055
H	3.971994	1.421156	1.276538
C	1.828821	0.999809	0.882008
C	-0.824641	2.266272	-1.044090
C	0.138341	1.994988	-2.024317
H	0.323756	0.964771	-2.316185
C	0.838107	3.036414	-2.626699
H	1.575893	2.802568	-3.392113
C	0.608760	4.373459	-2.271600
C	-0.345556	4.628353	-1.279692
H	-0.528597	5.652184	-0.959510
C	-1.052650	3.595007	-0.666836
H	-1.746093	3.823139	0.138131
C	1.386000	5.489265	-2.919515
H	1.379100	5.398097	-4.011336
H	0.971664	6.468091	-2.660812
C	-2.892727	1.369996	-0.399529
H	-3.272200	2.367200	-0.643348
C	-3.900034	0.390621	-0.080441
C	-3.654754	-1.050331	-0.155098
C	-4.742951	-1.918926	0.032064
H	-4.538009	-2.981458	-0.036556
C	-6.026918	-1.424003	0.303457
C	-6.270212	-0.014176	0.371085
H	-7.276543	0.338430	0.572019
C	-5.215865	0.847538	0.161584
H	-5.393548	1.921223	0.189825
C	-6.948842	-3.602501	0.448880
H	-6.604975	-3.921885	-0.543019
H	-6.243089	-3.957148	1.210607
H	-7.934004	-4.030057	0.643450
H	2.435265	5.477532	-2.600055
C	1.172122	2.132891	1.537999
C	1.771844	3.403872	1.529268
C	-0.005432	1.955816	2.284399
C	1.209002	4.464791	2.235109
H	2.677070	3.560390	0.947758
C	-0.572673	3.019764	2.985382
H	-0.458362	0.969253	2.336272
C	0.030406	4.280876	2.965718
H	1.688022	5.441133	2.212315
H	-1.478601	2.858043	3.565570
H	-0.405925	5.107475	3.521742
C	4.749449	-1.208999	0.338793
C	5.022449	-2.380695	1.064164
C	5.792589	-0.626879	-0.399841
C	6.291128	-2.961386	1.037231
H	4.236944	-2.823872	1.672023
C	7.062820	-1.204812	-0.426143
H	5.594725	0.281623	-0.963976
C	7.317784	-2.377906	0.289102
H	6.482154	-3.864491	1.612519
H	7.854750	-0.739946	-1.009038
H	8.307092	-2.828829	0.269579

Complex 3 –TD-DFT optimized T_1

Pd	-0.254364	0.244481	-0.058652
N	1.487181	0.796693	0.893687
C	1.645284	1.927597	1.601627
H	0.862695	2.675334	1.563016
C	2.767012	2.181375	2.380886
H	2.842695	3.119820	2.919930
C	3.754496	1.208296	2.435131
H	4.657099	1.366795	3.020280
C	3.587632	0.040551	1.745131
H	4.352268	-0.724430	1.791071
C	2.437713	-0.173645	0.980879
C	2.075865	-1.418434	0.290476
C	2.779283	-2.584900	-0.022486
C	4.211411	-2.908073	0.199462
F	5.042868	-1.925023	-0.213728
F	4.510351	-3.125583	1.505615
F	4.566617	-4.024270	-0.457009
C	1.848901	-3.442086	-0.631673
H	2.040383	-4.444180	-0.990229
C	0.633049	-2.763103	-0.689004
C	-0.634800	-3.318385	-1.248728
F	-0.414754	-4.562557	-1.721564
F	-1.616164	-3.418912	-0.329673
F	-1.120155	-2.598229	-2.272518
N	0.765577	-1.527657	-0.123944
O	-2.014256	-0.543784	-0.628245
C	-3.106977	-0.065243	-0.078617
C	-4.148164	-0.965692	0.218317
H	-3.961132	-2.009148	-0.003585
C	-5.347345	-0.511860	0.768831
O	-6.379043	-1.305958	1.144204
C	-6.338501	-2.690835	0.854859
H	-5.333093	-3.106500	0.977606
H	-6.996151	-3.178204	1.576775
H	-6.667824	-2.880101	-0.173940
C	-5.576226	0.894612	0.997841
H	-6.513279	1.237625	1.425789
C	-4.590150	1.793867	0.666107
H	-4.766332	2.860483	0.777471
C	-3.323845	1.363594	0.172819
C	-2.358770	2.334278	-0.132585
H	-2.584678	3.389329	0.008554
N	-1.048122	2.107562	-0.265758
C	-0.206810	3.173773	-0.613730
C	0.920329	2.944004	-1.430103
H	1.088049	1.948779	-1.832558
C	1.799652	3.984437	-1.710389
H	2.662577	3.793892	-2.345248
C	1.593780	5.277736	-1.188361
C	2.605853	6.362291	-1.445221
H	3.587233	6.086807	-1.042845
H	2.307345	7.305237	-0.977594
H	2.727263	6.551154	-2.518174
C	0.460870	5.498691	-0.403801
H	0.262721	6.499828	-0.025657
C	-0.422199	4.466570	-0.093748
H	-1.276067	4.657751	0.548152

The infinite stacks in 1 and 3 crystals

Figure S22a shows the molecular infinite stack of the **1** crystal. The shown A-B couple of molecules highlights how the O^N ligand of one molecule is eclipsed to the N^N ligand of the second molecule. Furthermore, two donor atoms of one complex are close to the analogue ones of the second molecule. The C-D couple of molecules are symmetrically related to the A-B one, resulting from the application of an inversion operation. Molecules B and C face one another through their O^N ligands. The A-D sequence in Figure S22a is repeated and gives rise to an infinite stack, which could be suitable for charge conduction. No other infinite stacks can be found in the crystal organization of **1**. Its finding is a good clue in favor of efficient photogeneration and photoconduction. Figure S22b shows the possibly conductive stack in the **3** crystal. A sequence of four molecules is replicated so to produce, in principle, an infinite pattern which could be suitable for photogeneration and/or conduction. All the couples involve a good "facing" of the O^N ligand. Furthermore, couples A-B and C-D show a proximity to the central metals and its surrounding donor atoms. Such a stack is substantially formed by couples of almost planar molecules in close parallel relative disposition, as observed in the case of the **1** crystal.

Figure S22. (a) The infinite stack of parallel molecules in the studied **1** crystal. Couples A-B and C-D are repeated by translations along the b axis; (b) The linear stack observed in **3**.

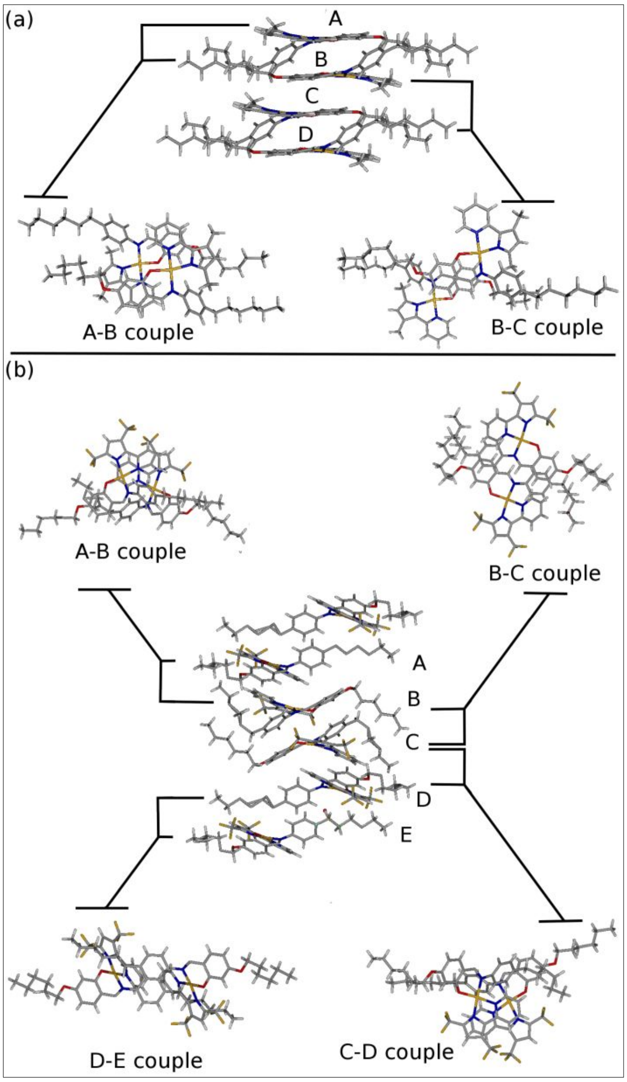

Table S12. Some computed properties of the lowest singlet and triplet excited states of **1**-**3**. All the structures were optimized in the corresponding excited state and in DCM solutions as detailed in the "Computational Methods" in the paper. The main character of the excited state is labelled as metal-centred (MC), metal-ligand charge transfer (MLCT) or ligand-ligand charge transfer (LLCT). The emission wavelength of each state is reported in nm.

State	Absolute Energy (kJ/mol)[a]	Emission wavelength (nm)	Excited state Assignment
		**1**	
**T_1**	0.0	641	MLCT
**T_2**	+18.4	599	MC (d_{z2}^*-d_{x2-y2}^*)
**S_1**	+57.6	477	MLCT
**S_2**	+62.9	476	MC (d_{π}^*-d_{x2-y2}^*)
**S_3**	+71.2	470	LLCT
		**2**	
**T_1**	0.0	634	MLCT
**T_2**	+7.4	542	LC (O^N ligand)
**S_1**	+60.5	478	MC (d_{π}^*-d_{x2-y2}^*)
**S_2**	+79.5	446	MLCT
**S_3**	+80.3	435	LLCT
		**3**	
**T_1**	0.00	600	LC
**T_2**	+3.6	524	MC (d_{π}^*-d_{x2-y2}^*)
**S_1**	+59.3	456	MC (d_{π}^*-d_{x2-y2}^*)
**S_2**	+87.1	412	MC (d_{z2}^*-d_{x2-y2}^*)
**S_3**	+107.5	380	LC

[a] Respect to T_1

Spin-Orbit perturbation of the lowest triplet states

Spin-orbit effects were computed on the lowest excited states of **1**-**3** complexes, on the basis of the TDDFT perturbative method [3] implemented in ADF 2019,[1] referred to as p-SOC (Perturbative Spin-Orbit Coupling) which combine low computational cost with limited reduction of accuracy.[4] This method has become one of the primary choices in transition metal complexes studies about spin-orbit coupled excited states and their dynamics.[5-7]

These computations were performed with the same mPW1PW91 xc functional used for Table S12 in the Gaussian09 software (see the "Computational Methods" paragraph for details), Zero Order Relativistic Approximation effects (ZORA) as implemented in ADF (default implementation) and ZORA/DZP basis set. Solvation was taken into account through the COSMO approximation, the chosen solvent was dichloromethane (as for Table S12). Default options and default parameters for the solvent were used. The numerical accuracy of these computations was set to "good" value (NUMERICALQUALITY=good keyword). The molecular structures were the same obtained with Gaussian 09 and used for Table S12 (also in this case, the interested reader is addressed to the main paper). The perturbative spin-orbit singlet-triplet couplings were computed through default options in ADF 2019 ("SOPERT" keywords without further specifications) associated to the computations of the first three triplet and first three singlet excited states.

Table S13 reports significant properties of the lowest six excited states. In particular, the percentage composition in terms of triplet and singlet sates (computed with the standard TDDFT method without spin-orbit corrections) clearly assigns the first three excited states to T_1, and the three next excited states to T_2. The excitation wavelength of the lowest two excited state, in particular, is very similar to the T_1 emission wavelength computed with Gaussian 09 in spite of the differences in the

computational methods applied through the two programs (in particular Slater basis sets and ZORA in ADF 2019 in comparison to the used relativistic pseudopotential and contracted Gaussian basis functions in Gaussian 09).
More important, no significant mixing is computed between triplet and singlet excited states up to the fourth excited state, in which a 5,9 % contribution (in **1**) comes from singlet states. In all the studied complexes, the lowest three excited states result from spin-orbit mixing mostly among pure triplet states components only. This makes the oscillator strengths of these excited states very small. These findings allow to explain the not-emissive character of the lowest (we could say T_1) excited state in all the complexes. Furthermore, the fact that no phosphorescent emission is observed also in solid solutions (see the discussions in the paper relative to Figure 7) cannot exclude the presence of relatively long-living triplet states, which could be not emissive because of their very low oscillator strength rather than fast non-radiative deactivation processes. A long lifetime of the lowest triplet state, combined to the unique nature of T_1 (a MLCT excited state), allows a direct explanation of the high observed photogeneration in **1**.

TABLE S13. Excited states of **1**-**3** computed with inclusion of spin-orbit effects. The lowest six excited states were computed on the T_1 optimized structure (optimized with Gaussian 09 as detailed in the paper about the computational study of emission in solid solution at 77 K, see also Table S12). Solvation effects (DCM) were included by the means of COSMO polarizable medium.

Excited State	Absolute Energy (kJ/mol)[a]	Emission wavelength (nm)	Oscillator Strength	Composition
		**1**		
1	0.0	631	$3\cdot10^{-7}$	T_1 81.3 %<br>T_2 18.5%
2	0.3	630	$2\cdot10^{-7}$	T_1 80.8 %<br>T_2 19.1%
3	6.6	610	$1\cdot10^{-6}$	T_1 99.2%<br>T_3 0.4 %
4	24.2	560	$2\cdot10^{-4}$	T_2 90.6%<br>S_1 5.9%<br>T_3 3.3%
5	31.5	541	$7\cdot10^{-7}$	T_2 70.5%<br>T_1 16.3 %<br>T_3 13.4%
6	33.1	537	$7\cdot10^{-6}$	T_2 79.1%<br>T_1 18.6%<br>T_3 1.5%<br>S_2 0.8 %
		**2**		
1	0.0	625	$1\cdot10^{-7}$	T_1 82.1 %<br>T_2 17.8%
2	0.0	624	$4\cdot10^{-6}$	T_1 82.0 %<br>T_2 17.9%
3	5.8	606	$6\cdot10^{-7}$	T_1 99.5%<br>T_3 0.4 %
4	24.4	554	$2\cdot10^{-4}$	T_2 90.1%<br>S_1 6.0%<br>S_2 0.3%<br>T_3 0.2%
5	31.5	536	$1\cdot10^{-6}$	T_2 70.1%<br>T_3 14.9%<br>T_1 12.8 %
6	33.0	532	$4\cdot10^{-6}$	T_2 66.5%<br>T_1 17.6%<br>T_3 1.8%<br>S_2 0.5%
		**3**		
1	0.0	604	$4\cdot10^{-7}$	T_1 62.7 %<br>T_2 35.1%<br>T_3 2.3%

2	0.8	602	$2{\cdot}10^{-6}$	T_1 63.0 %<br>T_2 36.7%<br>S_3 0.1%
3	6.0	587	$2{\cdot}10^{-4}$	T_1 83.1%<br>T_3 8.8 %<br>T_2 6.8 %<br>S_2 0.6 %<br>S_1 0.5 %
4	12.9	568	$1{\cdot}10^{-4}$	T_2 80.7%<br>T_1 9.7%<br>S_1 0.9%<br>S_2 0.4%
5	18.4	553	$2{\cdot}10^{-6}$	T_2 49.7%<br>T_1 26.5 %<br>T_3 23.8%
6	24.7	537	$2{\cdot}10^{-5}$	T_2 62.0%<br>T_1 36.0%<br>S_3 1.7%<br>T_3 0.1%

[a] Respect to Excitation 1, the structure is relaxed in T_1 without spin-orbit coupling and it is computed with Gaussian 09 (see the paper for details and discussions therein about Figure S12).

REFERENCES

1) ADF2019, SCM, Theoretical Chemistry, Vrije Universiteit, Amsterdam, The Netherlands, http://www.scm.com
2) Ziegler, T.; Rauk, A. On the calculation of bonding energies by the Hartree Fock Slater method. *Theor. Chim. Acta* **1977**, *46*, 1-10.
3) Wang, F.; Ziegler,T. A simplified relativistic time-dependent density-functional theory formalism for the calculations of excitation energies including spin-orbit coupling effect. *J. Chem. Phys* **2005**, *123*, 154102.
4) Moitra, T.; Karak, P.; Chakraborty, S.; Ruud, K.; Swapan Chakrabarti Behind the scenes of spin-forbidden decay pathways in transition metal complexes. *Phys. Chem. Chem. Phys*. **2021**, *23*, 59-81.
5) Paul, L.; Chakrabarti S.; Ruud, K. Anomalous Phosphorescence from an Organometallic White-Light Phosphor. *J. Phys. Chem. Lett.* **2017**, *8*, 4893-4897.
6) Moitra, T.; Alam, M. M.; Chakrabarti, S. Intersystem crossing rate dependent dual emission and phosphorescence from cyclometalated platinum complexes: a second order cumulant expansion based approach. *Phys. Chem. Chem. Phys.* **2018**, *20*, 23244–23251.
7) Mai, S.; Gattuso, H.; Fumanal, M.; Muñoz Losa, A.; Monari, A.; Daniel C.; González, L. Excited-states of a rhenium carbonyl diimine complex: solvation models, spin–orbit coupling, and vibrational sampling effects. *Phys. Chem. Chem. Phys.* **2017**, *19*, 27240-27250.
